# Supplementary material for: Investigating the effectiveness of school health services delivered by a health provider: A systematic review of systematic reviews
Source: PLoS One. 2019 Jun 12;14(6):e0212603. doi: 10.1371/journal.pone.0212603 (PMC6561551; doi:10.1371/journal.pone.0212603)
Supplement: S1 Appendix — (DOCX) [file pone.0212603.s001.docx]

**S1 APPENDIX. Protocol**

Finalized December 13, 2017

***Working title***

Investigating the effectiveness of school health services: a systematic review of systematic reviews

***Research question***

- Are school health services effective for improving the health of school-age children and adolescents?
  - *Population*: children (ages 5-9) and adolescents (ages 10-19) in school
  - *Intervention*: any form of school-based or school-linked health services that involves a health provider
  - *Comparator*: no school health services
  - *Outcome*: improved health outcomes (see list below)
- Sub-questions:
  - Based on information from the systematic reviews (SRs), what health areas do school health services address? Where are the gaps?
  - What is the quality of studies on school health services according to the SRs?
  - What is the quality of the SRs themselves?
  - To what extent are health providers involved in school health services?
  - How do school health services compare between high- and low-income countries or by region?
  - What is the cost-effectiveness of SHS?
  - Do gaps in health areas demonstrate existing services that are not well researched – or do gaps reveal services that are not included in most school health services?

***Aims***

- Through systematic searches in five databases, generate a table of SRs that fit specific inclusion criteria
- Determine what health areas are and are not addressed by SRs
  - This systematic review of systematic reviews (overview) will identify health areas and specific school health service interventions that will have at least some evidence on their effectiveness.
  - It will also suggest further research in areas where there is a recent review but it found insufficient evidence (gaps in the literature).
  - It will also identify health areas and specific school health service interventions for which no SRs were found. This might be because there are no studies or where there are studies but no SR has been conducted.
- Analyze the degree of effectiveness of interventions based on the findings of the SRs
- Where possible, conduct a meta-analysis of the results by health area (unlikely due to heterogeneous data)
- Propose an evidence-based package of services that could be offered in schools, based on these SRs, acknowledging that other evidence-based interventions might exist but have not been included in the SRs that have been found by our systematic searches.
  - How does this take into account different needs and resources of regions?

***Operational definitions***

- School health services: “Health services provided to enrolled students by health care and/or allied professional(s), irrespective of the site of service provision; the services should be mandated by a formal arrangement between the educational institution and the provider health care organization” [1].
- School health provider: “A health or allied professional who is involved in the provision of school health services” [1].
  - Deliverers considered health providers: clinical officers, counselors, dental assistants, dental professionals, dentists, dermatologists, dietitians, health extension workers/community health workers, health providers, immunization officers, medical practitioners, medical students, nurses (school, psychiatric, dental, public health, etc.), mental health professionals, mental health workers, nursing students, nutritionists, ophthalmologists, optometrists, paramedical staff, pediatricians, physicians, primary care providers, psychiatric social workers, psychiatrists, psychiatry trainees, psychologists (general, clinical, developmental, etc.), psychology graduate students, psychotherapists, refractionists, therapists (occupational, speech, art, etc.), trained health educators, etc.
  - Deliverers not considered health providers: parents/caregivers, printed materials/media, teachers (trained or not stated), youth (peers, youth workers, out of school youth)
  - Deliverers where it is unclear if they are health providers: graduate students (no specialty specified, specialty in non-medical field), research assistants, researchers, social workers
- School-based health services: “School health services that are provided in school premises; services can be provided by health care provider(s) located on-site, or visiting, or both” [1].
- School-linked health services: School health services that are provided in a healthcare facility or school health office that are not based in the school [2]
- School-age children and adolescents: ages 5-19

***Inclusion/exclusion criteria***:

*Types of SRs*:

- SRs are explicitly labeled “systematic review” within the title or abstract
- SRs have a methods section that outlines explicit inclusion criteria
- SRs are either of randomized controlled trials (RCTs), or RCTs and quasi-experimental studies (QEs), or RCTS and QEs and other non-randomized studies of interventions
- SRs assess at least one aspect of the effectiveness of one or more interventions provided in schools or for students enrolled in schools
- SRs are published in peer-reviewed journals
- SRs are published by June 15, 2018
- SRs are published in the English language
- Full texts of SRs are available

*Types of participants*:

- Children (5-9) or adolescents (10-19) enrolled in schools

*Types of interventions*:

- Interventions must involve a health provider (see definition above)
- Interventions must be school-based or school-linked
- Interventions must aim to improve some aspect of health for school-age children and/or adolescents
- Interventions must be compared to either no intervention, another intervention, or the same intervention in a different setting (e.g. community vs. school)
- Interventions may be of any duration

***Ranked exclusion criteria to standardize reasons for exclusion in full text screening****:*

1. Full text not available
2. Not published in a peer-reviewed journal
3. No studies included in the review
4. Full text not in English
5. Not labeled "systematic review" in title or abstract
6. Methods section does not include explicit inclusion/exclusion criteria
7. Not school-based or school-linked
8. Studies included are not randomized-controlled trials, controlled quasi-experimental studies, or other non-randomized controlled studies of interventions
9. No health provider explicitly involved
10. Participants are not between ages 5-19 (if no age given: must be clearly children/adolescents in school)

***Quality assessment:***

Before data extraction, SRs will be appraised on the following 4 points outlined by Ballard and Montgomery [3]:

1. Do SRs include primary trials that do not substantially overlap?
   1. Corrected covered area (CCA) to measure overlap [4]
2. Are reviews of high methodological quality?
   1. AMSTAR 2 to critically appraise quality of systematic reviews [5]
3. Are reviews up-to-date?
4. Do reviews match overview scope?

***Outcomes*** [6]:

This list will guide us in mapping outcomes included in reviews

1. Unintentional injury:
   1. Safety and accident prevention: incidence of traffic accidents or other accidents or injuries in school or at home
   2. Observation or self report of cycle-helmet use
2. Violence:
   1. Self-reported violence (for example, carried weapon, got into a fight)
   2. Bullying: self-reported incidence of being bullied or bullying others
3. Sexual and reproductive health (including HIV):
   1. Incidence of sexually transmitted infections (including HIV), pregnancy or abortion
   2. Self-reported use of condoms or other contraception, abstinence or delaying of sexual intercourse
   3. HPV vaccine coverage
4. Communicable diseases:
   1. Incidence of diseases such as diarrhea, respiratory tract infections such as cold or influenza, skin disease, worms, head lice
   2. Observation or self report of hand-washing with soap after visiting toilet or before handling food
   3. Vaccine coverage
5. Nutrition (including oral health) and physical activity:
   1. Self-reported food intake (particularly focusing on consumption of fruits and vegetables, water, high fat or sugar foods)
   2. Indicators of specific nutritional deficiencies (for example, iron, iodine, and vitamin A deficiencies)
   3. Body mass index or standardized body mass index (BMI or zBMI), height-for-age, weight-for-age, and weight-for-height z-scores, skin-fold thickness measures, waist circumference
   4. Student (or teacher or parent) reports of disordered eating habits, body size acceptance, self esteem
   5. Accelerometry, multi-stage fitness tests (for example, shuttle runs, step tests), self-reported levels of physical activity or sedentary behaviors
   6. Decayed, missing or filled teeth index
   7. Self-reported dental hygiene behaviors such as regular tooth brushing, dental check-ups
   8. Self-reported consumption of sugary snacks or drinks
6. Mental health, substance use & self-harm:
   1. Validated scales of well-being or quality of life or both
   2. Incidence of self harm or suicide
   3. Use of validated scales such as Rosenberg’s self esteem scale, Beck Depression Inventory, Strengths and Difficulties Questionnaire
   4. Tobacco use: salivary cotinine, carbon monoxide levels, self-reported use of cigarettes or other tobacco products
   5. Alcohol use: self-reported use of alcohol
   6. Other drug use: self-reported use of other drugs (legal or illegal)
7. Skin or sun safety:
   1. Observation or self report of sunscreen, behaviors to reduce exposure to the sun (for example, wearing hat, seeking shade, covering up)
