# Supplementary material for: Investigating the effectiveness of school health services delivered by a health provider: A systematic review of systematic reviews
Source: PLoS One. 2019 Jun 12;14(6):e0212603. doi: 10.1371/journal.pone.0212603 (PMC6561551; doi:10.1371/journal.pone.0212603)
Supplement: S2 Appendix — (DOCX) [file pone.0212603.s002.docx]

**S2 APPENDIX. Search strategy**

Finalized December 5, 2017

***PubMed***:

Filters:

*None*

Concept 1: school intervention

"School Health Services"[Mesh] OR school health service*[TI] OR health promoting school[TI] OR health promoting schools[TI] OR school-based health cent*[TI] OR school-based[TI] OR school-linked[TI] OR school nurs*[TI]

Concept 2: other interventions not obviously in schools

"Mass Screening"[Mesh] OR “Dental Care for Children”[Mesh] OR “Mass Vaccination”[Mesh] OR “Hygiene”[Mesh] OR “Health Promotion”[Mesh] OR screening*[TI] OR exam*[TI] OR deworming[TI] OR supplement*[TI] OR vaccin*[TI] OR hygiene[TI] OR dental[TI] OR promot*[TI] OR safety[TI] OR entry[TI] OR assessment[TI] OR chronic[TI] OR campaign[TI] OR emergenc*[TI] OR counselling[TI] OR counseling[TI] OR immuniz*[TI]

Concept 3: setting

("Schools"[Mesh:noexp] OR school[TIAB] OR schools[TIAB])

Concept 4: systematic review

“systematic review”[TIAB]

Combined:

(Concept 1 AND Concept 4) OR (Concept 2 AND Concept 3 AND Concept 4)

(("School Health Services"[Mesh] OR school health service*[TI] OR health promoting school[TI] OR health promoting schools[TI] OR school-based health cent*[TI] OR school-based[TI] OR school-linked[TI] OR school nurs*[TI]) AND “systematic review”[TIAB]) OR (("Mass Screening"[Mesh] OR “Dental Care for Children”[Mesh] OR “Mass Vaccination”[Mesh] OR “Hygiene”[Mesh] OR “Health Promotion”[Mesh] OR screening*[TI] OR exam*[TI] OR deworming[TI] OR supplement*[TI] OR vaccin*[TI] OR hygiene[TI] OR dental[TI] OR promot*[TI] OR safety[TI] OR entry[TI] OR assessment[TI] OR chronic[TI] OR campaign[TI] OR emergenc*[TI] OR counselling[TI] OR counseling[TI] OR immuniz*[TI]) AND ("Schools"[Mesh:noexp] OR school[TIAB] OR schools[TIAB]) AND “systematic review”[TIAB])

***Cochrane Library:***

Filters:

“Cochrane Reviews”

Concept 1: intervention- clearly in school

#1 "health promoting school" or "school-based health center" or "school-based" or "school-linked" or "school health services":ti (Word variations have been searched)

Concept 2: intervention- not clearly in school

#2 screening or exam or deworming or supplementation or vaccination or hygiene or dental or promotion or examination or safety or entry or assessment or chronic or campaign or emergency or counselling or immunization:ti (Word variations have been searched)

Concept 3: setting

#3 school:ti,ab,kw (Word variations have been searched)

Combined:

#1 or (#2 and #3)

*Web of Science:*

Filters:

“Reviews”

Concept 1: school intervention

#1 TI=("school health service*" OR “school nurs*” OR “health promoting school*” OR “school-based health cent*” OR “school-based” OR “school-linked”)

Concept 2: screening

#2 TI=(screening* or exam* or deworming or supplement* or vaccin* or hygiene or dental or promot* or safety or entry or assessment or chronic or campaign or emergenc* or counselling or counseling or immuniz*)

Concept 3: setting

#3 TS=(school OR schools)

Concept 4: systematic review

#4 TS=(“systematic review”)

Combined:

(#1 AND #4) OR (#2 AND #3 AND #4) AND DOCUMENT TYPES: (Review)

Indexes=SCI-EXPANDED, SSCI, A&HCI, CPCI-S, CPCI-SSH, ESCI Timespan=All years

***ERIC and psycINFO (via EBSCOhost)***

Filters:

“Academic journals”

Concept 1: population/setting

TI (“school health service*” OR "school nurs*" OR “health promoting school*” OR “school-based health cent*” OR “school-based” OR “school-linked”)

Concept 2: intervention

TI ( screening* OR exam* OR deworming OR supplement* OR vaccin* OR hygiene OR dental OR promot* OR safety OR entry OR assessment OR chronic OR campaign OR emergenc* OR counselling OR counseling OR immuniz*)

Concept 3: setting

TI (school OR schools ) OR AB ( school OR schools)

Concept 4: systematic review

TI "systematic review" OR AB "systematic review"

Combined:

(TI (“school health service*” OR "school nurs*" OR “health promoting school*” OR “school-based health cent*” OR “school-based” OR “school-linked”) AND (TI "systematic review" OR AB "systematic review")) OR (TI (screening* OR exam* OR deworming OR supplement* OR vaccin* OR hygiene OR dental OR promot* OR safety OR entry OR assessment OR chronic OR campaign OR emergenc* OR counselling OR counseling OR immuniz*) AND (TI (school OR schools) OR AB (school OR schools)) AND (TI "systematic review" OR AB "systematic review"))
