# Supplementary material for: Investigating the effectiveness of school health services delivered by a health provider: A systematic review of systematic reviews
Source: PLoS One. 2019 Jun 12;14(6):e0212603. doi: 10.1371/journal.pone.0212603 (PMC6561551; doi:10.1371/journal.pone.0212603)
Supplement: S3 Appendix — Table A. Excluded full text articles and reasons for exclusion. (DOCX) [file pone.0212603.s003.docx]

**S3 APPENDIX. Excluded full text articles.** Excluded full text articles and reasons for exclusion (Table A)

| **First author, year, reference** | **Reason for exclusion of full text** |
| --- | --- |
| Adair 2013 [7] | Not labeled "systematic review" in title or abstract |
| Adi 2007 [8] | Not published in a peer-reviewed journal |
| Adi 2007 [9] | Not published in a peer-reviewed journal |
| Agabio 2015 [10] | Less than 50% of studies within review fulfill all criteria |
| Ahmad 2011 [11] | Less than 50% of studies within review fulfill all criteria |
| Akers 2011 [12] | Unclear for at least 1 criterion |
| Allen 2016 [13] | Not labeled "systematic review" in title or abstract |
| Aloia 2016 [14] | No health provider involved |
| Altaf 2017 [15] | Studies included are not RCTs, QEs or other non-randomized studies |
| Amaugo 2014 [16] | No health provider involved |
| Anderson 2010 [17] | Unclear for at least 1 criterion |
| Arbesman 2013 [18] | Unclear for at least 1 criterion |
| Atilola 2016 [19] | Studies included are not RCTs, QEs or other non-randomized studies |
| Atkin 2011 [20] | Unclear for at least 1 criterion |
| Aveyard 2009 [21] | Not published in a peer-reviewed journal |
| Bains 2016 [22] | Less than 50% of studies within review fulfill all criteria |
| Bamford 2007 [23] | Studies included are not RCTs, QEs or other non-randomized studies |
| Barlow 1998 [24] | Less than 50% of studies within review fulfill all criteria |
| Barr-Anderson 2011 [25] | Unclear for at least 1 criterion |
| Barry 2013 [26] | Less than 50% of studies within review fulfill all criteria |
| Bayer 2009 [27] | Less than 50% of studies within review fulfill all criteria |
| Beets 2009 [28] | Less than 50% of studies within review fulfill all criteria |
| Bellamy 2005 [29] | Less than 50% of studies within review fulfill all criteria |
| Bennett 2015 [30] | Not labeled "systematic review" in title or abstract |
| Bennett 2005 [31] | Unclear for at least 1 criterion |
| Berkowitz 2007 [32] | Not published in a peer-reviewed journal |
| Bird 2017 [33] | Studies included are not RCTs, QEs or other non-randomized studies |
| Birdee 2009 [34] | No health provider involved |
| Blank 2010 [35] | Less than 50% of studies within review fulfill all criteria |
| Blank 2009 [36] | Not published in a peer-reviewed journal |
| Blank 2010 [37] | Unclear for at least 1 criterion |
| Borde 2017 [38] | Less than 50% of studies within review fulfill all criteria |
| Bothe 2006 [39] | Unclear for at least 1 criterion |
| Boyce 2017 [40] | No health provider involved |
| Brackney 2015 [41] | Less than 50% of studies within review fulfill all criteria |
| Brennan 2017 [42] | Studies included are not RCTs, QEs or other non-randomized studies |
| Bröning 2012 [43] | Less than 50% of studies within review fulfill all criteria |
| Brown 2014 [44] | Unclear for at least 1 criterion |
| Brown 2009 [45] | Less than 50% of studies within review fulfill all criteria |
| Burnim 2017 [46] | Unclear for at least 1 criterion |
| Cai 2014 [47] | Less than 50% of studies within review fulfill all criteria |
| Calear 2010 [48] | Less than 50% of studies within review fulfill all criteria |
| Camacho-Miñano 2011 [49] | Less than 50% of studies within review fulfill all criteria |
| Campbell 2000 [50] | Unclear for at least 1 criterion |
| Canter 2012 [51] | Unclear for at least 1 criterion |
| Cardoza 2012 [52] | Unclear for at least 1 criterion |
| Carlin 2016 [53] | Unclear for at least 1 criterion |
| Carney 2016 [54] | Unclear for at least 1 criterion |
| Carr 2007 [55] | Superseded by newer version |
| Carr 2012 [56] | Less than 50% of studies within review fulfill all criteria |
| Carroll 2012 [57] | Studies included are not RCTs, QEs or other non-randomized studies |
| Carson 2011 [58] | Less than 50% of studies within review fulfill all criteria |
| Cawley 2010 [59] | Less than 50% of studies within review fulfill all criteria |
| Chalamandaris 2015 [60] | Less than 50% of studies within review fulfill all criteria |
| Charania 2011 [61] | Unclear for at least 1 criterion |
| Cheney 2014 [62] | Less than 50% of studies within review fulfill all criteria |
| Chin 2012 [63] | Less than 50% of studies within review fulfill all criteria |
| Chong 2014 [64] | Superseded by newer version |
| Chong 2018 [65] | Unclear for at least 1 criterion |
| Clemmens 2004 [66] | Unclear for at least 1 criterion |
| Coffman 2009 [67] | Less than 50% of studies within review fulfill all criteria |
| Cooper Robbins 2011 [68] | Unclear for at least 1 criterion |
| Corcoran 2011 [69] | Not school-based or school-linked |
| Coren 2001 [70] | Unclear for at least 1 criterion |
| Cornell 2018 [71] | Less than 50% of studies within review fulfill all criteria |
| Corrieri 2014 [72] | Less than 50% of studies within review fulfill all criteria |
| Cuijpers 2002 [73] | Unclear for at least 1 criterion |
| Cuijpers 2006 [74] | Unclear for at least 1 criterion |
| Curran 2017 [75] | No health provider involved |
| Cushing 2014 [76] | Less than 50% of studies within review fulfill all criteria |
| Cusimano 2011 [77] | Less than 50% of studies within review fulfill all criteria |
| Dart 2018 [78] | Studies included are not RCTs, QEs or other non-randomized studies |
| Das 2016 [79] | Unclear for at least 1 criterion |
| Davó 2008 [80] | Full text not in English |
| Daykin 2008 [81] | Unclear for at least 1 criterion |
| De Bourdeaudhuij 2011 [82] | Unclear for at least 1 criterion |
| De Kleijn 2015 [83] | Less than 50% of studies within review fulfill all criteria |
| De Koker 2014 [84] | Less than 50% of studies within review fulfill all criteria |
| De Meester 2009 [85] | Unclear for at least 1 criterion |
| de Sa 2008 [86] | Less than 50% of studies within review fulfill all criteria |
| De-Regil 2011 [87] | Unclear for at least 1 criterion |
| Dean 2014 [88] | Less than 50% of studies within review fulfill all criteria |
| Delgado-Noguera 2011 [89] | Less than 50% of studies within review fulfill all criteria |
| Denno 2012 [90] | No health provider involved |
| Demetriou 2012 [91] | Unclear for at least 1 criterion |
| Demetriou 2015 [92] | Less than 50% of studies within review fulfill all criteria |
| DiCenso 2002 [93] | Less than 50% of studies within review fulfill all criteria |
| DiCenso 1999 [94] | Not published in a peer-reviewed journal |
| Dobbins 2009 [95] | Superseded by newer version |
| Dobbins 2013 [96] | Less than 50% of studies within review fulfill all criteria |
| Donaldson 1996 [97] | Not labeled "systematic review" in title or abstract |
| Driessen 2014 [98] | No health provider involved |
| Dudley 2015 [99] | Less than 50% of studies within review fulfill all criteria |
| Duperrex 2009 [100] | Unclear for at least 1 criterion |
| Eccleston 2002 [101] | Less than 50% of studies within review fulfill all criteria |
| Edwards 2014 [102] | Less than 50% of studies within review fulfill all criteria |
| Ejemot-Nwadiaro 2015 [103] | Less than 50% of studies within review fulfill all criteria |
| Ekeland 2004 [104] | Less than 50% of studies within review fulfill all criteria |
| Eklund 2018 [105] | Unclear for at least 1 criterion |
| El Dib 2008 [106] | Unclear for at least 1 criterion |
| Elder 2005 [107] | Unclear for at least 1 criterion |
| Elliott 2005 [108] | Unclear for at least 1 criterion |
| Evans 2012 [109] | Unclear for at least 1 criterion |
| Evans 2014 [110] | Unclear for at least 1 criterion |
| Faggiano 2008 [111] | No health provider involved |
| Faggiano 2014 [112] | Unclear for at least 1 criterion |
| Fair 2018 [113] | Unclear for at least 1 criterion |
| Farahmand 2011 [114] | Less than 50% of studies within review fulfill all criteria |
| Farrington 2009 [115] | Less than 50% of studies within review fulfill all criteria |
| Feldman 2014 [116] | Unclear for at least 1 criterion |
| Feng 2017 [117] | Unclear for at least 1 criterion |
| Flay 2009 [118] | Less than 50% of studies within review fulfill all criteria |
| Flynn 2015 [119] | Unclear for at least 1 criterion |
| Fonner 2014 [120] | Less than 50% of studies within review fulfill all criteria |
| Fortnum 2016 [121] | Unclear for at least 1 criterion |
| Foster 2018 [122] | Unclear for at least 1 criterion |
| Fothergill 2003 [123] | No studies included in the review |
| Fowden 2016 [124] | Studies included are not RCTs, QEs or other non-randomized studies |
| Foxcroft 2003 [125] | Unclear for at least 1 criterion |
| Foxcroft 1997 [126] | Unclear for at least 1 criterion |
| Foxcroft 2012 [127] | Less than 50% of studies within review fulfill all criteria |
| Foxcroft 2011 [128] | Unclear for at least 1 criterion |
| Franklin 1997 [129] | Not labeled "systematic review" in title or abstract |
| Galantino 2008 [130] | Unclear for at least 1 criterion |
| Gallagher 2016 [131] | Unclear for at least 1 criterion |
| Gambhir 2013 [132] | Unclear for at least 1 criterion |
| Gao 2014 [133] | Unclear for at least 1 criterion |
| Gascoine 2017 [134] | Studies included are not RCTs, QEs or other non-randomized studies |
| Gavin 2010 [135] | Less than 50% of studies within review fulfill all criteria |
| Gavine 2016 [136] | Less than 50% of studies within review fulfill all criteria |
| Gera 2018 [137] | Less than 50% of studies within review fulfill all criteria |
| Glaser 2016 [138] | Unclear for at least 1 criterion |
| Godin 2015 [139] | Unclear for at least 1 criterion |
| Gonzalez-Suarez 2008 [140] | No studies included in the review |
| Gorga 2016 [141] | Less than 50% of studies within review fulfill all criteria |
| Gould 2016 [142] | No health provider involved |
| Govindasamy 2015 [143] | Unclear for at least 1 criterion |
| Griebler 2017 [144] | No health provider involved |
| Griffin 2017 [145] | Unclear for at least 1 criterion |
| Haddison 2017 [146] | Less than 50% of studies within review fulfill all criteria |
| Hahn 2007 [147] | Unclear for at least 1 criterion |
| Hale 2014 [148] | Less than 50% of studies within review fulfill all criteria |
| Harden 2001 [149] | Not published in a peer-reviewed journal |
| Harden 2009 [150] | No health provider involved |
| Harrington 1998 [151] | Not school-based or school-linked |
| Harris 2009 [152] | Unclear for at least 1 criterion |
| Harrison 2010 [153] | Less than 50% of studies within review fulfill all criteria |
| Hartmann-Boyce 2014 [154] | Not labeled "systematic review" in title or abstract |
| Hawton 2000 [155] | Not school-based or school-linked |
| Heerde 2018 [156] | Studies included are not RCTs, QEs or other non-randomized studies |
| Hegarty 2016 [157] | No health provider involved |
| Hersch 2014 [158] | Unclear for at least 1 criterion |
| Hillier-Brown 2014 [159] | Unclear for at least 1 criterion |
| Hodder 2017 [160] | Unclear for at least 1 criterion |
| Hoehner 2013 [161] | Unclear for at least 1 criterion |
| Hoehner 2008 [162] | Unclear for at least 1 criterion |
| Hollis 2016 [163] | Unclear for at least 1 criterion |
| Holly 2018 [164] | Unclear for at least 1 criterion |
| Holub 2013 [165] | Less than 50% of studies within review fulfill all criteria |
| Holub 2014 [166] | Less than 50% of studies within review fulfill all criteria |
| Hoyland 2009 [167] | Unclear for at least 1 criterion |
| Hynynen 2016 [168] | Less than 50% of studies within review fulfill all criteria |
| Ickes 2013 [169] | Unclear for at least 1 criterion |
| Ingram 2011 [170] | Unclear for at least 1 criterion |
| Isaac 2009 [171] | Unclear for at least 1 criterion |
| Isensee 2012 [172] | Unclear for at least 1 criterion |
| Jackson 2012 [173] | Unclear for at least 1 criterion |
| Jackson 2010 [174] | Not published in a peer-reviewed journal |
| Jacob 2016 [175] | Less than 50% of studies within review fulfill all criteria |
| Jacobson Vann 2018 [176] | Less than 50% of studies within review fulfill all criteria |
| Jago 2004 [177] | No health provider involved |
| Jamil 2014 [178] | Unclear for at least 1 criterion |
| Jenkinson 2014 [179] | No health provider involved |
| Jordans 2009 [180] | Studies included are not RCTs, QEs or other non-randomized studies |
| Joury 2017 [181] | Less than 50% of studies within review fulfill all criteria |
| Joury 2017 [182] | Duplicate |
| Kahn 2002 [183] | Unclear for at least 1 criterion |
| Kamath 2008 [184] | Unclear for at least 1 criterion |
| Kang 2009 [185] | Not labeled "systematic review" in title or abstract |
| Katz 2013 [186] | Less than 50% of studies within review fulfill all criteria |
| Katz 2009 [187] | Not labeled "systematic review" in title or abstract |
| Katz 2008 [188] | Unclear for at least 1 criterion |
| Kay 1998 [189] | Methods section does not contain explicit inclusion/exclusion criteria |
| Keen 2017 [190] | Unclear for at least 1 criterion |
| Kellou 2014 [191] | Unclear for at least 1 criterion |
| Kelly 2017 [192] | Unclear for at least 1 criterion |
| Kendrick 2012 [193] | No health provider involved |
| Kessels 2012 [194] | Studies included are not RCTs, QEs or other non-randomized studies |
| Khayyati 2015 [195] | Unclear for at least 1 criterion |
| Kim 1997 [196] | Less than 50% of studies within review fulfill all criteria |
| Kingsnorth 2007 [197] | Unclear for at least 1 criterion |
| Kirkland 2018 [198] | Duplicate |
| Kirkland 2017 [199] | Unclear for at least 1 criterion |
| Knai 2006 [200] | Unclear for at least 1 criterion |
| Knopf 2016 [201] | Less than 50% of studies within review fulfill all criteria |
| Knowlden 2013 [202] | Unclear for at least 1 criterion |
| Kong 2016 [203] | Unclear for at least 1 criterion |
| Kothandan 2014 [204] | Unclear for at least 1 criterion |
| Krishnaswami 2012 [205] | Unclear for at least 1 criterion |
| Kristjansson 2007 [206] | Less than 50% of studies within review fulfill all criteria |
| Kropski 2008 [207] | Unclear for at least 1 criterion |
| Lösel 2003 [208] | Unclear for at least 1 criterion |
| Lai 2014 [209] | Unclear for at least 1 criterion |
| Laine 2014 [210] | Studies included are not RCTs, QEs or other non-randomized studies |
| Lane 2016 [211] | Unclear for at least 1 criterion |
| Langford 2015 [212] | Unclear for at least 1 criterion |
| Langford 2014 [213] | Less than 50% of studies within review fulfill all criteria |
| Larun 2006 [214] | Unclear for at least 1 criterion |
| Lavelle 2012 [215] | Unclear for at least 1 criterion |
| Lee 2016 [216] | Unclear for at least 1 criterion |
| Lee 2016 [217] | Less than 50% of studies within review fulfill all criteria |
| Leff 1999 [218] | No studies included in the review |
| Lemstra 2010 [219] | Unclear for at least 1 criterion |
| Leroy 2017 [220] | Less than 50% of studies within review fulfill all criteria |
| Lew 2015 [221] | Studies included are not RCTs, QEs or other non-randomized studies |
| Li 2008 [222] | Less than 50% of studies within review fulfill all criteria |
| Li 2015 [223] | Less than 50% of studies within review fulfill all criteria |
| Li 2015 [224] | Duplicate |
| Lima-Serrano 2014 [225] | Less than 50% of studies within review fulfill all criteria |
| Limbos 2007 [226] | Unclear for at least 1 criterion |
| Lindsay 2015 [227] | Studies included are not RCTs, QEs or other non-randomized studies |
| Lineberry 2015 [228] | Less than 50% of studies within review fulfill all criteria |
| Lister-Sharp 1999 [229] | Less than 50% of studies within review fulfill all criteria |
| Liu 2015 [230] | Unclear for at least 1 criterion |
| Lobelo 2013 [231] | Less than 50% of studies within review fulfill all criteria |
| Lofton 2016 [232] | Unclear for at least 1 criterion |
| Loharikar 2018 [233] | Studies included are not RCTs, QEs or other non-randomized studies |
| Loke 2017 [234] | Unclear for at least 1 criterion |
| Lopez 2016 [235] | Unclear for at least 1 criterion |
| Lopez 2016 [236] | Less than 50% of studies within review fulfill all criteria |
| Lopez 2013 [237] | Less than 50% of studies within review fulfill all criteria |
| Low 2013 [238] | Unclear for at least 1 criterion |
| Lynas 2017 [239] | Unclear for at least 1 criterion |
| Lytle 1995 [240] | Not published in a peer-reviewed journal |
| Macgowan 2004 [241] | Unclear for at least 1 criterion |
| Mann 2005 [242] | Unclear for at least 1 criterion |
| Mariani 2015 [243] | Studies included are not RCTs, QEs or other non-randomized studies |
| Marinho 2016 [244] | Less than 50% of studies within review fulfill all criteria |
| Marques 2017 [245] | Unclear for at least 1 criterion |
| Marseille 2018 [246] | Less than 50% of studies within review fulfill all criteria |
| Mason-Jones 2012 [247] | Less than 50% of studies within review fulfill all criteria |
| Mason-Jones 2016 [248] | Less than 50% of studies within review fulfill all criteria |
| Matangila 2015 [249] | Unclear for at least 1 criterion |
| Maxwell 2008 [250] | Not labeled "systematic review" in title or abstract |
| Mbakaya 2017 [251] | Unclear for at least 1 criterion |
| Mbuagbaw 2012 [252] | Not school-based or school-linked |
| McBride 2003 [253] | Unclear for at least 1 criterion |
| McClain 2018 [254] | Unclear for at least 1 criterion |
| Mcdaid 2011 [255] | Unclear for at least 1 criterion |
| McDonald 2018 [256] | Unclear for at least 1 criterion |
| McGinnis 2017 [257] | Unclear for at least 1 criterion |
| McGuinness 2018 [258] | No health provider involved |
| McQueston 2013 [259] | Less than 50% of studies within review fulfill all criteria |
| Meade 2005 [260] | Unclear for at least 1 criterion |
| Meadows 2004 [261] | Less than 50% of studies within review fulfill all criteria |
| Medina-Blanco 2011 [262] | Full text not in English |
| Mellor 2014 [263] | Less than 50% of studies within review fulfill all criteria |
| Merry 2004 [264] | Less than 50% of studies within review fulfill all criteria |
| Metcalf 2012 [265] | Unclear for at least 1 criterion |
| Michielsen 2010 [266] | Less than 50% of studies within review fulfill all criteria |
| Minatto 2016 [267] | Less than 50% of studies within review fulfill all criteria |
| Mirzazadeh 2017 [268] | Unclear for at least 1 criterion |
| Mirzazadeh 2018 [269] | Duplicate |
| Montoya 2003 [270] | Not labeled "systematic review" in title or abstract |
| Moore 2015 [271] | Unclear for at least 1 criterion |
| Mura 2015 [272] | Unclear for at least 1 criterion |
| Murray 2007 [273] | Less than 50% of studies within review fulfill all criteria |
| Mytton 2002 [274] | Superseded by newer version |
| Mytton 2006 [275] | Less than 50% of studies within review fulfill all criteria |
| Napierala Mavedzenge 2011 [276] | No health provider involved |
| National Health and Medical Research Council 2002 [277] | Not published in a peer-reviewed journal |
| Naylor 2015 [278] | Unclear for at least 1 criterion |
| Negrini, 2005 [279] | Not labeled "systematic review" in title or abstract |
| Neil 2007 [280] | Unclear for at least 1 criterion |
| Niccolai 2015 [281] | Less than 50% of studies within review fulfill all criteria |
| Niemeier 2012 [282] | Unclear for at least 1 criterion |
| Nishio 2018 [283] | Unclear for at least 1 criterion |
| Nixon 2012 [284] | Unclear for at least 1 criterion |
| Nixon 2012 [285] | Unclear for at least 1 criterion |
| Nogueira 2014 [286] | Unclear for at least 1 criterion |
| Nordheim 2016 [287] | Unclear for at least 1 criterion |
| Norris 2015 [288] | Unclear for at least 1 criterion |
| Oakley 1995 [289] | Full text not available |
| Ogilvie 2007 [290] | No health provider involved |
| Onate 2016 [291] | Unclear for at least 1 criterion |
| Onrust 2016 [292] | Unclear for at least 1 criterion |
| Oosterhoff 2016 [293] | Unclear for at least 1 criterion |
| Oringanje 2009 [294] | Less than 50% of studies within review fulfill all criteria |
| Orton 2016 [295] | Less than 50% of studies within review fulfill all criteria |
| Owen 2010 [296] | Less than 50% of studies within review fulfill all criteria |
| Owen 2017 [297] | Less than 50% of studies within review fulfill all criteria |
| Owen 2011 [298] | Unclear for at least 1 criterion |
| Perez Lopez 2015 [299] | Full text not in English |
| Pérez-Morales 2009 [300] | Full text not in English |
| Paleg 2015 [301] | Unclear for at least 1 criterion |
| Park 2006 [302] | Unclear for at least 1 criterion |
| Parrish 2013 [303] | Unclear for at least 1 criterion |
| Passon 2011 [304] | Full text not in English |
| Pattison 2006 [305] | Less than 50% of studies within review fulfill all criteria |
| Pearson 2015 [306] | Unclear for at least 1 criterion |
| Pearson 2015 [307] | Not labeled "systematic review" in title or abstract |
| Perman 2017 [308] | Studies included are not RCTs, QEs or other non-randomized studies |
| Persson 2009 [309] | Not labeled "systematic review" in title or abstract |
| Petering 2014 [310] | No health provider involved |
| Picot 2012 [311] | Less than 50% of studies within review fulfill all criteria |
| Powell 2004 [312] | Superseded by newer version |
| Pozuelo-Carrascosa 2017 [313] | Unclear for at least 1 criterion |
| Pucher 2013 [314] | Unclear for at least 1 criterion |
| Quitério 2013 [315] | Less than 50% of studies within review fulfill all criteria |
| Rabin 2010 [316] | Unclear for at least 1 criterion |
| Racey 2016 [317] | Unclear for at least 1 criterion |
| Rafferty 2016 [318] | Unclear for at least 1 criterion |
| Ran 2016 [319] | Unclear for at least 1 criterion |
| Raphael 2013 [320] | No health provider involved |
| Rasberry 2011 [321] | Unclear for at least 1 criterion |
| Renzaho 2010 [322] | Unclear for at least 1 criterion |
| Resnicow 1993 [323] | Not labeled "systematic review" in title or abstract |
| Ribeiro 2010 [324] | Unclear for at least 1 criterion |
| Rice 2016 [325] | Unclear for at least 1 criterion |
| Richards 2008 [326] | Not labeled "systematic review" in title or abstract |
| Ring 2007 [327] | Less than 50% of studies within review fulfill all criteria |
| Roberts 2001 [328] | Unclear for at least 1 criterion |
| Robinson 2013 [329] | Less than 50% of studies within review fulfill all criteria |
| Rojas-Andrade 2018 [330] | Unclear for at least 1 criterion |
| Royal 2007 [331] | Unclear for at least 1 criterion |
| Sabirin 2010 [332] | Studies included are not RCTs, QEs or other non-randomized studies |
| Salerno 2016 [333] | Less than 50% of studies within review fulfill all criteria |
| Sancassiani 2015 [334] | No health provider involved |
| Sani 2016 [335] | Less than 50% of studies within review fulfill all criteria |
| Saraf 2012 [336] | Unclear for at least 1 criterion |
| Saraiya 2004 [337] | Unclear for at least 1 criterion |
| Sbruzzi 2013 [338] | Less than 50% of studies within review fulfill all criteria |
| Schachter 2008 [339] | Unclear for at least 1 criterion |
| Scher 2006 [340] | Less than 50% of studies within review fulfill all criteria |
| Selkie 2016 [341] | Studies included are not RCTs, QEs or other non-randomized studies |
| Setiawan 2017 [342] | Not school-based or school-linked |
| Sexton 1996 [343] | Unclear for at least 1 criterion |
| Shepherd 2010 [344] | Less than 50% of studies within review fulfill all criteria |
| Sherman 2009 [345] | Unclear for at least 1 criterion |
| Shucksmith 2010 [346] | Not labeled "systematic review" in title or abstract |
| Sichieri 2014 [347] | Unclear for at least 1 criterion |
| Silveira 2011 [348] | Full text not in English |
| Silveira 2013 [349] | Unclear for at least 1 criterion |
| Singh 2012 [350] | Unclear for at least 1 criterion |
| Sipsma 2015 [351] | Less than 50% of studies within review fulfill all criteria |
| Skeie 2018 [352] | Unclear for at least 1 criterion |
| Sobol-Goldberg 2013 [353] | Unclear for at least 1 criterion |
| Soole 2008 [354] | Less than 50% of studies within review fulfill all criteria |
| Speizer 2003 [355] | Not labeled "systematic review" in title or abstract |
| Stanley 2015 [356] | Less than 50% of studies within review fulfill all criteria |
| Stead 1996 [357] | Not labeled "systematic review" in title or abstract |
| Steele 2006 [358] | Less than 50% of studies within review fulfill all criteria |
| Stein 2018 [359] | Duplicate |
| Stein 2018 [360] | Duplicate |
| Stewart 2012 [361] | Less than 50% of studies within review fulfill all criteria |
| Stothard 2013 [362] | Unclear for at least 1 criterion |
| Summerbell 2012 [363] | Not labeled "systematic review" in title or abstract |
| Sun 2013 [364] | Unclear for at least 1 criterion |
| Suthar 2013 [365] | Studies included are not RCTs, QEs or other non-randomized studies |
| Sutton 2014 [366] | Unclear for at least 1 criterion |
| Sutton 2018 [367] | Unclear for at least 1 criterion |
| Szumilas 2011 [368] | Less than 50% of studies within review fulfill all criteria |
| Taylor-Robinson 2015 [369] | Unclear for at least 1 criterion |
| Teesson 2012 [370] | Unclear for at least 1 criterion |
| Thakore 2015 [371] | Studies included are not RCTs, QEs or other non-randomized studies |
| Thomas 2002 [372] | Superseded by newer version |
| Thomas 2004 [373] | Not published in a peer-reviewed journal |
| Thomas 2006 [374] | Superseded by newer version |
| Thomas 2015 [375] | Less than 50% of studies within review fulfill all criteria |
| Thomas 2000 [376] | Not labeled "systematic review" in title or abstract |
| Thomas 2013 [377] | Less than 50% of studies within review fulfill all criteria |
| Tilford 1997 [378] | Full text not available |
| Tol 2011 [379] | Unclear for at least 1 criterion |
| Tollit 2018 [380] | Unclear for at least 1 criterion |
| Topping 2009 [381] | Less than 50% of studies within review fulfill all criteria |
| Ttofi 2011 [382] | Unclear for at least 1 criterion |
| Ttofi 2009 [383] | Unclear for at least 1 criterion |
| Turner 2005 [384] | Unclear for at least 1 criterion |
| Uijtdewilligen 2016 [385] | Less than 50% of studies within review fulfill all criteria |
| Underhill 2008 [386] | Less than 50% of studies within review fulfill all criteria |
| Underhill 2007 [387] | Less than 50% of studies within review fulfill all criteria |
| Vezina-Im 2017 [388] | Less than 50% of studies within review fulfill all criteria |
| Van Cauwenberghe 2010 [389] | Less than 50% of studies within review fulfill all criteria |
| Van Lippevelde 2012 [390] | Unclear for at least 1 criterion |
| van Sluijs 2007 [391] | Duplicate |
| van Sluijs 2007 [392] | Less than 50% of studies within review fulfill all criteria |
| van Stralen 2011 [393] | Unclear for at least 1 criterion |
| Verrotti 2014 [394] | Less than 50% of studies within review fulfill all criteria |
| Verstraeten 2012 [395] | Less than 50% of studies within review fulfill all criteria |
| Villa-Gonzalez 2018 [396] | Unclear for at least 1 criterion |
| Vindigni 2011 [397] | Unclear for at least 1 criterion |
| Vreeman 2007 [398] | Unclear for at least 1 criterion |
| Waddell 2007 [399] | Less than 50% of studies within review fulfill all criteria |
| Wainwright 2000 [400] | No studies included in the review |
| Walsh 2018 [401] | Duplicate |
| Walsh 2015 [402] | Superseded by newer version |
| Walter 2015 [403] | Not labeled "systematic review" in title or abstract |
| Wang 2013 [404] | Less than 50% of studies within review fulfill all criteria |
| Wang 2018 [405] | Unclear for at least 1 criterion |
| Wang 2015 [406] | Less than 50% of studies within review fulfill all criteria |
| Wang 2018 [407] | Duplicate |
| Warren-Gash 2013 [408] | Unclear for at least 1 criterion |
| Waters 2011 [409] | Less than 50% of studies within review fulfill all criteria |
| Watson 2017 [410] | Unclear for at least 1 criterion |
| Weisz 1987 [411] | Not labeled "systematic review" in title or abstract |
| Wells 2003 [412] | Less than 50% of studies within review fulfill all criteria |
| Werch 2002 [413] | Not labeled "systematic review" in title or abstract |
| Westwood 2007 [414] | Studies included are not RCTs, QEs or other non-randomized studies |
| Whitaker 2006 [415] | Less than 50% of studies within review fulfill all criteria |
| Whitaker 2013 [416] | Unclear for at least 1 criterion |
| White 1998 [417] | Unclear for at least 1 criterion |
| Wiefferink 2006 [418] | Studies included are not RCTs, QEs or other non-randomized studies |
| Wiehe 2015 [419] | Unclear for at least 1 criterion |
| Wight 2013 [420] | Unclear for at least 1 criterion |
| Willmott 2016 [421] | Less than 50% of studies within review fulfill all criteria |
| Wilson 2006 [422] | Unclear for at least 1 criterion |
| Wilson 2007 [423] | Unclear for at least 1 criterion |
| Wood 2006 [424] | Less than 50% of studies within review fulfill all criteria |
| Wood 2012 [425] | Unclear for at least 1 criterion |
| Worrell 2015 [426] | Unclear for at least 1 criterion |
| Yamada 1999 [427] | Full text not available |
| Yeung 2015 [428] | Not school-based or school-linked |
| Yildirim 2011 [429] | Unclear for at least 1 criterion |
| Young 2015 [430] | Unclear for at least 1 criterion |
| Zwi 2007 [431] | Less than 50% of studies within review fulfill all criteria |
