# Supplementary material for: Investigating the effectiveness of school health services delivered by a health provider: A systematic review of systematic reviews
Source: PLoS One. 2019 Jun 12;14(6):e0212603. doi: 10.1371/journal.pone.0212603 (PMC6561551; doi:10.1371/journal.pone.0212603)
Supplement: S4 Appendix — Table B. Quality appraisal of primary studies within included systematic reviews. (DOCX) [file pone.0212603.s004.docx]

**S4 APPENDIX. Quality appraisal of primary studies.** Quality appraisal of primary studies within included SRs (Table B).

| First author, reference | Method/tool used | Quality/risk of bias findings |
| --- | --- | --- |
| Arora [432] | Cochrane collaboration risk of bias tool [433] | “We assessed two of the six trials as low risk of bias (Milsom 2006; Cunningham 2009); one trial as high risk of bias (Hebbal 2005); and three trials as unclear risk (Zarod 1992; Burden 1994; Praveen 2014)… Allocation: Only two of the included trials adequately reported the method of sequence generation (Milsom 2006; Cunningham 2009); and three adequately reported concealment of allocation (Burden 1994; Milsom 2006; Cunningham 2009). The studies other than Cunningham 2009 and Milsom 2006 were at unclear risk of selection bias. Blinding: Out of six included trials, blinding of participants and personnel was not reported in two trials, which we therefore considered to have an unclear risk of performance and detection bias (Praveen 2014; Zarod 1992). Blinding of participants was not done in one trial so we judged it to be at high risk of performance bias, though a computer programme assessed the main outcome ’school attendance’ so we considered the study to be at low risk of detection bias (Hebbal 2005). Three trials described blinding of participants and assessors and we assessed them to be at low risk of performance or detection bias (Burden 1994; Milsom 2006; Cunningham 2009). Incomplete outcome data: In this review, not attending or not registering at the dental surgery is an outcome measure rather than attrition. Hence, we redefined attrition bias for this systematic review as ’parents not receiving call letters from school’. Based on this definition, all six trials were at low risk of attrition bias based on the data presented. Selective reporting: All the six included trials were at low risk of reporting bias as all pre-stated outcomes in the methods were reported.” |
| Bastounis [434] | Cochrane collaboration risk of bias tool [435] | “Most of the RCTs were of high quality. In two of the studies (Gillham et al., 2007; Roberts et al., 2010), it was not clear whether clusters ICC estimation was considered in the analysis. These studies were rated as high-risk in the section of “other bias”. Studies’ characteristics related to allocation procedures were taken into account especially for individual RCTs. Given the nature of intervention and control conditions, a complete blinding procedure was impossible; however, given the type of assessment (self-report questionnaires) most of the studies were rated as low-risk in “blinding” sections of the risk of bias tool. Protocols of RCTs were obtained, where it was possible, in order to assess the fidelity between protocols and trials reported results. Evidence of selective reporting resulted in rating these studies as high-risk in “selective reporting” section of risk of bias tool. Studies, which followed an intention-to-treat analysis, were rated as low-risk… Most of the cluster RCTs were rated as high quality. Most of the individual RCTs did not adequately report design and randomization procedures, leading to an inflated risk of bias scores.” |
| Brendel [436] | Cochrane collaboration risk of bias tool [433] | “The included study used a pretest–posttest quasi-experimental design; potential biases in non-randomized designs are likely to be greater than randomized studies. Risk of bias was assessed for selection, performance, detection, attrition, and reporting biases using the Cochrane Collaboration’s tool for assessing risk of bias (Higgins et al., 2011). Selection bias was rated as high risk due to differences between the control and treatment groups on baseline characteristics. In addition, the military parent of six children in the treatment group never deployed and six returned from deployment prior to posttest, whereas all of the military parents of the control group children deployed and only two returned from deployment prior to posttest. Performance bias and detection bias were rated as high risk as all participants, providers, and the investigator were aware of group assignment. Attrition bias was rated as high risk on the Child Behavior Checklist (CBCL) outcome due to high overall (31%) and differential (10%) attrition resulting from teachers not returning the CBCL questionnaire at posttest. Low risk of attrition bias was present for the self-esteem and anxiety outcomes due to no reported attrition. The study author reported data on all outcomes, thus reporting bias was rated as low risk.” |
| Chung [437] | Cochrane collaboration risk of bias tool [435] | “As all studies were comparing intervention and no intervention, they were rated to have high risk of bias in the blinding of participants and personnel. Three of the 7 included studies were rated to have high risk of bias due to the selective recruitment of cluster members; whereas 1 study had high risk of bias due to incomplete outcome data. All studies did not report whether there was blinding of outcome assessment, 6 studies did not report whether allocation concealment was performed, and 4 studies did not report whether random-sequence generation was arranged.” |
| Cooper [438] | Cochrane collaboration risk of bias tool [433] | “Across the domains relating to selection, performance and detection bias, the majority of trials scored low or unclear. Selective reporting bias was deemed to be highest risk of bias, being scored as high for half of the included studies. Overall the risk of bias was assessed as unclear in Zanin 2007, and high in the remaining three studies.” |
| Evans [439] | Cochrane collaboration risk of bias tool [433] | “Overall we judged the studies to be at low risk of bias… Most of the trials described an adequate method of generating the random sequence… We judged all seven studies as having adequate allocation concealment…We judged all the studies to be at low risk of performance bias… Five out of the seven studies reported efforts to mask outcome assessment (Morjaria 2016; RECS 2009; SIL 2014; SIL II 2015; WEAR 2017). In Wedner 2008 this was not clearly described. Congdon 2011 did not mask the outcome assessments but any bias would have been expected to favour the intervention (education), which was not the case… Follow-up was high and reasonably balanced between groups in most studies (6) and we judged these to be at low risk of attrition bias… Selective reporting was harder to judge… Two studies reported all pre-planned outcomes (Morjaria 2016; Wedner 2008), other studies did not report all pre-planned outcomes but the missing out- comes were not relevant to the review (SIL 2014; SIL II 2015; WEAR 2017). Two studies did not report some of our pre-specified review outcomes. Congdon 2011 did not report the prevalence of refractive error at six months and RECS 2009 did not report spectacle use at 6 to 12 months… For the cluster-randomised controlled trials only (Congdon 2011; SIL 2014; SIL II 2015; Wedner 2008) we considered three additional potential sources of bias. Baseline imbalance: Baseline data were poorly reported at the cluster level but individual-level data were available that largely suggested no major imbalances in these trials. Only SIL 2014 provided enough information to be confident that there were no baseline imbalances. Loss of clusters: Again there was no strong evidence that this was a problem but only two studies provided enough information to judge definitively (SIL 2014; SIL II 2015). Recruitment bias: Although this was not addressed directly the trials had made efforts to mask treatment assignment and we felt that recruitment bias was unlikely in a school setting.” |
| Geryk [440] | Not stated | Not stated |
| Gold [441] | Assessment made, but tool not specified | “Randomisation: Two studies (Buday 1995, Farmer 2003) stated explicitly that randomisation was used to assign participants to conditions. The remaining study (Brownell 2002) used the term ‘counterbalanced’ to describe an assignment that was either random or quasi-random, but intended to be random. Methods of randomisation and allocation concealment were not specified in the studies. Blindness and quality of outcome assessment: There was one single-blind study, with blinded assessors (Buday 1995). It was not reported whether blindness was used in the other studies. All studies used independent raters to assess outcomes. All studies reported a high inter-rater reliability for the assessment of outcomes (Brownell 2002 inter-rater reliability 0.86 to 0.94; Buday 1995: agreement rate 98%; Farmer 2003: agreement rate 91%). Loss to follow-up: No dropouts were reported in any of the studies. Performance bias: Medication levels were not monitored in the included studies. However, due to the short duration of the studies, it appears un- likely to assume significant differential change in medication might distort the results. All participants received the full therapy intervention as intended. Data reporting and analysis: One study (Buday 1995) reported means, standard deviations, and F test results for the outcomes described above. From these statistics it was possible to calculate an SMD with a standard error as appropriate for crossover studies… For the other two studies, individual patient data were extracted from tables or graphs. We screened the data for skewness before data synthesis. The data of one study (Farmer 2003) showed a skewed distribution. A log transformation would have removed the skewness, but would also have increased the effect size estimate. Therefore we decided to use the more conservative original scale. |
| Hennegan [442] | Cochrane collaboration risk of bias tool [433]; EPOC resources for review authors [443] | Three studies were non-randomised, representing a high risk of selection bias. Positively, these studies assessed and reported good comparability of intervention and control group characteristics at baseline. Wilson and colleagues reported that all outcomes were comparable at baseline. The primary outcome of school attendance was balanced at baseline in the study by Montgomery and colleagues, however, psychosocial outcomes were not balanced, representing a high risk for these outcomes. Of randomised studies, only one adequately reported methods of randomisation and allocation concealment. Of the three individually allocated studies, two were vulnerable to contamination. Education provided to girls in the same university dormitories or school could have easily been passed to friends in the control condition. All studies had a high risk of bias due to blinding, as it would not be possible to conceal the intervention from those providing or receiving education or sanitary products. Similarly, the majority of outcomes were self-reported by participants and most included items (e.g., attitudes) vulnerable to self-report biases, and may have been influenced by the lack of blinding. Two studies were considered to have a low risk of detection bias as they used official school attendance records. Whilst attendance data may not be highly reliable in the context of LMICs, both studies reported triangulating school records with researcher visits or girls’ own attendance diaries and noted a high level of attendance record reliability. It should be noted that for attitude items reported in Dolan et al. a high risk of detection bias is present. Mbvizo and colleagues tested menstrual knowledge by assessing students’ answers to questions about menstruation; judged to represent low risk of bias as the ability to answer accurately was unlikely to be altered by lack of blinding. There was considerable attrition in Wilson et al. as authors were unable to follow-up one intervention school. Djalalinia and colleagues stated in text that 5% of the sample was lost to follow-up, however reported N’s deviate from this figure with 1231 participants of 1823 (67.5%) reported on at two-years post intervention, with even smaller numbers displayed in results tables. Abedian et al. reported modest attrition, but failed to employ an intention-to-treat analysis. Fetohy and colleagues assessed outcomes immediately after the education session, resulting in no attrition. Despite a 15-month duration, Oster and colleagues reported negligible drop out (one participant), and employed an intention-to-treat analysis. Some missing outcome data was reported by Montgomery et al. but this was judged to be low risk in light of analytic approaches, including imputing missing values. Only one study reported trial registration. Of the remaining studies, five reported all outcomes included in methods. Fakhri and colleagues reported only an aggregated outcome. No other sources of bias were identified, although the suitability of outcome measures and study quality varied and are highlighted further in discussion. |
| Higgins [444] | Assessment made, but tool not specified | “Of the five included studies, two provided some data describing the number of participants who had dropped out of the study or were unavailable for follow-up outcome data collection (Barrett 2006; Lowry 2001, 2003). Both studies reported high attrition rate at both post-intervention and follow up. These studies are at a high risk of bias for incomplete outcome data (Barrett 2006). Lowry 2001, 2003 did not link attrition data to age, gender, intervention status, or severity of anxiety symptomology. Barrett 2006 did however link attrition data to intervention status and indicated that control groups were less likely to provide additional opportunities to screen children who were missing at assessment times. Attrition bias could not be assessed for the other three studies as they did not provide a description of withdrawals and drop-outs.” |
| Kavanagh [445] | Standardized framework from EPPI-Centre [446] | “Fifteen of the RCTs were judged sound for assessing effectiveness. Two were judged to be sound despite discrepancies with quality criteria (Lamb et al 1998; Lock and Barrett 2003). Lamb et al (1998) reported pre- and post-intervention data only for those remaining in the study, however attrition levels were low and the authors reported no difference between those that remained in the study and those who dropped out. Long-term follow up data from Lock and Barrett (2003) were excluded due to high levels of unequal attrition between control and intervention groups. Six studies reported methods of randomisation (Gillham et al 2006, 2007; Listug-Lunde 2005; Merry et al 2004a; Puskar et al 2003; Sheffield etal2006; Stein et al 2003). Two studies reported concealed allocation, and blinding of participants (Merry et al 2004a; Sheffield et al 2006). Five used cluster-randomisation of groups of participants (Lock and Barrett 2003; Poessel et al 2008; Ruini et al 2006; Sheffield et al 2006; Spence et al 2003)." |
| Marinho [447] | Cochrane collaboration risk of bias tool [433] | “We considered none of the included studies to be at low risk of bias overall. We considered eight studies to be at unclear risk of bias (Hagan 1985; Mainwaring 1978; Marthaler 1970; Olivier 1992; Ran 1991; Shern 1976; Truin 2005; Van Rijkom 2004). We considered the remaining 20 studies to be at high risk of bias.” |
| McDonald [448] | The Checklist for Measuring Quality [449] | “The scores for each study varied with a range between 16-26 out of a possible 32… Quality of studies was assessed to be between the 3rd and 5th quintile of possible scores on the Downs and Black Checklist.”  “Rosal 1993 scored the highest at 26 out of 32 which is in the 5th Quintile. This is due to the study design being assessed as reasonably robust but lacking adequate reporting on elements such as potential confounding factors. Regev 2005 scored 24 out of 32 which is in the 4th Quintile. This is due to the study design being assessed as reasonably robust but lacking adequate reporting on methods such as the analysis of the data. Khadar 2013a and 2013b appears to have been reported upon in two papers using the same format to report on the findings in relation to two different outcomes. There is a notable lack of reporting on the remaining 9 out of the 13 emotional and behavioural disorders which the measure used is intended to assess. It is not reported if any of the outcomes showed no change or scored worse at the end of the study than at the beginning. The authors of this study were emailed several times throughout the duration of this systematic review but no reply was received. The description of the methods used to operationalise this study is very sparse, leaving unanswered questions about the ways in which the data was collected, analysed and reported. For these reasons, both Khadar papers scored 16 out of 32 for quality which is in the 3rd Quintile." |
| Neil [450] | The Jadad Scale [451] | “The quality of the studies reviewed was quite poor, with only three of the 27 (11%) trials receiving a rating of three. The results of this review should therefore be interpreted with this in mind, although it is unlikely that the poor quality ratings impacted results. The low quality scores often resulted from an inability to achieve double-blind conditions and a failure to report the details of the randomization process or withdrawals and drop-outs. With the poor quality ratings often linked to inadequate trial reports, rather than poor trial designs, the results of the current studies can still be interpreted with confidence.” |
| Paul-Ebhohimhen [452] | Assessment made, but tool not specified | Not stated |
| Schroeder [453] | The Checklist for Measuring Quality [449] | “Regarding study reporting, all studies reported clear study objectives and outcomes of interest, though only one reported adverse events such as the child feeling stigmatized by participating in the intervention. Only 2 studies reported characteristics of patients lost to follow-up. Three studies reported attrition rates, with rates ranging from no attrition at 6 months to 21.2% attrition at 24 months. Concerning external validity, no study addressed whether the baseline sample was representative of the recruited population. Regarding internal validity, most studies did not report blinding of participants (except one which included an attention control) or outcomes assessors. Regarding confounding, although each quasi-experimental study provided a partial list of cofounders to be considered in group comparisons, statistical adjustment for confounders was incomplete. No study reported their method of allocation concealment. Only 3 studies reported a priori power analyses. Quality scores ranged between 12 and 19 points.” |
| Stein [359] | Cochrane collaboration risk of bias tool [433] | Summary of the risk of bias is displayed in Figure 2. No discussion of the results is included beyond the figure. |
| Sullivan [454] | Not stated | Not stated |
| Walter [455] | JBI-MAStARI [455] | “All six were determined to be of adequate quality after assessment using the JBI-MAStARI critical appraisal tool... All the articles met the minimum of six out of 10 questions... None of the included studies blinded participants to treatment allocation. None of the studies indicated that the allocation to treatment groups were concealed from the allocator, which increased the risk of selection bias. Risk of attrition bias was present in two of the studies. Risk of recall bias was present in two studies. None of the included studies blinded the assessors of outcomes to the treatment allocation of the groups, increasing the risk of detection bias.” |
| Werner-Seidler [456] | Cochrane collaboration risk of bias tool [433] | “There was evidence of selection bias, with only twenty-two studies (27%) reported that the allocation sequence had been adequately generated. This means that 73% of studies either did not provide sufficient detail to evaluate how groups were randomised (54 studies), or did not use a randomisation procedure that ensured comparability between groups (e.g., did not use a random number table; five studies). Similarly, 29 studies (36%) indicated adequate concealment of allocation, suggesting that a majority of studies did not report enough information to ascertain whether intervention allocations could have been foreseen prior to, or during enrolment. Risk of attrition bias was lower, with 32 studies indicating low risk of bias for addressing incomplete outcome data (40%), which indicates that the proportion of missing data was comparable across the study conditions. Bias from selective reporting was difficult to judge, with 5 studies clearly indicated low risk of bias (6%). Most studies (85%) did not report enough information in order to rule selective reporting out, which is largely contingent on publication of a study protocol to ensure authors report on outcomes they have previously indicated they will. 36% of studies had adequate protection against contamination by randomising at school level. This means that there was potential for contamination between conditions in 64% of studies, where participants in the control condition may have had access to material covered in the prevention condition, through contact with participants. Only 1 study was classified as having a low risk of bias for all five indices.” |

JBI-MAStARI = The Joanna Briggs Institute Meta-Analysis of Statistics Assessment and Review Instrument
