# Supplementary material for: Investigating the effectiveness of school health services delivered by a health provider: A systematic review of systematic reviews
Source: PLoS One. 2019 Jun 12;14(6):e0212603. doi: 10.1371/journal.pone.0212603 (PMC6561551; doi:10.1371/journal.pone.0212603)
Supplement: S5 Appendix — Table C. Calculation of corrected covered areas (CCAs). Table D. Classification of primary studies within included systematic reviews for use in corrected covered area calculation. (DOCX) [file pone.0212603.s005.docx]

**S5 APPENDIX. Corrected covered area**

$$\boldsymbol{CCA=}\left( \boldsymbol{N-r} \right)\boldsymbol{/(rc-r)}$$

Where:

N = total number of publications

r = number of index publications

c = number of reviews

CCA = corrected covered area

**Calculation of corrected covered areas (CCA) (Table C)**

| **Category** | **N** | **r** | **c** | **CCA %** |
| --- | --- | --- | --- | --- |
| Overall | 270 | 225 | 20 | 1 |
| Asthma (all SRs) | 15 | 15 | 2 | 0 |
| Menstrual management (all SRs) | 8 | 8 | 1 | 0 |
| Mental health (all SRs) | 160 | 117 | 9 | 5 |
| Mental health (SRs on mood disorders only) | 138 | 95 | 5 | 11 |
| Obesity (all SRs) | 11 | 11 | 1 | 0 |
| Oral health (all SRs) | 50 | 48 | 4 | 1 |
| Sexual and reproductive health (all SRs) | 12 | 12 | 1 | 0 |
| Sleep (all SRs) | 7 | 7 | 1 | 0 |
| Vision (all SRs) | 7 | 7 | 1 | 0 |

N = total number of publications; r = number of index publications; c = number of reviews; CCA = corrected covered area; SR = systematic review

**Classification of primary studies within included systematic reviews for use in corrected covered area calculation (Table D)**

| **Primary study ID**^a^ | **Systematic review ID**^b^ | **Classification for use in CCA calculation** |
| --- | --- | --- |
| Abadia 1978 | Marinho 2015 | Index |
| Abedian 2011 | Hennegan 2016 | Index |
| Agha 2002 | Paul Ebhohimhen 2008 | Index |
| Agha 2004 | Paul Ebhohimhen 2008 | Index |
| Al-Jundi 2006 | Stein 2017 | Index |
| Anticich 2013 | Werner-Seidler 2017 | Index |
| Anttonen 2011 | Stein 2017 | Index |
| Araya 2013 | Werner-Seidler 2017 | Index |
| Arnarson 2009 | Werner-Seidler 2017 | Index |
| Aune 2009 | Werner-Seidler 2017 | Index |
| Baker 2007 | Sullivan 2016 | Index |
| Balle 2010 | Werner-Seidler 2017 | Index |
| Barrett 2001 | Higgins 2015 | Index |
| Barrett 2001 | Neil 2009 | ***** |
| Barrett 2001 | Werner-Seidler 2017 | ***** |
| Barrett 2005 | Neil 2009 | Index |
| Barrett 2005 | Werner-Seidler 2017 | ***** |
| Barrett 2006 | Neil 2009 | Index |
| Beijamini 2012 | Chung 2017 | Index |
| Berger 2007 | Neil 2009 | Index |
| Berger 2007 | Werner-Seidler 2017 | ***** |
| Bijella 1981 | Marinho 2015 | Index |
| Bonhauser 2005 | Neil 2009 | Index |
| Bonnar 2015 | Chung 2017 | Index |
| Bonsergent 2012 | Schroeder 2016 | Index |
| Bouchard 2013 | Werner-Seidler 2017 | Index |
| Brownell 2002 | Gold 2006 | Index |
| Bruzzese 2008 | Walter 2016 | Index |
| Bryan 1970 | Marinho 2015 | Index |
| Buday 1995 | Gold 2006 | Index |
| Burden 1994 | Arora 2017 | Index |
| Butz 2005 | Walter 2016 | Index |
| Cain 2011 | Chung 2017 | Index |
| Calear 2009 | Werner-Seidler 2017 | Index |
| Cardemil 2002 (a) | Werner-Seidler 2017 | Index |
| Cardemil 2002 (b) | Werner-Seidler 2017 | Index |
| Castellanos 2006 | Kavanagh 2009 | Index |
| Castellanos 2006 | Neil 2009 | ***** |
| Castellanos 2006 | Werner-Seidler 2017 | ***** |
| Chandrashekar 2014 | Stein 2017 | Index |
| Chaplin 2006 | Kavanagh 2009 | Index |
| Chaplin 2006 | Bastounis 2016 | ***** |
| Chaplin 2006 | Werner-Seidler 2017 | ***** |
| Christiansen 1997 | Geryk 2017 | Index |
| Cicutto 2013 | Geryk 2017 | Index |
| Clark 2004 | Walter 2016 | Index |
| Clarke 1993 (a) | Werner-Seidler 2017 | Index |
| Clarke 1993 (b) | Werner-Seidler 2017 | Index |
| Clarke 1995 | Werner-Seidler 2017 | Index |
| Cobb 1980 | Marinho 2015 | Index |
| Congdon 2011 | Evans 2018 | Index |
| Cons 1970 | Marinho 2015 | Index |
| Cooley 2011 | Werner-Seidler 2017 | Index |
| Crane 2015 | Geryk 2017 | Index |
| Cunningham 2009 | Arora 2017 | Index |
| D'Cruz 2013 | Stein 2017 | Index |
| Dadds 1997 | Neil 2009 | Index |
| Dadds 1997 | Werner-Seidler 2017 | ***** |
| de Farias 2009 | Stein 2017 | Index |
| DePaola 1980 | Marinho 2015 | Index |
| Djalalinia | Hennegan 2016 | Index |
| Dura 2012 | Sullivan 2016 | Index |
| Ehntholt 2005 | Sullivan 2016 | Index |
| Ellis 2013 | Sullivan 2016 | Index |
| Englander 1967 | Marinho 2015 | Index |
| Englander 1971 | Marinho 2015 | Index |
| Englander 1978 | Marinho 2015 | Index |
| Essau 2000 | Higgins 2015 | Index |
| Essau 2012 | Werner-Seidler 2017 | Index |
| Esteves 1996 | Stein 2017 | Index |
| Fakhri 2012 | Hennegan 2016 | Index |
| Farmer 2003 | Gold 2006 | Index |
| Fawole 1999 | Paul Ebhohimhen 2008 | Index |
| Fazel 2009 | Sullivan 2016 | Index |
| Fetohy 2007 | Hennegan 2016 | Index |
| Fitzgerald 1999 | Paul Ebhohimhen 2008 | Index |
| Fox 2005 | Sullivan 2016 | Index |
| Garaigordobil 2004 | Neil 2009 | Index |
| Garaigordobil 2004 | Werner-Seidler 2017 | ***** |
| Gilham 2007 | Bastounis 2016 | ***** |
| Gillham 2006 | Kavanagh 2009 | Index |
| Gillham 2006 | Neil 2009 | ***** |
| Gillham 2006 | Werner-Seidler 2017 | ***** |
| Gillham 2007 | Kavanagh 2009 | Index |
| Gillham 2007 | Werner-Seidler 2017 | ***** |
| Gillham 2012 | Werner-Seidler 2017 | Index |
| Ginsburg 2002 | Neil 2009 | Index |
| Gisselsson 1999 | Marinho 2015 | Index |
| Gregory 2000 | Geryk 2017 | Index |
| Hagan 1985 | Marinho 2015 | Index |
| Hains 1990 | Neil 2009 | Index |
| Hains 1990 | Werner-Seidler 2017 | ***** |
| Hains 1992 | Neil 2009 | Index |
| Hains 1992 | Werner-Seidler 2017 | ***** |
| Hains 1994 | Neil 2009 | Index |
| Hains 1994 | Werner-Seidler 2017 | ***** |
| Haleem 2012 | Stein 2017 | Index |
| Harvey 2000 | Paul Ebhohimhen 2008 | Index |
| Hawthorne 2011 | Schroeder 2016 | Index |
| Hebbal 2005 | Arora 2017 | Index |
| Heifetz 1970 | Marinho 2015 | Index |
| Hiebert 1989 | Neil 2009 | Index |
| Hiebert 1989 | Werner-Seidler 2017 | Index |
| Horner 2008 | Geryk 2017 | Index |
| Horner 2014 | Geryk 2017 | Index |
| Horowitz 1971 | Marinho 2015 | Index |
| Horowitz 1974 | Marinho 2015 | Index |
| Horowitz 2007 | Werner-Seidler 2017 | Index |
| Howell 2006 | Geryk 2017 | Index |
| Hunt 2009 | Werner-Seidler 2017 | Index |
| Ingraham 1970 | Marinho 2015 | Index |
| Ivanovic 1996 | Stein 2017 | Index |
| James 2005 | Paul Ebhohimhen 2008 | Index |
| Jiang 2005 | Marinho 2015 | Index |
| Johnston 2013 | Schroeder 2016 | Index |
| Johnstone 2014 | Werner-Seidler 2017 | Index |
| Jordans 2010 | Werner-Seidler 2017 | Index |
| Kalantari 2012 | Sullivan 2016 | Index |
| Keogh 2006 | Neil 2009 | Index |
| Keogh 2006 | Werner-Seidler 2017 | ***** |
| Khadar 2013a | McDonald 2018 | Index |
| Khadar 2013b | McDonald 2018 | Index |
| Kindt 2014 | Bastounis 2016 | Index |
| Kindt 2014 | Werner-Seidler 2017 | ***** |
| King 1990 | Werner-Seidler 2017 | Index |
| Kira 2014 | Chung 2017 | Index |
| Kiselica 1994 | Neil 2009 | Index |
| Kiselica 1994 | Werner-Seidler 2017 | ***** |
| Klepp 1994 | Paul Ebhohimhen 2008 | Index |
| Klepp 1997 | Paul Ebhohimhen 2008 | Index |
| Kraag 2009 | Werner-Seidler 2017 | Index |
| Kuhn 1994 | Paul Ebhohimhen 2008 | Index |
| Lamb 1998 | Kavanagh 2009 | Index |
| Lamb 1998 | Werner-Seidler 2017 | ***** |
| Listug 2005 | Kavanagh 2009 | Index |
| Lock 2003 | Kavanagh 2009 | Index |
| Lock 2003 | Higgins 2015 | ***** |
| Lock 2003 | Neil 2009 | ***** |
| Lock 2003 | Werner-Seidler 2017 | ***** |
| Lowry 2001 | Higgins 2015 | Index |
| Lowry 2001 | Neil 2009 | ***** |
| Lowry 2001 | Werner-Seidler 2017 | ***** |
| Mainwaring 1978 | Marinho 2015 | Index |
| Malgady 1990 | Neil 2009 | Index |
| Manassis 2010 | Werner-Seidler 2017 | Index |
| Marthaler 1970 | Marinho 2015 | Index |
| Marthaler 1970a | Marinho 2015 | Index |
| Masia 2005 | Kavanagh 2009 | Index |
| Masia 2005 | Neil 2009 | ***** |
| Masia 2007 | Kavanagh 2009 | Index |
| Mbizvo 1997 | Hennegan 2016 | Index |
| McCarty 2010 | Werner-Seidler 2017 | Index |
| McCarty 2013 | Werner-Seidler 2017 | Index |
| McGhan 2003 | Walter 2016 | Index |
| McGhan 2010 | Walter 2016 | Index |
| McLoone 2012 | Werner-Seidler 2017 | Index |
| Melin 2009 | Schroeder 2016 | Index |
| Meng 2000 | Geryk 2017 | Index |
| Merry 2004 | Kavanagh 2009 | Index |
| Merry 2004 | Werner-Seidler 2017 | ***** |
| Mestrinho 1983 | Marinho 2015 | Index |
| Mifsud 2005 | Werner-Seidler 2017 | Index |
| Miller 2010 | Werner-Seidler 2017 | Index |
| Miller 2011a | Werner-Seidler 2017 | Index |
| Miller 2011b | Werner-Seidler 2017 | Index |
| Miller 2011b (2) | Werner-Seidler 2017 | Index |
| Milsom 2006 | Arora 2017 | Index |
| Misfud 2005 | Neil 2009 | Index |
| Mitchum 1999 | Brendel 2014 | Index |
| Montgomery 2012 | Hennegan 2016 | Index |
| Morjaria 2016 | Evans 2018 | Index |
| Moseley 2009 | Chung 2017 | Index |
| Munodawafa 1995 | Paul Ebhohimhen 2008 | Index |
| Nobel 2012 | Werner-Seidler 2017 | Index |
| O'Shea 2000 | Sullivan 2016 | Index |
| Olivier 1992 | Marinho 2015 | Index |
| Oster 2010 | Hennegan 2016 | Index |
| Patterson 2005 | Geryk 2017 | Index |
| Pattison 2001 | Neil 2009 | Index |
| Pattison 2001 | Bastounis 2016 | ***** |
| Pattison 2001 | Werner-Seidler 2017 | ***** |
| Pbert 2012 | Schroeder 2016 | Index |
| Pedro 1985 | Werner-Seidler 2017 | Index |
| Petrecca 1994 | Cooper 2013 | Index |
| Poessel 2004 | Werner-Seidler 2017 | Index |
| Poessel 2008 | Kavanagh 2009 | Index |
| Poessel 2008 | Werner-Seidler 2017 | ***** |
| Poessel 2013 | Werner-Seidler 2017 | Index |
| Praveen 2014 | Arora 2017 | Index |
| Puskar 2003 | Kavanagh 2009 | Index |
| Puskar 2003 | Werner-Seidler 2017 | ***** |
| Quayle 2001 | Bastounis 2016 | Index |
| Quayle 2001 | Werner-Seidler 2017 | ***** |
| Raes 2014 | Werner-Seidler 2017 | Index |
| Ran 1991 | Marinho 2015 | Index |
| RECS 2009 | Evans 2018 | Index |
| Regev 2005 | McDonald 2018 | Index |
| Rigney 2015 | Chung 2017 | Index |
| Rivet 2011 | Werner-Seidler 2017 | Index |
| Robbins 2012 | Schroeder 2016 | Index |
| Roberts 2003 | Neil 2009 | Index |
| Roberts 2003 | Werner-Seidler 2017 | ***** |
| Roberts 2010 | Bastounis 2016 | Index |
| Roberts 2010 | Werner-Seidler 2017 | ***** |
| Rodgers 2015 | Higgins 2015 | Index |
| Rodrigues 2003 | Stein 2017 | Index |
| Rohde 2014 | Werner-Seidler 2017 | Index |
| Rooney 2006 | Neil 2009 | Index |
| Rooney 2006 | Bastounis 2016 | ***** |
| Rooney 2006 | Werner-Seidler 2017 | ***** |
| Rooney 2013 | Bastounis 2016 | Index |
| Rosal 1993 | McDonald 2018 | Index |
| Rose 2014 | Werner-Seidler 2017 | Index |
| Rousseau 2005 | Sullivan 2016 | Index |
| Rousseau 2007 | Sullivan 2016 | Index |
| Rousseau 2012 | Sullivan 2016 | Index |
| Ruini 2006 | Kavanagh 2009 | Index |
| Ruini 2006 | Werner-Seidler 2017 | ***** |
| Ruini 2009 | Werner-Seidler 2017 | Index |
| Rusakaniko 1997 | Paul Ebhohimhen 2008 | Index |
| Saied 2009 | Cooper 2013 | Index |
| Salisbury 2002 | Geryk 2017 | Index |
| Schottelkorb 2012 | Sullivan 2016 | Index |
| Sheffield 2006 | Kavanagh 2009 | Index |
| Sheffield 2006 | Neil 2009 | ***** |
| Sheffield 2006 | Werner-Seidler 2017 | ***** |
| Shern 1976 | Marinho 2015 | Index |
| SIL 2014 | Evans 2018 | Index |
| Sil II 2015 | Evans 2018 | Index |
| Siu 2007 | Werner-Seidler 2017 | Index |
| Spence 2003 | Kavanagh 2009 | Index |
| Spence 2003 | Werner-Seidler 2017 | ***** |
| Speroni 2007 | Schroeder 2016 | Index |
| Stallard 2012 | Werner-Seidler 2017 | Index |
| Stallard 2014 | Werner-Seidler 2017 | Index |
| Stanton 1998 | Paul Ebhohimhen 2008 | Index |
| Stein 2003 | Kavanagh 2009 | Index |
| Stice 2007 | Werner-Seidler 2017 | Index |
| Stice 2008 | Werner-Seidler 2017 | Index |
| Stolberg 1994 | Neil 2009 | Index |
| Szwejda 1972 | Marinho 2015 | Index |
| Tak 2015 | Bastounis 2016 | Index |
| Tol 2008 | Werner-Seidler 2017 | Index |
| Treie 1988 | Marinho 2015 | Index |
| Trubman 1973 | Marinho 2015 | Index |
| Truin 2005 | Marinho 2015 | Index |
| Tucker 2015 | Schroeder 2016 | Index |
| Van Rijkom 2004 | Marinho 2015 | Index |
| Walker 2008 | Walter 2016 | Index |
| WEAR 2017 | Evans 2018 | Index |
| Wedner 2008 | Evans 2018 | Index |
| Wijnhoven 2014 | Werner-Seidler 2017 | Index |
| Williams 2011 | Schroeder 2016 | Index |
| Wilson 2014 | Hennegan 2016 | Index |
| Wing 2015 | Chung 2017 | Index |
| Wong 2013 | Schroeder 2016 | Index |
| Wong 2014 | Werner-Seidler 2017 | Index |
| Woods 2011 | Werner-Seidler 2017 | Index |
| Worthington 2001 | Cooper 2013 | Index |
| Worthington 2001 | Stein 2017 | ***** |
| Wright 2013 | Schroeder 2016 | Index |
| Yankey 2012 | Sullivan 2016 | Index |
| Yekaninejad 2012 | Stein 2017 | Index |
| Young 2006 | Werner-Seidler 2017 | Index |
| Young 2010 | Werner-Seidler 2017 | Index |
| Yu 2002 | Kavanagh 2009 | Index |
| Yu 2002 | Werner-Seidler 2017 | ***** |
| Zanin 2007 | Cooper 2013 | Index |
| Zanin 2007 | Stein 2017 | ***** |
| Zarod 1992 | Arora 2017 | Index |

^a^ Identification for all primary studies within included systematic reviews

^b^ Identification for all included systematic reviews (SRs)

Index = first appearance of a publication; ***** = duplicate; CCA = corrected covered area
