# Supplementary material for: Investigating the effectiveness of school health services delivered by a health provider: A systematic review of systematic reviews
Source: PLoS One. 2019 Jun 12;14(6):e0212603. doi: 10.1371/journal.pone.0212603 (PMC6561551; doi:10.1371/journal.pone.0212603)
Supplement: S6 Appendix — Table E. AMSTAR 2 checklist with designations of critical and non-critical. Table F. Answers to AMSTAR 2 checklist questions 1–16. (DOCX) [file pone.0212603.s006.docx]

**S6 APPENDIX. AMSTAR 2 classifications and results**

**AMSTAR 2 checklist [457] with designations of critical (serious flaws if “no”) and non-critical (not serious weaknesses if “no”) (Table E)**

| **Question number** | **Question from AMSTAR 2 Checklist [457]** | **Decision^a^** |
| --- | --- | --- |
| 1 | Did the research questions and inclusion criteria for the review include the components of PICO? | C |
| 2 | Did the report of the review contain an explicit statement that the review methods were established prior to the conduct of the review and did the report justify any significant deviations from the protocol? | NC |
| 3 | Did the review authors explain their selection of the study designs for inclusion in the review? | NC |
| 4 | Did the review authors use a comprehensive literature search strategy? | C |
| 5 | Did the review authors perform study selection in duplicate? | C |
| 6 | Did the review authors perform data extraction in duplicate? | C |
| 7 | Did the review authors provide a list of excluded studies and justify the exclusions? | NC |
| 8 | Did the review authors describe the included studies in adequate detail? | C |
| 9 | Did the review authors use a satisfactory technique for assessing the RoB in individual studies that were included in the review?  9a. For reviews with RCTS  9b. For reviews with NRSI | C |
| 10 | Did the review authors report on the sources of funding for the studies included in the review? | NC |
| 11 | If meta-analysis was performed did the review authors use appropriate methods for statistical combination of results?  11a. For reviews with RCTs  11b. For reviews with NRSIs | C |
| 12 | If meta-analysis was performed, did the review authors assess the potential impact of RoB in individual studies on the results of the meta-analysis or other evidence synthesis? | NC |
| 13 | Did the review authors account for RoB in individual studies when interpreting/ discussing the results of the review? | C |
| 14 | Did the review authors provide a satisfactory explanation for, and discussion of, any heterogeneity observed in the results of the review? | C |
| 15 | If they performed quantitative synthesis did the review authors carry out an adequate investigation of publication bias (small study bias) and discuss its likely impact on the results of the review? | NC |
| 16 | Did the review authors report any potential sources of conflict of interest, including any funding they received for conducting the review? | NC |

^a^ = decision of whether each question is critical or non-critical for the purposes of this overview, determined through discussion between 2 reviewers until consensus was reached;

PICO = population, intervention, comparison, outcome; C = critical flaw if no; NC = non-critical weakness if no; RCT = randomized controlled trial; NRSI = non-randomized study of interventions; RoB = risk of bias

**Answers to AMSTAR 2 checklist questions 1-16^a^ (Table F)**

|  | **1** | **2** | **3** | **4** | **5** | **6** | **7** | **8** | **9a** | **9b** | **10** | **11a** | **11b** | **12** | **13** | **14** | **15** | **16** |
| --- | --- | --- | --- | --- | --- | --- | --- | --- | --- | --- | --- | --- | --- | --- | --- | --- | --- | --- |
| Arora [432] | + | - | - | +/- | + | + | + | + | + | ORCT | + | + | - | + | + | + | - | + |
| Bastounis [434] | + | - | + | - | + | + | - | +/- | + | ORCT | - | + | - | - | + | + | - | - |
| Brendel [436] | - | - | - | - | - | + | + | - | ONRSI | - | - | NMA | NMA | NMA | + | + | NMA | + |
| Chung [437] | - | - | - | - | + | + | - | +/- | + | ORCT | - | - | - | + | + | + | - | - |
| Cooper [438] | + | - | - | +/- | + | + | + | + | + | ORCT | + | NMA | NMA | NMA | + | + | NMA | + |
| Evans [439] | + | - | - | +/- | + | + | + | + | + | ORCT | + | + | - | + | + | + | - | + |
| Geryk [440] | - | - | - | - | + | + | - | - | - | - | - | NMA | NMA | NMA | - | + | NMA | - |
| Gold [441] | + | - | + | - | + | + | + | + | +/- | ORCT | - | + | - | + | - | + | - | + |
| Hennegan [442] | - | - | + | +/- | + | - | + | +/- | + | +/- | - | NMA | - | - | + | + | - | + |
| Higgins [444] | + | - | + | - | - | - | - | + | - | ORCT | - | NMA | NMA | NMA | - | - | NMA | - |
| Kavanagh [445] | + | - | - | - | - | - | - | + | +/- | ORCT | - | + | - | + | + | + | - | + |
| Marinho, 2015 [447] | + | - | + | +/- | + | + | + | + | + | ORCT | + | + | - | + | + | + | + | + |
| McDonald [448] | + | - | - | - | + | + | + | +/- | - | +/- | - | NMA | NMA | NMA | + | - | NMA | + |
| Neil [450] | - | - | - | - | - | + | - | + | +/- | ORCT | - | - | NMA | NMA | + | - | NMA | - |
| Paul-Ebhohimhen [452] | + | - | + | +/- | - | + | + | + | - | - | - | NMA | NMA | NMA | - | - | NMA | + |
| Schroeder [453] | + | - | - | - | - | - | - | + | +/- | +/- | - | + | - | - | + | + | + | - |
| Stein [359] | + | - | - | +/- | + | - | - | +/- | + | ORCT | - | + | - | - | - | + | - | - |
| Sullivan [454] | - | - | - | - | - | - | - | - | - | - | - | NMA | NMA | NMA | - | - | NMA | - |
| Walter [455] | + | - | - | - | - | - | + | +/- | +/- | ORCT | - | NMA | NMA | NMA | + | + | NMA | + |
| Werner-Seidler [456] | + | - | - | - | + | + | - | + | - | ORCT | - | + | - | - | + | + | + | - |

^a^ refer to Table S3 for questions within the AMSTAR 2 checklist

+ = yes; - = no; +/- = partial yes; ORCT = only randomized controlled trials included; ONRSI = only non-randomized study of interventions; NMA = no meta-analysis conducted
