# Supplementary material for: Investigating the effectiveness of school health services delivered by a health provider: A systematic review of systematic reviews
Source: PLoS One. 2019 Jun 12;14(6):e0212603. doi: 10.1371/journal.pone.0212603 (PMC6561551; doi:10.1371/journal.pone.0212603)
Supplement: S8 Appendix — (DOCX) [file pone.0212603.s008.docx]

**S8. APPENDIX REFERENCES**

1. Baltag V, Pachyna A, Hall J. Global Overview of School Health Services: Data from 102 Countries. Health Behav Policy Rev. 2015;2: 268–283. doi:10.14485/HBPR.2.4.4

2. Health for the World’s Adolescents: A second chance in the second decade [Internet]. Geneva: World Health Organization; 2014. Available: www.who.int/adolescent/second-decade

3. Ballard M, Montgomery P. Risk of bias in overviews of reviews: a scoping review of methodological guidance and four-item checklist. Res Synth Methods. 2017;8: 92–108. doi:10.1002/jrsm.1229

4. Pieper D, Antoine S-L, Mathes T, Neugebauer EAM, Eikermann M. Systematic review finds overlapping reviews were not mentioned in every other overview. J Clin Epidemiol. 2014;67: 368–375. doi:10.1016/j.jclinepi.2013.11.007

5. Shea BJ, Reeves BC, Wells G, Thuku M, Hamel C, Moran J, et al. AMSTAR 2: a critical appraisal tool for systematic reviews that include randomised or non-randomised studies of healthcare interventions, or both. BMJ. 2017;358: j4008. doi:10.1136/bmj.j4008

6. Langford R, Bonell CP, Jones HE, Pouliou T, Murphy SM, Waters E, et al. The WHO Health Promoting School framework for improving the health and well‐being of students and their academic achievement. The Cochrane Library. John Wiley & Sons, Ltd; 2014. Available: http://onlinelibrary.wiley.com/doi/10.1002/14651858.CD008958.pub2/full

7. Adair PM, Burnside G, Pine CM. Analysis of health behaviour change interventions for preventing dental caries delivered in primary schools. Caries Res. 2013;47 Suppl 1: 2–12. doi:10.1159/000351829

8. Adi Y, Killoran A, Janmohamed K, Stewart-Brown S. Systematic review of the effectiveness of interventions to promote mental wellbeing in children in primary education. Report 1: Universal approaches: non-violence related outcomes. PubMed Health. 2007; Available: https://www.ncbi.nlm.nih.gov/pubmedhealth/PMH0024581/

9. Adi Y, Schrader McMillan A, Kiloran A, Stewart-Brown S. Systematic review of the effectiveness of interventions to promote mental wellbeing in primary schools. Report 3: Universal Approaches with focus on prevention of violence and bullying. 2007 Sep.

10. Agabio R, Trincas G, Floris F, Mura G, Sancassiani F, Angermeyer MC. A Systematic Review of School-Based Alcohol and other Drug Prevention Programs. Clin Pract Epidemiol Ment Health CP EMH. 2015;11: 102–112. doi:10.2174/1745017901511010102

11. Ahmad E, Grimes DE. The effects of self-management education for school-age children on asthma morbidity: a systematic review. J Sch Nurs Off Publ Natl Assoc Sch Nurses. 2011;27: 282–292. doi:10.1177/1059840511403003

12. Akers AY, Holland CL, Bost J. Interventions to improve parental communication about sex: a systematic review. Pediatrics. 2011;127: 494–510. doi:10.1542/peds.2010-2194

13. Allen T, Parker M. Deworming delusions? Mass drug administration in east African schools. J Biosoc Sci. 2016;48 Suppl 1: S116–147. doi:10.1017/S0021932016000171

14. Aloia CR, Shockey TA, Nahar VK, Knight KB. Pertinence of the recent school-based nutrition interventions targeting fruit and vegetable consumption in the United States:a systematic review. Health Promot Perspect. 2016;6: 1–9. doi:10.15171/hpp.2016.01

15. Altaf F, Drinkwater J, Phan K, Cree AK. Systematic Review of School Scoliosis Screening. Spine Deform. 2017;5: 303–309. doi:10.1016/j.jspd.2017.03.009

16. Amaugo LG, Papadopoulos C, Ochieng BMN, Ali N. The effectiveness of HIV/AIDS school-based sexual health education programmes in Nigeria: a systematic review. Health Educ Res. 2014;29: 633–648. doi:10.1093/her/cyu002

17. Anderson JE, Lowen CA. Connecting youth with health services: Systematic review. Can Fam Physician Med Fam Can. 2010;56: 778–784.

18. Arbesman M, Bazyk S, Nochajski SM. Systematic review of occupational therapy and mental health promotion, prevention, and intervention for children and youth. Am J Occup Ther Off Publ Am Occup Ther Assoc. 2013;67: e120–130. doi:10.5014/ajot.2013.008359

19. Atilola O, Ola B. Towards school mental health programmes in Nigeria: systematic review revealed the need for contextualised and culturally-nuanced research. J Child Adolesc Ment Health. 2016;28: 47–70. doi:10.2989/17280583.2016.1144607

20. Atkin AJ, Gorely T, Biddle SJH, Cavill N, Foster C. Interventions to Promote Physical Activity in Young People Conducted in the Hours Immediately After School: A Systematic Review. Int J Behav Med. 2011;18: 176–187. doi:10.1007/s12529-010-9111-z

21. Aveyard P, Olekan U, Yahaya I, Pennant M, Bayliss S, Jit M, et al. School-based interventions to prevent the uptake of smoking among children and young people; effectiveness review. 2009; Available: https://www.phc.ox.ac.uk/publications/365353

22. Bains RM, Diallo AF. Mental Health Services in School-Based Health Centers: Systematic Review. J Sch Nurs. 2016;32: 8–19. doi:10.1177/1059840515590607

23. Bamford J, Fortnum H, Bristow K, Smith J, Vamvakas G, Davies L, et al. Current practice, accuracy, effectiveness and cost-effectiveness of the school entry hearing screen. Health Technol Assess. 2007;11: 1–+.

24. Barlow J, Stewart-Brown S, Fletcher J. Systematic review of the school entry medical examination. Arch Dis Child. 1998;78: 301–311.

25. Barr-Anderson DJ, AuYoung M, Whitt-Glover MC, Glenn BA, Yancey AK. Integration of short bouts of physical activity into organizational routine a systematic review of the literature. Am J Prev Med. 2011;40: 76–93. doi:10.1016/j.amepre.2010.09.033

26. Barry MM, Clarke AM, Jenkins R, Patel V. A systematic review of the effectiveness of mental health promotion interventions for young people in low and middle income countries. Bmc Public Health. 2013;13: 835. doi:10.1186/1471-2458-13-835

27. Bayer J, Hiscock H, Scalzo K, Mathers M, McDonald M, Morris A, et al. Systematic review of preventive interventions for children’s mental health: what would work in Australian contexts? Aust N Z J Psychiatry. 2009;43: 695–710. doi:10.1080/00048670903001893

28. Beets MW, Beighle A, Erwin HE, Huberty JL. After-school program impact on physical activity and fitness: a meta-analysis. Am J Prev Med. 2009;36: 527–537. doi:10.1016/j.amepre.2009.01.033

29. Bellamy R. A systematic review of educational interventions for promoting sun protection knowledge, attitudes and behaviour following the QUESTS approach. Med Teach. 2005;27: 269–275. doi:10.1080/01421590400029558

30. Bennett K, Manassis K, Duda S, Bagnell A, Bernstein GA, Garland EJ, et al. PREVENTING CHILD AND ADOLESCENT ANXIETY DISORDERS: OVERVIEW OF SYSTEMATIC REVIEWS. Depress Anxiety. 2015;32: 909–918. doi:10.1002/da.22400

31. Bennett SE, Assefi NP. School-based teenage pregnancy prevention programs: a systematic review of randomized controlled trials. J Adolesc Health. 2005;36: 72–81. doi:10.1016/j.jadohealth.2003.11.097

32. Berkowitz M, Bier M. What Works In Character Education. J Res Character Educ. 2007;5.

33. Bird Y, Obidiya O, Mahmood R, Nwankwo C, Moraros J. Human Papillomavirus Vaccination Uptake in Canada: A Systematic Review and Meta-analysis. Int J Prev Med. 2017;8: 71. doi:10.4103/ijpvm.IJPVM_49_17

34. Birdee GS, Yeh GY, Wayne PM, Phillips RS, Davis RB, Gardiner P. Clinical applications of yoga for the pediatric population: a systematic review. Acad Pediatr. 2009;9: 212–220.e1–9. doi:10.1016/j.acap.2009.04.002

35. Blank L, Baxter SK, Payne N, Guillaume LR, Pilgrim H. Systematic review and narrative synthesis of the effectiveness of contraceptive service interventions for young people, delivered in educational settings. J Pediatr Adolesc Gynecol. 2010;23: 341–351. doi:10.1016/j.jpag.2010.03.007

36. Blank L, Baxter S, Goyder E, Guillaume L, Wilkinson A, Hummel S, et al. Systematic review of the effectiveness of universal interventions which aim to promote emotional and social wellbeing in secondary schools [Internet]. Sheffield: School of Health and Related Research (ScHARR); 2009. Available: https://www.sheffield.ac.uk/polopoly_fs/1.44221!/file/Wellbeing-in-schools.pdf

37. Blank L, Baxter S, Goyder E, Naylor P, Guillaume L, Wilkinson A, et al. Promoting well‐being by changing behaviour: a systematic review and narrative synthesis of the effectiveness of whole secondary school behavioural interventions. Ment Health Rev J. 2010;15: 43–53. doi:10.5042/mhrj.2010.0371

38. Borde R, Smith JJ, Sutherland R, Nathan N, Lubans DR. Methodological considerations and impact of school-based interventions on objectively measured physical activity in adolescents: a systematic review and meta-analysis. Obes Rev. 2017;18: 476–490. doi:10.1111/obr.12517

39. Bothe AK, Davidow JH, Bramlett RE, Ingham RJ. Stuttering treatment research 1970-2005: I. Systematic review incorporating trial quality assessment of behavioral, cognitive, and related approaches. Am J Speech Lang Pathol. 2006;15: 321–341. doi:10.1044/1058-0360(2006/031)

40. Boyce MR, O’Meara WP. Use of malaria RDTs in various health contexts across sub-Saharan Africa: a systematic review. BMC Public Health. 2017;17: 470. doi:10.1186/s12889-017-4398-1

41. Brackney DE, Cutshall M. Prevention of Type 2 Diabetes among Youth: A Systematic Review, Implications for the School Nurse. J Sch Nurs. 2015;31: 6–21. doi:10.1177/1059840514535445

42. Brennan JH, Mitra B, Synnot A, McKenzie J, Willmott C, McIntosh AS, et al. Accelerometers for the Assessment of Concussion in Male Athletes: A Systematic Review and Meta-Analysis. Sports Med. 2017;47: 469–478. doi:10.1007/s40279-016-0582-1

43. Bröning S, Kumpfer K, Kruse K, Sack P-M, Schaunig-Busch I, Ruths S, et al. Selective prevention programs for children from substance-affected families: a comprehensive systematic review. Subst Abuse Treat Prev Policy. 2012;7: 23. doi:10.1186/1747-597X-7-23

44. Brown T, Platt S, Amos A. Equity impact of interventions and policies to reduce smoking in youth: systematic review. Tob Control. 2014;23: e98–e105. doi:10.1136/tobaccocontrol-2013-051451

45. Brown T, Summerbell C. Systematic review of school-based interventions that focus on changing dietary intake and physical activity levels to prevent childhood obesity: an update to the obesity guidance produced by the National Institute for Health and Clinical Excellence. Obes Rev. 2009;10: 110–141. doi:10.1111/j.1467-789X.2008.00515.x

46. Burnim M, Ivy JA, King CH. Systematic review of community-based, school-based, and combined delivery modes for reaching school-aged children in mass drug administration programs for schistosomiasis. PLoS Negl Trop Dis. 2017;11: e0006043. doi:10.1371/journal.pntd.0006043

47. Cai L, Wu Y, Cheskin LJ, Wilson RF, Wang Y. The Effect of Childhood Obesity Prevention Programs on Blood Lipids: A Systematic Review and Meta-analysis. Obes Rev Off J Int Assoc Study Obes. 2014;15: 933–944. doi:10.1111/obr.12227

48. Calear AL, Christensen H. Systematic review of school-based prevention and early intervention programs for depression. J Adolesc. 2010;33: 429–438. doi:10.1016/j.adolescence.2009.07.004

49. Camacho-Miñano MJ, LaVoi NM, Barr-Anderson DJ. Interventions to promote physical activity among young and adolescent girls: a systematic review. Health Educ Res. 2011;26: 1025–1049. doi:10.1093/her/cyr040

50. Campbell M, Buckeridge D, Dwyer J, Fong S, Mann V, Sanchez-Sweatman O, et al. A systematic review of the effectiveness of environmental awareness interventions. Can J Public Health Rev Can Sante Publique. 2000;91: 137–143.

51. Canter KS, Roberts MC. A Systematic and Quantitative Review of Interventions to Facilitate School Reentry for Children With Chronic Health Conditions. J Pediatr Psychol. 2012;37: 1065–1075. doi:10.1093/jpepsy/jss071

52. Cardoza VJ, Documét PI, Fryer CS, Gold MA, Butler J. Sexual health behavior interventions for U.S. Latino adolescents: a systematic review of the literature. J Pediatr Adolesc Gynecol. 2012;25: 136–149. doi:10.1016/j.jpag.2011.09.011

53. Carlin A, Murphy MH, Gallagher AM. Do Interventions to Increase Walking Work? A Systematic Review of Interventions in Children and Adolescents. Sports Med Auckl Nz. 2016;46: 515–530. doi:10.1007/s40279-015-0432-6

54. Carney T, Myers BJ, Louw J, Okwundu CI. Brief school‐based interventions and behavioural outcomes for substance‐using adolescents. The Cochrane Library. John Wiley & Sons, Ltd; 2016. Available: http://onlinelibrary.wiley.com/doi/10.1002/14651858.CD008969.pub3/abstract

55. Carr AB, Ebbert JO. Interventions for tobacco cessation in the dental setting. A systematic review. Community Dent Health. 2007;24: 70–74.

56. Carr AB, Ebbert J. Interventions for tobacco cessation in the dental setting. The Cochrane Library. John Wiley & Sons, Ltd; 2012. Available: http://onlinelibrary.wiley.com/doi/10.1002/14651858.CD005084.pub3/abstract

57. Carroll C, Lloyd-Jones M, Cooke J, Owen J. Reasons for the use and non-use of school sexual health services: a systematic review of young people’s views. J Public Health. 2012;34: 403–410. doi:10.1093/pubmed/fdr103

58. Carson KV, Brinn MP, Labiszewski NA, Esterman AJ, Chang AB, Smith BJ. Community interventions for preventing smoking in young people. Cochrane Database of Systematic Reviews. John Wiley & Sons, Ltd; 2011. Available: http://onlinelibrary.wiley.com/doi/10.1002/14651858.CD001291.pub2/abstract

59. Cawley J, Hull HF, Rousculp MD. Strategies for Implementing School-Located Influenza Vaccination of Children: A Systematic Literature Review. J Sch Health. 2010;80: 167–175.

60. Chalamandaris A-G, Piette D. School-based anti-bullying interventions: Systematic review of the methodology to assess their effectiveness. Aggress Violent Behav. 2015;24: 131–174. doi:10.1016/j.avb.2015.04.004

61. Charania MR, Crepaz N, Guenther-Gray C, Henny K, Liau A, Willis LA, et al. Efficacy of Structural-Level Condom Distribution Interventions: A Meta-Analysis of U.S. and International Studies, 1998–2007. AIDS Behav. 2011;15: 1283–1297. doi:10.1007/s10461-010-9812-y

62. Cheney G, Schlösser A, Nash P, Glover L. Targeted group-based interventions in schools to promote emotional well-being: a systematic review. Clin Child Psychol Psychiatry. 2014;19: 412–438. doi:10.1177/1359104513489565

63. Chin HB, Sipe TA, Elder R, Mercer SL, Chattopadhyay SK, Jacob V, et al. The effectiveness of group-based comprehensive risk-reduction and abstinence education interventions to prevent or reduce the risk of adolescent pregnancy, human immunodeficiency virus, and sexually transmitted infections: two systematic reviews for the Guide to Community Preventive Services. Am J Prev Med. 2012;42: 272–294. doi:10.1016/j.amepre.2011.11.006

64. Chong LY, Clarkson JE, Dobbyn‐Ross L, Bhakta S. Slow‐release fluoride devices for the control of dental decay. The Cochrane Library. John Wiley & Sons, Ltd; 2014. Available: http://onlinelibrary.wiley.com/doi/10.1002/14651858.CD005101.pub3/abstract

65. Chong L-Y, Clarkson JE, Dobbyn‐Ross L, Bhakta S. Slow‐release fluoride devices for the control of dental decay. The Cochrane Library. John Wiley & Sons, Ltd; 2018. Available: http://cochranelibrary-wiley.com/doi/10.1002/14651858.CD005101.pub4/full

66. Clemmens D, Hayman LL. Increasing activity to reduce obesity in adolescent girls: a research review. J Obstet Gynecol Neonatal Nurs JOGNN. 2004;33: 801–808. doi:10.1177/0884217504270598

67. Coffman JM, Cabana MD, Yelin EH. Do School-Based Asthma Education Programs Improve Self-Management and Health Outcomes? Pediatrics. 2009;124: 729–742. doi:10.1542/peds.2008-2085

68. Cooper Robbins SC, Ward K, Skinner SR. School-based vaccination: a systematic review of process evaluations. Vaccine. 2011;29: 9588–9599. doi:10.1016/j.vaccine.2011.10.033

69. Corcoran J, Dattalo P, Crowley M, Brown E, Grindle L. A systematic review of psychosocial interventions for suicidal adolescents. Child Youth Serv Rev. 2011;33: 2112–2118. doi:10.1016/j.childyouth.2011.06.017

70. Coren E, Barlow J. Individual and group‐based parenting programmes for improving psychosocial outcomes for teenage parents and their children. The Cochrane Library. John Wiley & Sons, Ltd; 2001. Available: http://cochranelibrary-wiley.com/doi/10.1002/14651858.CD002964/abstract

71. Cornell HR, Lin TT, Anderson JA. A systematic review of play-based interventions for students with ADHD: implications for school-based occupational therapists. J Occup Ther Sch Early Interv. 2018;11: 192–211. doi:10.1080/19411243.2018.1432446

72. Corrieri S, Heider D, Conrad I, Blume A, König H-H, Riedel-Heller SG. School-based prevention programs for depression and anxiety in adolescence: a systematic review. Health Promot Int. 2014;29: 427–441. doi:10.1093/heapro/dat001

73. Cuijpers P. Effective ingredients of school-based drug prevention programs. A systematic review. Addict Behav. 2002;27: 1009–1023.

74. Cuijpers P, van Straten A, Smits N, Smit F. Screening and early psychological intervention for depression in schools. Eur Child Adolesc Psychiatry. 2006;15: 300–307. doi:10.1007/s00787-006-0537-4

75. Curran T, Wexler L. School-Based Positive Youth Development: A Systematic Review of the Literature. J Sch Health. 2017;87: 71–80. doi:10.1111/josh.12467

76. Cushing CC, Brannon EE, Suorsa KI, Wilson DK. Systematic Review and Meta-Analysis of Health Promotion Interventions for Children and Adolescents Using an Ecological Framework. J Pediatr Psychol. 2014;39: 949–962. doi:10.1093/jpepsy/jsu042

77. Cusimano MD, Sameem M. The effectiveness of middle and high school-based suicide prevention programmes for adolescents: a systematic review. Inj Prev. 2011;17: 43–49. doi:10.1136/ip.2009.025502

78. Dart EH, Radley KC, Mason BA, Allen JP. Addressing escape-maintained behavior for students with developmental disabilities: A systematic review of school-based interventions. Psychol Sch. 2018;55: 295–304. doi:10.1002/pits.22108

79. Das JK, Salam RA, Arshad A, Lassi ZS, Bhutta ZA. Systematic Review and Meta-Analysis of Interventions to Improve Access and Coverage of Adolescent Immunizations. J Adolesc Health. 2016;59: S40–S48. doi:10.1016/j.jadohealth.2016.07.005

80. Davo MC, Gil-Gonzalez D, Vives-Cases C, Alvarez-Dardet C, La Parra D. Research on health education and promotion in Spanish nursery and primary schools. A systematic review of studies published between 1995 and 2005. Gac Sanit. 2008;22: 58–64.

81. Daykin N, Orme J, Evans D, Salmon D, McEachran M, Brain S. The impact of participation in performing arts on adolescent health and behaviour: a systematic review of the literature. J Health Psychol. 2008;13: 251–264. doi:10.1177/1359105307086699

82. De Bourdeaudhuij I, Van Cauwenberghe E, Spittaels H, Oppert J-M, Rostami C, Brug J, et al. School-based interventions promoting both physical activity and healthy eating in Europe: a systematic review within the HOPE project. Obes Rev Off J Int Assoc Study Obes. 2011;12: 205–216. doi:10.1111/j.1467-789X.2009.00711.x

83. de Kleijn MJJ, Farmer MM, Booth M, Motala A, Smith A, Sherman S, et al. Systematic review of school-based interventions to prevent smoking for girls. Syst Rev. 2015;4: 109. doi:10.1186/s13643-015-0082-7

84. De Koker P, Mathews C, Zuch M, Bastien S, Mason-Jones AJ. A systematic review of interventions for preventing adolescent intimate partner violence. J Adolesc Health Off Publ Soc Adolesc Med. 2014;54: 3–13. doi:10.1016/j.jadohealth.2013.08.008

85. De Meester F, van Lenthe FJ, Spittaels H, Lien N, De Bourdeaudhuij I. Interventions for promoting physical activity among European teenagers: a systematic review. Int J Behav Nutr Phys Act. 2009;6: 82. doi:10.1186/1479-5868-6-82

86. de Sa J, Lock K. Will European agricultural policy for school fruit and vegetables improve public health? A review of school fruit and vegetable programmes. Eur J Public Health. 2008;18: 558–568. doi:10.1093/eurpub/ckn061

87. De-Regil LM, Jefferds MED, Sylvetsky AC, Dowswell T. Intermittent iron supplementation for improving nutrition and development in children under 12 years of age. Cochrane Database of Systematic Reviews. John Wiley & Sons, Ltd; 2011. Available: http://onlinelibrary.wiley.com/doi/10.1002/14651858.CD009085.pub2/abstract

88. Dean SV, Lassi ZS, Imam AM, Bhutta ZA. Preconception care: promoting reproductive planning. Reprod Health. 2014;11 Suppl 3: S2. doi:10.1186/1742-4755-11-S3-S2

89. Delgado-Noguera M, Tort S, Martinez-Zapata MJ, Bonfill X. Primary school interventions to promote fruit and vegetable consumption: A systematic review and meta-analysis. Prev Med. 2011;53: 3–9. doi:10.1016/j.ypmed.2011.04.016

90. Denno DM, Chandra-Mouli V, Osman M. Reaching youth with out-of-facility HIV and reproductive health services: a systematic review. J Adolesc Health Off Publ Soc Adolesc Med. 2012;51: 106–121. doi:10.1016/j.jadohealth.2012.01.004

91. Demetriou Y, Höner O. Physical activity interventions in the school setting: A systematic review. Psychol Sport Exerc. 2012;13: 186–196. doi:10.1016/j.psychsport.2011.11.006

92. Demetriou Y, Sudeck G, Thiel A, Hoener O. The effects of school-based physical activity interventions on students’ health-related fitness knowledge: A systematic review. Educ Res Rev. 2015;16: 19–40. doi:10.1016/j.edurev.2015.07.002

93. DiCenso A, Guyatt G, Willan A, Griffith L. Interventions to reduce unintended pregnancies among adolescents: systematic review of randomised controlled trials. BMJ. 2002;324: 1426.

94. DiCenso A, Guyatt GH, Willan A, Ontario, Public Health Research E and DP. A systematic review of the effectiveness of adolescent pregnancy primary prevention programs. Hamilton, ON: Region of Hamilton-Wentworth, Social and Public Health Services Division, Community Support and Research Branch, PHRED Program; 1999.

95. Dobbins M, De Corby K, Robeson P, Husson H, Tirilis D. School-based physical activity programs for promoting physical activity and fitness in children and adolescents aged 6-18. Cochrane Database Syst Rev. 2009; CD007651. doi:10.1002/14651858.CD007651

96. Dobbins M, Husson H, DeCorby K, LaRocca RL. School-based physical activity programs for promoting physical activity and fitness in children and adolescents aged 6 to 18. Cochrane Database Syst Rev. 2013; CD007651. doi:10.1002/14651858.CD007651.pub2

97. Donaldson SI, Sussman S, MacKinnon DP, Severson HH, Glynn T, Murray DM, et al. Drug abuse prevention programming: Do we know what content works? Am Behav Sci. 1996; 868–883.

98. Driessen CE, Cameron AJ, Thornton LE, Lai SK, Barnett LM. Effect of changes to the school food environment on eating behaviours and/or body weight in children: a systematic review. Obes Rev Off J Int Assoc Study Obes. 2014;15: 968–982. doi:10.1111/obr.12224

99. Dudley DA, Cotton WG, Peralta LR. Teaching approaches and strategies that promote healthy eating in primary school children: a systematic review and meta-analysis. Int J Behav Nutr Phys Act. 2015;12: 28. doi:10.1186/s12966-015-0182-8

100. Duperrex O, Blackhall K, Burri M, Jeannot E. Education of children and adolescents for the prevention of dog bite injuries. Cochrane Database Syst Rev. 2009; CD004726. doi:10.1002/14651858.CD004726.pub2

101. Eccleston C, Morley S, Williams A, Yorke L, Mastroyannopoulou K. Systematic review of randomised controlled trials of psychological therapy for chronic pain in children and adolescents, with a subset meta-analysis of pain relief. Pain. 2002;99: 157–165.

102. Edwards D, Noyes J, Lowes L, Haf Spencer L, Gregory JW. An ongoing struggle: a mixed-method systematic review of interventions, barriers and facilitators to achieving optimal self-care by children and young people with type 1 diabetes in educational settings. BMC Pediatr. 2014;14: 228. doi:10.1186/1471-2431-14-228

103. Ejemot-Nwadiaro RI, Ehiri JE, Arikpo D, Meremikwu MM, Critchley JA. Hand washing promotion for preventing diarrhoea. Cochrane Database of Systematic Reviews. John Wiley & Sons, Ltd; 2015. Available: http://onlinelibrary.wiley.com/doi/10.1002/14651858.CD004265.pub3/abstract

104. Ekeland E, Heian F, Hagen KB, Abbott J, Nordheim L. Exercise to improve self-esteem in children and young people. Cochrane Database Syst Rev. 2004; CD003683. doi:10.1002/14651858.CD003683.pub2

105. Eklund K, Rossen E, Koriakin T, Chafouleas SM, Resnick C. A Systematic Review of Trauma Screening Measures for Children and Adolescents. Sch Psychol Q. 2018;33: 30–43. doi:10.1037/spq0000244

106. El Dib RP, Atallah AN, Andriolo RB, de Oliveira Soares BG, Verbeek JH. A systematic review of the interventions to promote the wearing of hearing protection. Sao Paulo Med J. 2007;125: 362–369.

107. Elder RW, Nichols JL, Shults RA, Sleet DA, Barrios LC, Compton R. Effectiveness of school-based programs for reducing drinking and driving and riding with drinking drivers - A systematic review. Am J Prev Med. 2005;28: 288–304. doi:10.1016/j.amepre.2005.02.015

108. Elliott L, Orr L, Watson L, Jackson A. Secondary prevention interventions for young drug users: a systematic review of the evidence. Adolescence. 2005;40: 1–22.

109. Evans CEL, Christian MS, Cleghorn CL, Greenwood DC, Cade JE. Systematic review and meta-analysis of school-based interventions to improve daily fruit and vegetable intake in children aged 5 to 12 y. Am J Clin Nutr. 2012;96: 889–901. doi:10.3945/ajcn.111.030270

110. Evans CBR, Fraser MW, Cotter KL. The effectiveness of school-based bullying prevention programs: A systematic review. Aggress Violent Behav. 2014;19: 532–544. doi:10.1016/j.avb.2014.07.004

111. Faggiano F, Vigna-Taglianti FD, Versino E, Zambon A, Borraccino A, Lemma P. School-based prevention for illicit drugs use: A systematic review. Prev Med. 2008;46: 385–396. doi:10.1016/j.ypmed.2007.11.012

112. Faggiano F, Minozzi S, Versino E, Buscemi D. Universal school‐based prevention for illicit drug use. The Cochrane Library. John Wiley & Sons, Ltd; 2014. Available: http://onlinelibrary.wiley.com/doi/10.1002/14651858.CD003020.pub3/abstract

113. Fair KN, Williams KDS, Warren J, McKyer ELJ, Ory MG. The Influence of Organizational Culture on School-Based Obesity Prevention Interventions: A Systematic Review of the Literature. J Sch Health. 2018;88: 462–473. doi:10.1111/josh.12626

114. Farahmand FK, Grant KE, Polo AJ, Duffy SN, DuBois DL. School-Based Mental Health and Behavioral Programs for Low-Income, Urban Youth: A Systematic and Meta-Analytic Review. Clin Psychol-Sci Pract. 2011;18: 372–390. doi:10.1111/j.1468-2850.2011.01265.x

115. Farrington DP, Ttofi MM. School-based programs to reduce bullying and victimization. Campbell Syst Rev. 2009;6. Available: https://www.campbellcollaboration.org/media/k2/attachments/School-based_Anti-Bullying_Programs_v2_R.pdf

116. Feldman D, Beausejour M, Felix Sosa J, Goulet L, Parent S, Labelle H. Cost effectiveness of school screening for scoliosis: a systematic review. Int J Child Adolesc Health. 2014;7: 7–13.

117. Feng L, Wei D-M, Lin S-T, Maddison R, Mhurchu CN, Jiang Y, et al. Systematic review and meta-analysis of school-based obesity interventions in mainland China. Plos One. 2017;12: e0184704. doi:10.1371/journal.pone.0184704

118. Flay BR. School-based smoking prevention programs with the promise of long-term effects. Tob Induc Dis. 2009;5: 6. doi:10.1186/1617-9625-5-6

119. Flynn AB, Falco M, Hocini S. Independent Evaluation of Middle School-Based Drug Prevention Curricula A Systematic Review. Jama Pediatr. 2015;169: 1046–1052. doi:10.1001/jamapediatrics.2015.1736

120. Fonner VA, Armstrong KS, Kennedy CE, O’Reilly KR, Sweat MD. School Based Sex Education and HIV Prevention in Low- and Middle-Income Countries: A Systematic Review and Meta-Analysis. PLOS ONE. 2014;9: e89692. doi:10.1371/journal.pone.0089692

121. Fortnum H, Ukoumunne OC, Hyde C, Taylor RS, Ozolins M, Errington S, et al. A programme of studies including assessment of diagnostic accuracy of school hearing screening tests and a cost-effectiveness model of school entry hearing screening programmes. Health Technol Assess. 2016;20: 1–+. doi:10.3310/hta20360

122. Foster C, Kelly P, Reid HAB, Roberts N, Murtagh EM, Humphreys DK, et al. What works to promote walking at the population level? A systematic review. Br J Sports Med. 2018;52: 807–812. doi:10.1136/bjsports-2017-098953

123. Fothergill A, Satherley P, Webber I. A systematic review on the effectiveness of school nurse implemented mental health screening available for adolescents in schools. J Psychiatr Ment Health Nurs. 2003;10: 625–626.

124. Fowden K, Franklin R, Graves P, MacLaren D, McBride J. The prevalence of leprosy in school-students and evaluation of school-based screening for leprosy: A Systematic Review. Lepr Rev. 2016;87: 276–293.

125. Foxcroft DR, Ireland D, Lister-Sharp DJ, Lowe G, Breen R. Longer-term primary prevention for alcohol misuse in young people: a systematic review. Addict Abingdon Engl. 2003;98: 397–411.

126. Foxcroft DR, Lister-Sharp D, Lowe G. Alcohol misuse prevention for young people: a systematic review reveals methodological concerns and lack of reliable evidence of effectiveness. Addict Abingdon Engl. 1997;92: 531–537.

127. Foxcroft DR, Tsertsvadze A. Cochrane Review: Universal school-based prevention programs for alcohol misuse in young people. Evid-Based Child Health Cochrane Rev J. 2012;7: 450–575. doi:10.1002/ebch.1829

128. Foxcroft DR, Tsertsvadze A. Universal school-based prevention programs for alcohol misuse in young people. Cochrane Database Syst Rev. 2011; CD009113. doi:10.1002/14651858.CD009113

129. Franklin C, Grant D, Corcoran J, Miller PO, Bultman L. Effectiveness of Prevention Programs for Adolescent Pregnancy: A Meta-Analysis. J Marriage Fam. 1997;59: 551–567. doi:10.2307/353945

130. Galantino ML, Galbavy R, Quinn L. Therapeutic effects of yoga for children: a systematic review of the literature. Pediatr Phys Ther Off Publ Sect Pediatr Am Phys Ther Assoc. 2008;20: 66–80. doi:10.1097/PEP.0b013e31815f1208

131. Gallagher KE, Kadokura E, Eckert LO, Miyake S, Mounier-Jack S, Aldea M, et al. Factors influencing completion of multi-dose vaccine schedules in adolescents: a systematic review. Bmc Public Health. 2016;16: 172. doi:10.1186/s12889-016-2845-z

132. Gambhir RS, Sohi RK, Nanda T, Sawhney GS, Setia S. Impact of school based oral health education programmes in India: a systematic review. J Clin Diagn Res JCDR. 2013;7: 3107–3110. doi:10.7860/JCDR/2013/6212.3718

133. Gao Z, Chen S. Are field-based exergames useful in preventing childhood obesity? A systematic review. Obes Rev Off J Int Assoc Study Obes. 2014;15: 676–691. doi:10.1111/obr.12164

134. Gascoine L, Higgins S, Wall K. The assessment of metacognition in children aged 4-16 years: a systematic review. Rev Educ. 2017;5: 3–57. doi:10.1002/rev3.3077

135. Gavin LE, Catalano RF, David-Ferdon C, Gloppen KM, Markham CM. A Review of Positive Youth Development Programs That Promote Adolescent Sexual and Reproductive Health. J Adolesc Health. 2010;46: S75–S91. doi:10.1016/j.jadohealth.2009.11.215

136. Gavine AJ, Donnelly PD, Williams DJ. Effectiveness of universal school-based programs for prevention of violence in adolescents. Psychol Violence. 2016;6: 390–399. doi:10.1037/vio0000052

137. Gera T, Shah D, Sachdev HS. Impact of Water, Sanitation and Hygiene Interventions on Growth, Non-diarrheal Morbidity and Mortality in Children Residing in Low- and Middle-income Countries: A Systematic Review. Indian Pediatr. 2018;55: 381–393.

138. Glaser DB, Roberts KJ, Grosskopf NA, Basch CH. An Evaluation of the Effectiveness of School-Based Breastfeeding Education. J Hum Lact. 2016;32: 46–52. doi:10.1177/0890334415595040

139. Godin K, Leatherdale ST, Elton-Marshall T. A systematic review of the effectiveness of school-based obesity prevention programmes for First Nations, Inuit and Metis youth in Canada. Clin Obes. 2015;5: 103–115. doi:10.1111/cob.12099

140. Gonzalez-Suarez CB, Dones V. Evidence for the effectiveness of different school-based programs in the management of childhood obesity: a systematic review. JBI Libr Syst Rev. 2008;6: 1–9.

141. Gorga E, Regazzoni V, Bansilal S, Carubelli V, Trichaki E, Gavazzoni M, et al. School and family-based interventions for promoting a healthy lifestyle among children and adolescents in Italy: a systematic review. J Cardiovasc Med. 2016;17: 547–555. doi:10.2459/JCM.0000000000000404

142. Gould LF, Dariotis JK, Greenberg MT, Mendelson T. Assessing Fidelity of Implementation (FOI) for School-Based Mindfulness and Yoga Interventions: A Systematic Review. Mindfulness. 2016;7: 5–33. doi:10.1007/s12671-015-0395-6

143. Govindasamy D, Ferrand RA, Wilmore SMS, Ford N, Ahmed S, Afnan-Holmes H, et al. Uptake and yield of HIV testing and counselling among children and adolescents in sub-Saharan Africa: a systematic review. J Int Aids Soc. 2015;18: 20182. doi:10.7448/IAS.18.1.20182

144. Griebler U, Rojatz D, Simovska V, Forster R. Effects of student participation in school health promotion: a systematic review. Health Promot Int. 2017;32: 195–206. doi:10.1093/heapro/dat090

145. Griffin SO, Naavaal S, Scherrer C, Patel M, Chattopadhyay S. Evaluation of School-Based Dental Sealant Programs: An Updated Community Guide Systematic Economic Review. Am J Prev Med. 2017;52: 407–415. doi:10.1016/j.amepre.2016.10.004

146. Haddison EC, Abdullahi LH, Muloiwa R, Hussey GD, Kagina BM. Comparison of school based and supplemental vaccination strategies in the delivery of vaccines to 5-19 year olds in Africa - a systematic review. F1000Research. 2017;6. doi:10.12688/f1000research.12804.1

147. Hahn R, Fuqua-Whitley D, Wethington H, Lowy J, Crosby A, Fullilove M, et al. Effectiveness of universal school-based programs to prevent violent and aggressive Behavior - A systematic review. Am J Prev Med. 2007;33: S114–S129. doi:10.1016/j.amepre.2007.04.012

148. Hale DR, Fitzgerald-Yau N, Viner RM. A systematic review of effective interventions for reducing multiple health risk behaviors in adolescence. Am J Public Health. 2014;104: e19–41. doi:10.2105/AJPH.2014.301874

149. Harden A, Rees R, Shepherd J, Brunton G, Oliver S, Oakley A. Young People and Mental Health: A Systematic Review of Research on Barriers and Facilitators. [Internet]. London: EPPI-Centre, Social Science Research Unit, Institute of Education, University of London.; 2001. Available: https://eppi.ioe.ac.uk/cms/Default.aspx?tabid=256

150. Harden A, Brunton G, Fletcher A, Oakley A. Teenage pregnancy and social disadvantage: systematic review integrating controlled trials and qualitative studies. BMJ. 2009;339: b4254. doi:10.1136/bmj.b4254

151. Harrington R, Whittaker J, Shoebridge P, Campbell F. Systematic review of efficacy of cognitive behaviour therapies in childhood and adolescent depressive disorder. BMJ. 1998;316: 1559–1563. doi:10.1136/bmj.316.7144.1559

152. Harris KC, Kuramoto LK, Schulzer M, Retallack JE. Effect of school-based physical activity interventions on body mass index in children: a meta-analysis. CMAJ Can Med Assoc J J Assoc Medicale Can. 2009;180: 719–726. doi:10.1503/cmaj.080966

153. Harrison A, Newell M-L, Imrie J, Hoddinott G. HIV prevention for South African youth: which interventions work? A systematic review of current evidence. BMC Public Health. 2010;10: 102. doi:10.1186/1471-2458-10-102

154. Hartmann-Boyce J, Stead LF, Cahill K, Lancaster T. Efficacy of interventions to combat tobacco addiction: Cochrane update of 2013 reviews. Addict Abingdon Engl. 2014;109: 1414–1425. doi:10.1111/add.12633

155. Hawton K, Townsend E, Arensman E, Gunnell D, Hazell P, House A, et al. Psychosocial versus pharmacological treatments for deliberate self harm. Cochrane Database Syst Rev. 2000; CD001764. doi:10.1002/14651858.CD001764

156. Heerde JA, Hemphill SA. Examination of associations between informal help-seeking behavior, social support, and adolescent psychosocial outcomes: A meta-analysis. Dev Rev. 2018;47: 44–62. doi:10.1016/j.dr.2017.10.001

157. Hegarty LM, Mair JL, Kirby K, Murtagh E, Murphy MH. School-based Interventions to Reduce Sedentary Behaviour in Children: A Systematic Review. Aims Public Health. 2016;3: 520–541. doi:10.3934/publichealth.2016.3.520

158. Hersch D, Perdue L, Ambroz T, Boucher JL. The impact of cooking classes on food-related preferences, attitudes, and behaviors of school-aged children: a systematic review of the evidence, 2003-2014. Prev Chronic Dis. 2014;11: E193. doi:10.5888/pcd11.140267

159. Hillier-Brown FC, Bambra CL, Cairns J-M, Kasim A, Moore HJ, Summerbell CD. A systematic review of the effectiveness of individual, community and societal level interventions at reducing socioeconomic inequalities in obesity amongst children. BMC Public Health. 2014;14: 834. doi:10.1186/1471-2458-14-834

160. Hodder RK, Freund M, Wolfenden L, Bowman J, Nepal S, Dray J, et al. Systematic review of universal school-based “resilience” interventions targeting adolescent tobacco, alcohol or illicit substance use: A meta-analysis. Prev Med. 2017;100: 248–268. doi:10.1016/j.ypmed.2017.04.003

161. Hoehner CM, Ribeiro IC, Parra DC, Reis RS, Azevedo MR, Hino AA, et al. Physical Activity Interventions in Latin America: Expanding and Classifying the Evidence. Am J Prev Med. 2013;44: e31–e40. doi:10.1016/j.amepre.2012.10.026

162. Hoehner CM, Soares J, Parra Perez D, Ribeiro IC, Joshu CE, Pratt M, et al. Physical activity interventions in Latin America: a systematic review. Am J Prev Med. 2008;34: 224–233. doi:10.1016/j.amepre.2007.11.016

163. Hollis JL, Williams AJ, Sutherland R, Campbell E, Nathan N, Wolfenden L, et al. A systematic review and meta-analysis of moderate-to-vigorous physical activity levels in elementary school physical education lessons. Prev Med. 2016;86: 34–54. doi:10.1016/j.ypmed.2015.11.018

164. Holly C, Porter S, Kamienski M, Lim A. School-Based and Community-Based Gun Safety Educational Strategies for Injury Prevention. Health Promot Pract. 2018; 1524839918774571. doi:10.1177/1524839918774571

165. Holub CK, Elder JP, Arredondo EM, Barquera S, Eisenberg CM, Romero LMS, et al. Obesity Control in Latin American and U.S. Latinos. Am J Prev Med. 2013;44: 529–537. doi:10.1016/j.amepre.2013.01.023

166. Holub CK, Lobelo F, Mehta SM, Sánchez Romero LM, Arredondo EM, Elder JP. School-wide programs aimed at obesity among Latino youth in the United States: a review of the evidence. J Sch Health. 2014;84: 239–246. doi:10.1111/josh.12144

167. Hoyland A, Dye L, Lawton CL. A systematic review of the effect of breakfast on the cognitive performance of children and adolescents. Nutr Res Rev. 2009;22: 220–243. doi:10.1017/S0954422409990175

168. Hynynen S-T, van Stralen MM, Sniehotta FF, Araujo-Soares V, Hardeman W, Chinapaw MJM, et al. A systematic review of school-based interventions targeting physical activity and sedentary behaviour among older adolescents. Int Rev Sport Exerc Psychol. 2016;9: 22–44. doi:10.1080/1750984X.2015.1081706

169. Ickes MJ, Erwin H, Beighle A. Systematic Review of Recess Interventions to Increase Physical Activity. J Phys Act Health. 2013;10: 910–926. doi:10.1123/jpah.10.6.910

170. Ingram G. School-Based Activity Programs for Promoting Physical Activity and Fitness in Children and Adolescents Aged 6-18 Years. Int J Child Adolesc Health. 2011;4: 107.

171. Isaac M, Elias B, Katz LY, Belik S-L, Deane FP, Enns MW, et al. Gatekeeper training as a preventative intervention for suicide: a systematic review. Can J Psychiatry Rev Can Psychiatr. 2009;54: 260–268. doi:10.1177/070674370905400407

172. Isensee B, Hanewinkel R. Meta-analysis on the effects of the smoke-free class competition on smoking prevention in adolescents. Eur Addict Res. 2012;18: 110–115. doi:10.1159/000335085

173. Jackson C, Geddes R, Haw S, Frank J. Interventions to prevent substance use and risky sexual behaviour in young people: a systematic review. Addict Abingdon Engl. 2012;107: 733–747. doi:10.1111/j.1360-0443.2011.03751.x

174. Jackson C, Haw SJ, Frank J. Adolescent and Young Adult Health in Scotland - Interventions that address multiple risk behaviours or take a generic approach to risk in youth [Internet]. SCPHRP; 2010. Available: https://www.research.ed.ac.uk/portal/en/publications/adolescent-and-young-adult-health-in-scotland--interventions-that-address-multiple-risk-behaviours-or-take-a-generic-approach-to-risk-in-youth(044214da-0ff2-4ad0-9f31-1aff34aa6a3b).html

175. Jacob V, Chattopadhyay SK, Hopkins DP, Morgan JM, Pitan AA, Clymer JM. Increasing Coverage of Appropriate Vaccinations A Community Guide Systematic Economic Review. Am J Prev Med. 2016;50: 797–808. doi:10.1016/j.amepre.2015.11.003

176. Jacobson Vann JC, Jacobson RM, Coyne‐Beasley T, Asafu‐Adjei JK, Szilagyi PG. Patient reminder and recall interventions to improve immunization rates. The Cochrane Library. John Wiley & Sons, Ltd; 2018. Available: http://cochranelibrary-wiley.com/doi/10.1002/14651858.CD003941.pub3/full

177. Jago R, Baranowski T. Non-curricular approaches for increasing physical activity in youth: a review. Prev Med. 2004;39: 157–163. doi:10.1016/j.ypmed.2004.01.014

178. Jamil MS, Bauer HM, Hocking JS, Ali H, Wand H, Walker J, et al. Chlamydia Screening Strategies and Outcomes in Educational Settings: A Systematic Review. Sex Transm Dis. 2014;41: 180–187. doi:10.1097/OLQ.0000000000000095

179. Jenkinson KA, Naughton G, Benson AC. Peer-Assisted Learning in School Physical Education, Sport and Physical Activity Programmes: A Systematic Review. Phys Educ Sport Pedagogy. 2014;19: 253–277. doi:10.1080/17408989.2012.754004

180. Jordans MJD, Tol WA, Komproe IH, De Jong JVTM. Systematic Review of Evidence and Treatment Approaches: Psychosocial and Mental Health Care for Children in War. Child Adolesc Ment Health. 2009;14: 2–14. doi:10.1111/j.1475-3588.2008.00515.x

181. Joury E, Bernabe E, Sabbah W, Nakhleh K, Gurusamy K. Systematic review and meta-analysis of randomised controlled trials on the effectiveness of school-based dental screening versus no screening on improving oral health in children. J Dent. 2017;58: 1–10. doi:10.1016/j.jdent.2016.11.008

182. Joury E, Bernabe E, Sabbah W, Nakhleh K, Gurusamy K. Systematic review and meta-analysis of randomised controlled trials on the effectiveness of school-based dental screening versus no screening on improving oral health in children. J Dent. 2017;58: 1–10. doi:10.1016/j.jdent.2016.11.008

183. Kahn EB, Ramsey LT, Brownson RC, Heath GW, Howze EH, Powell KE, et al. The effectiveness of interventions to increase physical activity. A systematic review. Am J Prev Med. 2002;22: 73–107.

184. Kamath CC, Vickers KS, Ehrlich A, McGovern L, Johnson J, Singhal V, et al. Behavioral Interventions to Prevent Childhood Obesity: A Systematic Review and Metaanalyses of Randomized Trials. J Clin Endocrinol Metab. 2008;93: 4606–4615. doi:10.1210/jc.2006-2411

185. Kang M, Marshall SJ, Barreira TV, Lee J-O. Effect of Pedometer-Based Physical Activity Interventions. Res Q Exerc Sport. 2009;80: 648–655. doi:10.1080/02701367.2009.10599604

186. Katz C, Bolton S-L, Katz LY, Isaak C, Tilston-Jones T, Sareen J, et al. A systematic review of school-based suicide prevention programs. Depress Anxiety. 2013;30: 1030–1045. doi:10.1002/da.22114

187. Katz DL. School-Based Interventions for Health Promotion and Weight Control: Not Just Waiting on the World to Change. Annu Rev Public Health. 2009;30: 253–272. doi:10.1146/annurev.publhealth.031308.100307

188. Katz DL, O’Connell M, Njike VY, Yeh M-C, Nawaz H. Strategies for the prevention and control of obesity in the school setting: systematic review and meta-analysis. Int J Obes 2005. 2008;32: 1780–1789. doi:10.1038/ijo.2008.158

189. Kay E, Locker D. A systematic review of the effectiveness of health promotion aimed at improving oral health. Community Dent Health. 1998;15: 132–144.

190. Keen B, Blaszczynski A, Anjoul F. Systematic Review of Empirically Evaluated School-Based Gambling Education Programs. J Gambl Stud. 2017;33: 301–325. doi:10.1007/s10899-016-9641-7

191. Kellou N, Sandalinas F, Copin N, Simon C. Prevention of unhealthy weight in children by promoting physical activity using a socio-ecological approach: What can we learn from intervention studies? Diabetes Metab. 2014;40: 258–271. doi:10.1016/j.diabet.2014.01.002

192. Kelly S, Stephens J, Hoying J, McGovern C, Melnyk BM, Militello L. A systematic review of mediators of physical activity, nutrition, and screen time in adolescents: Implications for future research and clinical practice. Nurs Outlook. 2017;65: 530–548. doi:10.1016/j.outlook.2017.07.011

193. Kendrick D, Young B, Mason-Jones AJ, Ilyas N, Achana FA, Cooper NJ, et al. Home safety education and provision of safety equipment for injury prevention. Cochrane Database of Systematic Reviews. John Wiley & Sons, Ltd; 2012. Available: http://onlinelibrary.wiley.com/doi/10.1002/14651858.CD005014.pub3/abstract

194. Kessels SJM, Marshall HS, Watson M, Braunack-Mayer AJ, Reuzel R, Tooher RL. Factors associated with HPV vaccine uptake in teenage girls: A systematic review. Vaccine. 2012;30: 3546–3556. doi:10.1016/j.vaccine.2012.03.063

195. Khayyati F, Allahverdipour H, Shaghaghi A, Fathifar Z. Tobacco Use Prevention by Integrating Inside and Outside of School Based Programs: A Systematic Review Article. Health Promot Perspect. 2015;5: 81–91. doi:10.15171/hpp.2015.010

196. Kim N, Stanton B, Li X, Dickersin K, Galbraith J. Effectiveness of the 40 adolescent AIDS-risk reduction interventions: a quantitative review. J Adolesc Health Off Publ Soc Adolesc Med. 1997;20: 204–215. doi:10.1016/S1054-139X(96)00169-3

197. Kingsnorth S, Healy H, Macarthur C. Preparing for adulthood: a systematic review of life skill programs for youth with physical disabilities. J Adolesc Health Off Publ Soc Adolesc Med. 2007;41: 323–332. doi:10.1016/j.jadohealth.2007.06.007

198. Kirkland SW, Soleimani A, Newton AS. Review: The impact of pediatric mental health care provided outpatient, primary care, community and school settings on emergency department use - a systematic review. Child Adolesc Ment Health. 2018;23: 4–13. doi:10.1111/camh.12230

199. Kirkland SW, Soleimani A, Newton AS. Review: The impact of pediatric mental health care provided outpatient, primary care, community and school settings on emergency department use - a systematic review. Child Adolesc Ment Health. 2018;23: 4–13. doi:10.1111/camh.12230

200. Knai C, Pomerleau J, Lock K, McKee M. Getting children to eat more fruit and vegetables: a systematic review. Prev Med. 2006;42: 85–95. doi:10.1016/j.ypmed.2005.11.012

201. Knopf JA, Finnie RKC, Peng Y, Hahn RA, Truman BI, Vernon-Smiley M, et al. School-Based Health Centers to Advance Health Equity A Community Guide Systematic Review. Am J Prev Med. 2016;51: 114–126. doi:10.1016/j.amepre.2016.01.009

202. Knowlden AP, Sharma M. Systematic Review of School-based Obesity Interventions Targeting African American and Hispanic Children. J Health Care Poor Underserved. 2013;24: 1194–1214.

203. Kong K, Liu J, Tao Y. Limitations of studies on school-based nutrition education interventions for obesity in China: a systematic review and meta-analysis. Asia Pac J Clin Nutr. 2016;25: 589–601.

204. Kothandan SK. School based interventions versus family based interventions in the treatment of childhood obesity- a systematic review. Arch Public Health Arch Belg Sante Publique. 2014;72: 3. doi:10.1186/2049-3258-72-3

205. Krishnaswami J, Martinson M, Wakimoto P, Anglemeyer A. Community-engaged interventions on diet, activity, and weight outcomes in U.S. schools: a systematic review. Am J Prev Med. 2012;43: 81–91. doi:10.1016/j.amepre.2012.02.031

206. Kristjansson B, Petticrew M, MacDonald B, Krasevec J, Janzen L, Greenhalgh T, et al. School feeding for improving the physical and psychosocial health of disadvantaged students. The Cochrane Library. John Wiley & Sons, Ltd; 2007. Available: http://onlinelibrary.wiley.com/doi/10.1002/14651858.CD004676.pub2/abstract

207. Kropski JA, Keckley PH, Jensen GL. School-based obesity prevention programs: an evidence-based review. Obes Silver Spring Md. 2008;16: 1009–1018. doi:10.1038/oby.2008.29

208. Lösel F, Beelmann A. Effects of Child Skills Training in Preventing Antisocial Behavior: A Systematic Review of Randomized Evaluations. Ann Am Acad Pol Soc Sci. 2003;587: 84–109. doi:10.1177/0002716202250793

209. Lai SK, Costigan SA, Morgan PJ, Lubans DR, Stodden DF, Salmon J, et al. Do School-Based Interventions Focusing on Physical Activity, Fitness, or Fundamental Movement Skill Competency Produce a Sustained Impact in These Outcomes in Children and Adolescents? A Systematic Review of Follow-Up Studies. Sports Med. 2014;44: 67–79. doi:10.1007/s40279-013-0099-9

210. Laine J, Kuvaja-Köllner V, Pietilä E, Koivuneva M, Valtonen H, Kankaanpää E. Cost-effectiveness of population-level physical activity interventions: a systematic review. Am J Health Promot AJHP. 2014;29: 71–80. doi:10.4278/ajhp.131210-LIT-622

211. Lane H, Porter K, Estabrooks P, Zoellner J. A Systematic Review to Assess Sugar-Sweetened Beverage Interventions for Children and Adolescents across the Socioecological Model. J Acad Nutr Diet. 2016;116: 1295–1307.e6. doi:10.1016/j.jand.2016.04.015

212. Langford R, Bonell C, Jones H, Pouliou T, Murphy S, Waters E, et al. The World Health Organization’s Health Promoting Schools framework: a Cochrane systematic review and meta-analysis. Bmc Public Health. 2015;15: 130. doi:10.1186/s12889-015-1360-y

213. Langford R, Bonell CP, Jones HE, Pouliou T, Murphy SM, Waters E, et al. The WHO Health Promoting School framework for improving the health and well-being of students and their academic achievement. Cochrane Database of Systematic Reviews. John Wiley & Sons, Ltd; 2014. Available: http://onlinelibrary.wiley.com/doi/10.1002/14651858.CD008958.pub2/abstract

214. Larun L, Nordheim LV, Ekeland E, Hagen KB, Heian F. Exercise in prevention and treatment of anxiety and depression among children and young people. Cochrane Database Syst Rev. 2006; CD004691. doi:10.1002/14651858.CD004691.pub2

215. Lavelle HV, Mackay DF, Pell JP. Systematic review and meta-analysis of school-based interventions to reduce body mass index. J Public Health. 2012;34: 360–369. doi:10.1093/pubmed/fdr116

216. Lee C, Robinson JL. Systematic review of the effect of immunization mandates on uptake of routine childhood immunizations. J Infect. 2016;72: 659–666. doi:10.1016/j.jinf.2016.04.002

217. Lee NK, Cameron J, Battams S, Roche A. What Works in School-Based Alcohol Education: A Systematic Review. Health Educ J. 2016;75: 780–798. doi:10.1177/0017896915612227

218. Leff S. Systematic review of the school entry medical examination. Arch Dis Child. 1999;80: 101–101.

219. Lemstra M, Bennett N, Nannapaneni U, Neudorf C, Warren L, Kershaw T, et al. A systematic review of school-based marijuana and alcohol prevention programs targeting adolescents aged 10-15. PubMed Health. 2010; Available: https://www.ncbi.nlm.nih.gov/pubmedhealth/PMH0029168/

220. Leroy ZC, Wallin R, Lee S. The Role of School Health Services in Addressing the Needs of Students With Chronic Health Conditions: A Systematic Review. J Sch Nurs. 2017;33: 64–72. doi:10.1177/1059840516678909

221. Lew RM, Burnett L, Proos AL, Barlow-Stewart K, Delatycki MB, Bankier A, et al. Ashkenazi Jewish population screening for Tay-Sachs disease: The International and Australian experience. J Paediatr Child Health. 2015;51: 271–279. doi:10.1111/jpc.12632

222. Li M, Li S, Baur LA, Huxley RR. A systematic review of school-based intervention studies for the prevention or reduction of excess weight among Chinese children and adolescents. Obes Rev Off J Int Assoc Study Obes. 2008;9: 548–559. doi:10.1111/j.1467-789X.2008.00495.x

223. Li Q, Babor TF, Zeigler D, Xuan Z, Morisky D, Hovell MF, et al. Health promotion interventions and policies addressing excessive alcohol use: a systematic review of national and global evidence as a guide to health-care reform in China. Addiction. 2015;110: 68–78. doi:10.1111/add.12784

224. Li Q, Babor TF, Zeigler D, Xuan Z, Morisky D, Hovell MF, et al. Health promotion interventions and policies addressing excessive alcohol use: a systematic review of national and global evidence as a guide to health-care reform in China. Addiction. 2015;110: 68–78. doi:10.1111/add.12784

225. Lima-Serrano M, Lima-Rodriguez JS. Impact of school-based health promotion interventions aimed at different behavioral domains: a systematic review. Gac Sanit. 2014;28: 411–417. doi:10.1016/j.gaceta.2014.05.003

226. Limbos MA, Chan LS, Warf C, Schneir A, Iverson E, Shekelle P, et al. Effectiveness of Interventions to Prevent Youth Violence: A Systematic Review. Am J Prev Med. 2007;33: 65–74. doi:10.1016/j.amepre.2007.02.045

227. Lindsay S, Hartman LR, Reed N, Gan C, Thomson N, Solomon B. A Systematic Review of Hospital-to-School Reintegration Interventions for Children and Youth with Acquired Brain Injury. PloS One. 2015;10: e0124679. doi:10.1371/journal.pone.0124679

228. Lineberry MJ, Ickes MJ. The Role and Impact of Nurses in American Elementary Schools: A Systematic Review of the Research. J Sch Nurs. 2015;31: 22–33. doi:10.1177/1059840514540940

229. Lister-Sharp D, Chapman S, Stewart-Brown S, Sowden A. Health promoting schools and health promotion in schools: two systematic reviews [Internet]. Centre for Reviews and Dissemination (UK); 1999. Available: https://www.ncbi.nlm.nih.gov/books/NBK67984/

230. Liu M, Wu L, Ming Q. How Does Physical Activity Intervention Improve Self-Esteem and Self-Concept in Children and Adolescents? Evidence from a Meta-Analysis. PloS One. 2015;10: e0134804. doi:10.1371/journal.pone.0134804

231. Lobelo F, Garcia de Quevedo I, Holub CK, Nagle BJ, Arredondo EM, Barquera S, et al. School-Based Programs Aimed at the Prevention and Treatment of Obesity: Evidence-Based Interventions for Youth in Latin America. J Sch Health. 2013;83: 668–677. doi:10.1111/josh.12080

232. Lofton S, Julion WA, McNaughton DB, Bergren MD, Keim KS. A Systematic Review of Literature on Culturally Adapted Obesity Prevention Interventions for African American Youth. J Sch Nurs Off Publ Natl Assoc Sch Nurses. 2016;32: 32–46. doi:10.1177/1059840515605508

233. Loharikar A, Suragh TA, MacDonald NE, Balakrishnan MR, Benes O, Lamprianou S, et al. Anxiety-related adverse events following immunization (AEFI): A systematic review of published clusters of illness. Vaccine. 2018;36: 299–305. doi:10.1016/j.vaccine.2017.11.017

234. Loke AY, Kwan ML, Wong Y-T, Wong AKY. The Uptake of Human Papillomavirus Vaccination and Its Associated Factors Among Adolescents: A Systematic Review. J Prim Care Community Health. 2017;8: 349–362. doi:10.1177/2150131917742299

235. Lopez LM, Bernholc A, Chen M, Tolley EE. School‐based interventions for improving contraceptive use in adolescents. The Cochrane Library. John Wiley & Sons, Ltd; 2016. Available: http://onlinelibrary.wiley.com/doi/10.1002/14651858.CD012249/abstract

236. Lopez LM, Grey TW, Chen M, Tolley EE, Stockton LL. Theory‐based interventions for contraception. The Cochrane Library. John Wiley & Sons, Ltd; 2016. Available: http://onlinelibrary.wiley.com/doi/10.1002/14651858.CD007249.pub5/abstract

237. Lopez LM, Otterness C, Chen M, Steiner M, Gallo MF. Behavioral interventions for improving condom use for dual protection. The Cochrane Library. John Wiley & Sons, Ltd; 2013. Available: http://onlinelibrary.wiley.com/doi/10.1002/14651858.CD010662.pub2/abstract

238. Low M, Farrell A, Biggs B-A, Pasricha S-R. Effects of daily iron supplementation in primary-school-aged children: systematic review and meta-analysis of randomized controlled trials. CMAJ Can Med Assoc J J Assoc Medicale Can. 2013;185: E791–802. doi:10.1503/cmaj.130628

239. Lynas J, Hawkins R. Fidelity in school-based child sexual abuse prevention programs: A systematic review. Child Abuse Negl. 2017;72: 10–21. doi:10.1016/j.chiabu.2017.07.003

240. Lytle L. Nutrition education for school-aged children. J Nutr Educ. 1995;27: 298–311. doi:10.1016/S0022-3182(12)80090-2

241. Macgowan MJ. Psychosocial Treatment of Youth Suicide: A Systematic Review of the Research. Res Soc Work Pract. 2004;14: 147–162. doi:10.1177/1049731503257889

242. Mann JJ, Apter A, Bertolote J, Beautrais A, Currier D, Haas A, et al. Suicide prevention strategies: a systematic review. JAMA. 2005;294: 2064–2074. doi:10.1001/jama.294.16.2064

243. Mariani L, Vici P, Suligoi B, Checcucci-Lisi G, Drury R. Early Direct and Indirect Impact of Quadrivalent HPV (4HPV) Vaccine on Genital Warts: a Systematic Review. Adv Ther. 2015;32: 10–30. doi:10.1007/s12325-015-0178-4

244. Marinho VC, Chong LY, Worthington HV, Walsh T. Fluoride mouthrinses for preventing dental caries in children and adolescents. The Cochrane Library. John Wiley & Sons, Ltd; 2016. Available: http://onlinelibrary.wiley.com/doi/10.1002/14651858.CD002284.pub2/abstract

245. Marques A, Gomez F, Martins J, Catunda R, Sarmento H. Association between physical education, school-based physical activity, and academic performance: a systematic review. Retos-Nuevas Tend En Educ Fis Deporte Recreacion. 2017; 316–320.

246. Marseille E, Mirzazadeh A, Biggs MA, P. Miller A, Horvath H, Lightfoot M, et al. Effectiveness of School-Based Teen Pregnancy Prevention Programs in the USA: a Systematic Review and Meta-Analysis. Prev Sci. 2018;19: 468–489. doi:10.1007/s11121-017-0861-6

247. Mason-Jones AJ, Crisp C, Momberg M, Koech J, De Koker P, Mathews C. A systematic review of the role of school-based healthcare in adolescent sexual, reproductive, and mental health. Syst Rev. 2012;1: 49. doi:10.1186/2046-4053-1-49

248. Mason‐Jones AJ, Sinclair D, Mathews C, Kagee A, Hillman A, Lombard C. School‐based interventions for preventing HIV, sexually transmitted infections, and pregnancy in adolescents. The Cochrane Library. John Wiley & Sons, Ltd; 2016. Available: http://onlinelibrary.wiley.com/doi/10.1002/14651858.CD006417.pub3/abstract

249. Matangila JR, Mitashi P, Inocêncio da Luz RA, Lutumba PT, Van Geertruyden J-P. Efficacy and safety of intermittent preventive treatment for malaria in schoolchildren: a systematic review. Malar J. 2015;14: 450. doi:10.1186/s12936-015-0988-5

250. Maxwell C, Aggleton P, Warwick I, Yankah E, Hill V, Mehmedbegović D. Supporting children’s emotional wellbeing and mental health in England: a review. Health Educ. 2008;108: 272–286. doi:10.1108/09654280810884160

251. Mbakaya BC, Lee PH, Lee RLT. Hand Hygiene Intervention Strategies to Reduce Diarrhoea and Respiratory Infections among Schoolchildren in Developing Countries: A Systematic Review. Int J Environ Res Public Health. 2017;14: 371. doi:10.3390/ijerph14040371

252. Mbuagbaw L, Ye C, Thabane L. Motivational interviewing for improving outcomes in youth living with HIV. Cochrane Database Syst Rev. 2012; CD009748. doi:10.1002/14651858.CD009748.pub2

253. McBride N. A systematic review of school drug education. Health Educ Res. 2003;18: 729–742.

254. McClain MB, Otero TL, Haverkamp CR, Molsberry F. Autism spectrum disorder assessment and evaluation research in 10 school psychology journals from 2007 to 2017. Psychol Sch. 2018;55: 661–679. doi:10.1002/pits.22133

255. Mcdaid D, Park A-L. Investing in mental health and well-being: findings from the DataPrev project. Health Promot Int. 2011;26 Suppl 1: i108–139. doi:10.1093/heapro/dar059

256. McDonald SM, Clennin MN, Pate RR. Specific Strategies for Promotion of Physical Activity in Kids-Which Ones Work? A Systematic Review of the Literature. Am J Lifestyle Med. 2018;12: 51–82. doi:10.1177/1559827615616381

257. McGinnis SM, McKeon T, Desai R, Ejelonu A, Laskowski S, Murphy HM. A Systematic Review: Costing and Financing of Water, Sanitation, and Hygiene (WASH) in Schools. Int J Environ Res Public Health. 2017;14: 442. doi:10.3390/ijerph14040442

258. McGuinness SL, Barker SF, O’Toole J, Cheng AC, Forbes AB, Sinclair M, et al. Effect of hygiene interventions on acute respiratory infections in childcare, school and domestic settings in low- and middle-income countries: a systematic review. Trop Med Int Health TM IH. 2018; doi:10.1111/tmi.13080

259. McQueston K, Silverman R, Glassman A. The Efficacy of Interventions to Reduce Adolescent Childbearing in Low- and Middle-Income Countries: A Systematic Review. Stud Fam Plann. 2013;44: 369–388. doi:10.1111/j.1728-4465.2013.00365.x

260. Meade CS, Ickovics JR. Systematic review of sexual risk among pregnant and mothering teens in the USA: pregnancy as an opportunity for integrated prevention of STD and repeat pregnancy. Soc Sci Med 1982. 2005;60: 661–678. doi:10.1016/j.socscimed.2004.06.015

261. Meadows E, Le Saux N. A systematic review of the effectiveness of antimicrobial rinse-free hand sanitizers for prevention of illness-related absenteeism in elementary school children. BMC Public Health. 2004;4: 50. doi:10.1186/1471-2458-4-50

262. Medina-Blanco RI, Jimenez-Cruz A, Perez-Morales ME, Armendariz-Anguiano AL, Bacardi-Gascon M. Intervention programs to promote physical activity in school children: systematic review. Nutr Hosp. 2011;26: 265–270. doi:10.3305/nh.2011.26.2.5180

263. Mellor C. School-based interventions targeting stigma of mental illness: systematic review. Psychiatr Bull 2014. 2014;38: 164–171. doi:10.1192/pb.bp.112.041723

264. Merry S, McDowell H, Hetrick S, Bir J, Muller N. Psychological and/or educational interventions for the prevention of depression in children and adolescents. Cochrane Database Syst Rev. 2004; CD003380. doi:10.1002/14651858.CD003380.pub2

265. Metcalf B, Henley W, Wilkin T. Effectiveness of intervention on physical activity of children: systematic review and meta-analysis of controlled trials with objectively measured outcomes (EarlyBird 54). BMJ. 2012;345: e5888. doi:10.1136/bmj.e5888

266. Michielsen K, Chersich MF, Luchters S, De Koker P, Van Rossem R, Temmerman M. Effectiveness of HIV prevention for youth in sub-Saharan Africa: systematic review and meta-analysis of randomized and nonrandomized trials. AIDS Lond Engl. 2010;24: 1193–1202. doi:10.1097/QAD.0b013e3283384791

267. Minatto G, Barbosa Filho VC, Berria J, Petroski EL. School-Based Interventions to Improve Cardiorespiratory Fitness in Adolescents: Systematic Review with Meta-analysis. Sports Med. 2016;46: 1273–1292. doi:10.1007/s40279-016-0480-6

268. Mirzazadeh A, Biggs MA, Viitanen A, Horvath H, Wang LY, Dunville R, et al. Do School-Based Programs Prevent HIV and Other Sexually Transmitted Infections in Adolescents? A Systematic Review and Meta-analysis. Prev Sci. 2018;19: 490–506. doi:10.1007/s11121-017-0830-0

269. Mirzazadeh A, Biggs MA, Viitanen A, Horvath H, Wang LY, Dunville R, et al. Do School-Based Programs Prevent HIV and Other Sexually Transmitted Infections in Adolescents? A Systematic Review and Meta-analysis. Prev Sci. 2018;19: 490–506. doi:10.1007/s11121-017-0830-0

270. Montoya ID, Atkinson J, McFaden WC. Best characteristics of adolescent gateway drug prevention programs. J Addict Nurs. 2003;14: 75–83. doi:10.1080/10884600390230466

271. Moore GF, Littlecott HJ, Turley R, Waters E, Murphy S. Socioeconomic gradients in the effects of universal school-based health behaviour interventions: a systematic review of intervention studies. BMC Public Health. 2015;15: 907. doi:10.1186/s12889-015-2244-x

272. Mura G, Vellante M, Nardi AE, Machado S, Carta MG. Effects of School-Based Physical Activity Interventions on Cognition and Academic Achievement: A Systematic Review. CNS Neurol Disord Drug Targets. 2015;14: 1194–1208.

273. Murray NG, Low BJ, Hollis C, Cross AW, Davis SM. Coordinated School Health Programs and Academic Achievement: A Systematic Review of the Literature. J Sch Health. 2007;77: 589–600.

274. Mytton J, Diguiseppi C, A Gough D, S Taylor R, Logan S. School-based violence prevention programs: Systematic review of secondary prevention trials. Arch Pediatr Adolesc Med. 2002;156: 752–62.

275. Mytton J, DiGuiseppi C, Gough D, Taylor R, Logan S. School-based secondary prevention programmes for preventing violence. Cochrane Database Syst Rev. 2006; CD004606. doi:10.1002/14651858.CD004606.pub2

276. Napierala Mavedzenge SM, Doyle AM, Ross DA. HIV prevention in young people in sub-Saharan Africa: a systematic review. J Adolesc Health Off Publ Soc Adolesc Med. 2011;49: 568–586. doi:10.1016/j.jadohealth.2011.02.007

277. National Health and Medical Research Council. Child Health Surveillance and Screening: A Critical Review of the Evidence [Internet]. Centre for Community Child Health, Royal Children’s Hospital Melbourne; 2002. Available: https://www.nhmrc.gov.au/guidelines-publications/ch42

278. Naylor P-J, Nettlefold L, Race D, Hoy C, Ashe MC, Higgins JW, et al. Implementation of school based physical activity interventions: A systematic review. Prev Med. 2015;72: 95–115. doi:10.1016/j.ypmed.2014.12.034

279. Negrini S, Aulisa L, Ferraro C, Fraschini P, Masiero S, Simonazzi P, et al. Italian guidelines on rehabilitation treatment of adolescents with scoliosis or other spinal deformities. Eur Medicophysica. 2005;41: 183–201.

280. Neil AL, Christensen H. Australian school-based prevention and early intervention programs for anxiety and depression: a systematic review. Med J Aust. 2007;186: 305–308.

281. Niccolai LM, Hansen CE. Practice- and Community-Based Interventions to Increase Human Papillomavirus Vaccine Coverage A Systematic Review. Jama Pediatr. 2015;169: 686–692. doi:10.1001/jamapediatrics.2015.0310

282. Niemeier BS, Hektner JM, Enger KB. Parent participation in weight-related health interventions for children and adolescents: a systematic review and meta-analysis. Prev Med. 2012;55: 3–13. doi:10.1016/j.ypmed.2012.04.021

283. Nishio A, Saito J, Tomokawa S, Kobayashi J, Makino Y, Akiyama T, et al. Systematic review of school tobacco prevention programs in African countries from 2000 to 2016. PloS One. 2018;13: e0192489. doi:10.1371/journal.pone.0192489

284. Nixon CA, Moore HJ, Douthwaite W, Gibson EL, Vogele C, Kreichauf S, et al. Identifying effective behavioural models and behaviour change strategies underpinning preschool- and school-based obesity prevention interventions aimed at 4-6-year-olds: a systematic review. Obes Rev. 2012;13: 106–117. doi:10.1111/j.1467-789X.2011.00962.x

285. Nixon CA, Moore HJ, Douthwaite W, Gibson EL, Vögele C, Kreichauf S, et al. A systematic review to identify behavioural models underpinning school-based interventions in pre-primary and primary settings for the prevention of obesity in children aged 4-6 years. Obes Rev Off J Int Assoc Study Obes. 2012;13. doi:10.1111/j.1467-789X.2011.00962.x

286. Nogueira RC, Weeks BK, Beck BR. Exercise to improve pediatric bone and fat: a systematic review and meta-analysis. Med Sci Sports Exerc. 2014;46: 610–621. doi:10.1249/MSS.0b013e3182a6ab0d

287. Nordheim LV, Gundersen MW, Espehaug B, Guttersrud O, Flottorp S. Effects of School-Based Educational Interventions for Enhancing Adolescents Abilities in Critical Appraisal of Health Claims: A Systematic Review. Plos One. 2016;11: e0161485. doi:10.1371/journal.pone.0161485

288. Norris E, Shelton N, Dunsmuir S, Duke-Williams O, Stamatakis E. Physically active lessons as physical activity and educational interventions: A systematic review of methods and results. Prev Med. 2015;72: 116–125. doi:10.1016/j.ypmed.2014.12.027

289. Oakley A, Oliver S, Peersman G, Thomas J. Review of Effectiveness of Sexual Health Promotion Interventions for Young People [Internet]. London: Unknown; 1995. Available: http://discovery.ucl.ac.uk/10015268/

290. Ogilvie D, Foster CE, Rothnie H, Cavill N, Hamilton V, Fitzsimons CF, et al. Interventions to promote walking: systematic review. Br Med J. 2007;334: 1204–1207. doi:10.1136/bmj.39198.722720.BE

291. Onate JA, Everhart JS, Clifton DR, Best TM, Borchers JR, Chaudhari AMW. Physical Exam Risk Factors for Lower Extremity Injury in High School Athletes: A Systematic Review. Clin J Sport Med. 2016;26: 435–444.

292. Onrust SA, Otten R, Lammers J, Smit F. School-based programmes to reduce and prevent substance use in different age groups: What works for whom? Systematic review and meta-regression analysis. Clin Psychol Rev. 2016;44: 45–59. doi:10.1016/j.cpr.2015.11.002

293. Oosterhoff M, Joore M, Ferreira I. The effects of school-based lifestyle interventions on body mass index and blood pressure: a multivariate multilevel meta-analysis of randomized controlled trials. Obes Rev Off J Int Assoc Study Obes. 2016;17: 1131–1153. doi:10.1111/obr.12446

294. Oringanje C, Meremikwu MM, Eko H, Esu E, Meremikwu A, Ehiri JE. Interventions for preventing unintended pregnancies among adolescents. Cochrane Database Syst Rev. 2009; CD005215. doi:10.1002/14651858.CD005215.pub2

295. Orton E, Whitehead J, Mhizha-Murira J, Clarkson M, Watson MC, Mulvaney CA, et al. School-based education programmes for the prevention of unintentional injuries in children and young people. Cochrane Database Syst Rev. 2016; doi:10.1002/14651858.CD010246.pub2

296. Owen J, Carroll C, Cooke J, Formby E, Hayter M, Hirst J, et al. School-linked sexual health services for young people (SSHYP): a survey and systematic review concerning current models, effectiveness, cost-effectiveness and research opportunities. Health Technol Assess Winch Engl. 2010;14: 1–228, iii–iv. doi:10.3310/hta14300

297. Owen MB, Curry WB, Kerner C, Newson L, Fairclough SJ. The effectiveness of school-based physical activity interventions for adolescent girls: A systematic review and meta-analysis. Prev Med. 2017;105: 237–249. doi:10.1016/j.ypmed.2017.09.018

298. Owen R, Kendrick D, Mulvaney C, Coleman T, Royal S. Non-legislative interventions for the promotion of cycle helmet wearing by children. Cochrane Database of Systematic Reviews. John Wiley & Sons, Ltd; 2011. Available: http://onlinelibrary.wiley.com/doi/10.1002/14651858.CD003985.pub3/abstract

299. Perez-Lopez JI, Tercedor Sanchez P, Delgado-Fernandez M. Effects of school-based physical activity and nutrition programs in Spanish adolescents: systematic review. Nutr Hosp. 2015;32: 534–544. doi:10.3305/nh.2015.32.2.9144

300. Pérez-Morales ME, Bacardí-Gascón M, Jiménez-Cruz A, Armendáriz-Anguiano A. [Randomized controlled school based interventions to prevent childhood obesity: systematic review from 2006 to 2009]. Arch Latinoam Nutr. 2009;59: 253–259.

301. Paleg G, Livingstone R. Outcomes of gait trainer use in home and school settings for children with motor impairments: a systematic review. Clin Rehabil. 2015;29: 1077–1091. doi:10.1177/0269215514565947

302. Park E. School-based smoking prevention programs for adolescents in South Korea: a systematic review. Health Educ Res. 2006;21: 407–415. doi:10.1093/her/cyl038

303. Parrish A-M, Okely AD, Stanley RM, Ridgers ND. The Effect of School Recess Interventions on Physical Activity. Sports Med. 2013;43: 287–299. doi:10.1007/s40279-013-0024-2

304. Passon AM, Gerber A, Schröer-Günther M. Wirksamkeit von schulbasierten Gruppeninterventionen zur Depressionsprävention. Kindh Entwickl. 2011;20: 236–246. doi:10.1026/0942-5403/a000061

305. Pattison S, Harris B. Adding value to education through improved mental health: A review of the research evidence on the effectiveness of counselling for children and young people. Aust Educ Res. 2006;33: 97–121.

306. Pearson M, Chilton R, Wyatt K, Abraham C, Ford T, Woods HB, et al. Implementing health promotion programmes in schools: a realist systematic review of research and experience in the United Kingdom. Implement Sci. 2015;10: 149. doi:10.1186/s13012-015-0338-6

307. Pearson N, Braithwaite R, Biddle SJH. The Effectiveness of Interventions to Increase Physical Activity Among Adolescent Girls: A Meta-analysis. Acad Pediatr. 2015;15: 9–18. doi:10.1016/j.acap.2014.08.009

308. Perman S, Turner S, Ramsay AIG, Baim-Lance A, Utley M, Fulop NJ. School-based vaccination programmes: a systematic review of the evidence on organisation and delivery in high income countries. Bmc Public Health. 2017;17: 252. doi:10.1186/s12889-017-4168-0

309. Persson TJ, Rousseau C. School-based interventions for minors in war-exposed countries: a review of targeted and general programmes. Torture Q J Rehabil Torture Vict Prev Torture. 2009;19: 88–101.

310. Petering R, Wenzel S, Winetrobe H. Systematic Review of Current Intimate Partner Violence Prevention Programs and Applicability to Homeless Youth. J Soc Soc Work Res. 2014;5: 107–135. doi:10.1086/675851

311. Picot J, Shepherd J, Kavanagh J, Cooper K, Harden A, Barnett-Page E, et al. Behavioural interventions for the prevention of sexually transmitted infections in young people aged 13-19 years: a systematic review. Health Educ Res. 2012;27: 495–512. doi:10.1093/her/cys014

312. Powell C, Wedner S, Hatt SR. Vision screening for correctable visual acuity deficits in school-age children and adolescents. Cochrane Database of Systematic Reviews. John Wiley & Sons, Ltd; 2004. Available: http://onlinelibrary.wiley.com/doi/10.1002/14651858.CD005023.pub2/abstract

313. Pozuelo Carrascosa D, García-Hermoso A, Alvarez-Bueno C, Sánchez-López M, Martinez Vizcaino V. Effectiveness of school-based physical activity programmes on cardiorespiratory fitness in children: A meta-analysis of randomised controlled trials. Br J Sports Med. 2017; doi:10.1136/bjsports-2017-097600

314. Pucher K k., Boot N m. w. m., De Vries N k. Systematic review: School health promotion interventions targeting physical activity and nutrition can improve academic performance in primary‐ and middle school children. Health Educ. 2013;113: 372–391. doi:10.1108/HE-02-2012-0013

315. Quitério ALD. School Physical Education: The Effectiveness of Health-Related Interventions and Recommendations for Health-Promotion Practice. Health Educ J. 2013;72: 716–732. doi:10.1177/0017896912460934

316. Rabin BA, Glasgow RE, Kerner JF, Klump MP, Brownson RC. Dissemination and Implementation Research on Community-Based Cancer Prevention: A Systematic Review. Am J Prev Med. 2010;38: 443–456. doi:10.1016/j.amepre.2009.12.035

317. Racey M, O’Brien C, Douglas S, Marquez O, Hendrie G, Newton G. Systematic Review of School-based Interventions to Modify Dietary Behavior: Does Intervention Intensity Impact Effectiveness? J Sch Health. 2016;86: 452–463. doi:10.1111/josh.12396

318. Rafferty R, Breslin G, Brennan D, Hassan D. A systematic review of school-based physical activity interventions on children’s wellbeing. Int Rev Sport Exerc Psychol. 2016;9: 215–230. doi:10.1080/1750984X.2016.1164228

319. Ran T, Chattopadhyay S, Hahn R. Economic Evaluation of School-Based Health Centers: A Community Guide Systematic Review. Value Health. 2016;19: A25–A25.

320. Raphael JL, Rueda A, Lion KC, Giordano TP. The Role of Lay Health Workers in Pediatric Chronic Disease: A Systematic Review. Acad Pediatr. 2013;13: 408–420.

321. Rasberry CN, Lee SM, Robin L, Laris BA, Russell LA, Coyle KK, et al. The association between school-based physical activity, including physical education, and academic performance: A systematic review of the literature. Prev Med. 2011;52: S10–S20. doi:10.1016/j.ypmed.2011.01.027

322. Renzaho AMN, Mellor D, Boulton K, Swinburn B. Effectiveness of prevention programmes for obesity and chronic diseases among immigrants to developed countries - a systematic review. Public Health Nutr. 2010;13: 438–450. doi:10.1017/S136898000999111X

323. Resnicow K. School‐based Obesity Prevention. Ann N Y Acad Sci. 1993;699: 154–166. doi:10.1111/j.1749-6632.1993.tb18847.x

324. Ribeiro IC, Parra DC, Hoehner CM, Soares J, Torres A, Pratt M, et al. School-based physical education programs: evidence-based physical activity interventions for youth in Latin America. Glob Health Promot. 2010;17: 5–15. doi:10.1177/1757975910365231

325. Rice K, Hiwi BT, Zwarenstein M, Lavallee B, Barre DE, Harris SB. Best Practices for the Prevention and Management of Diabetes and Obesity-Related Chronic Disease among Indigenous Peoples in Canada: A Review. Can J Diabetes. 2016;40: 216–225. doi:10.1016/j.jcjd.2015.10.007

326. Richards BS, Vitale MG. Screening for Idiopathic Scoliosis in Adolescents: An Information Statement*. J Bone Jt Surg-Am Vol. 2008;90: 195–198. doi:10.2106/JBJS.G.01276

327. Ring N, Malcolm C, Wyke S, Macgillivray S, Dixon D, Hoskins G, et al. Promoting the use of Personal Asthma Action Plans: a systematic review. Prim Care Respir J J Gen Pract Airw Group. 2007;16: 271–283. doi:10.3132/pcrj.2007.00049

328. Roberts IG, Kwan I. School‐based driver education for the prevention of traffic crashes. The Cochrane Library. John Wiley & Sons, Ltd; 2001. Available: http://onlinelibrary.wiley.com/doi/10.1002/14651858.CD003201/abstract

329. Robinson J, Cox G, Malone A, Williamson M, Baldwin G, Fletcher K, et al. A Systematic Review of School-Based Interventions Aimed at Preventing, Treating, and Responding to Suicide-Related Behavior in Young People. Crisis- J Crisis Interv Suicide Prev. 2013;34: 164–182. doi:10.1027/0227-5910/a000168

330. Rojas-Andrade R, Bahamondes LL. Is Implementation Fidelity Important? A Systematic Review on School-Based Mental Health Programs. Contemp Sch Psychol. 2018; 1–12. doi:10.1007/s40688-018-0175-0

331. Royal S, Kendrick D, Coleman T. Promoting bicycle helmet wearing by children using non-legislative interventions: systematic review and meta-analysis. Inj Prev. 2007;13: 162–167. doi:10.1136/ip.2006.013441

332. Sabirin J, Bakri R, Buang SN, Abdullah AT, Shapie A. School scoliosis screening programme-a systematic review. Med J Malaysia. 2010;65: 261–267.

333. Salerno JP. Effectiveness of Universal School-Based Mental Health Awareness Programs among Youth in the United States: A Systematic Review. J Sch Health. 2016;86: 922–931. doi:10.1111/josh.12461

334. Sancassiani F, Pintus E, Holte A, Paulus P, Moro MF, Cossu G, et al. Enhancing the Emotional and Social Skills of the Youth to Promote their Wellbeing and Positive Development: A Systematic Review of Universal School-based Randomized Controlled Trials. Clin Pract Epidemiol Ment Health CP EMH. 2015;11: 21–40. doi:10.2174/1745017901511010021

335. Sani AS, Abraham C, Denford S, Ball S. School-based sexual health education interventions to prevent STI/HIV in sub-Saharan Africa: a systematic review and meta-analysis. Bmc Public Health. 2016;16: 1069. doi:10.1186/s12889-016-3715-4

336. Saraf DS, Nongkynrih B, Pandav CS, Gupta SK, Shah B, Kapoor SK, et al. A Systematic Review of School-Based Interventions to Prevent Risk Factors Associated With Noncommunicable Diseases. Asia Pac J Public Health. 2012;24: 733–752. doi:10.1177/1010539512445053

337. Saraiya M, Glanz K, Briss PA, Nichols P, White C, Das D, et al. Interventions to prevent skin cancer by reducing exposure to ultraviolet radiation: a systematic review. Am J Prev Med. 2004;27: 422–466. doi:10.1016/j.amepre.2004.08.009

338. Sbruzzi G, Eibel B, Barbiero SM, Petkowicz RO, Ribeiro RA, Cesa CC, et al. Educational interventions in childhood obesity: a systematic review with meta-analysis of randomized clinical trials. Prev Med. 2013;56: 254–264. doi:10.1016/j.ypmed.2013.02.024

339. Schachter HM, Girardi A, Ly M, Lacroix D, Lumb AB, van Berkom J, et al. Effects of school-based interventions on mental health stigmatization: a systematic review. Child Adolesc Psychiatry Ment Health. 2008;2: 18. doi:10.1186/1753-2000-2-18

340. Scher LS, Maynard R, Stagner M. Interventions intended to reduce pregnancy-related outcomes among adolescents. Campbell Syst Rev. 2006;12.

341. Selkie EM, Fales JL, Moreno MA. Cyberbullying Prevalence Among US Middle and High School-Aged Adolescents: A Systematic Review and Quality Assessment. J Adolesc Health. 2016;58: 125–133. doi:10.1016/j.jadohealth.2015.09.026

342. Setiawan D, Oktora MP, Hutubessy R, Riewpaiboon A, Postma MJ. The health-economic studies of HPV vaccination in Southeast Asian countries: a systematic review. Expert Rev Vaccines. 2017;16: 933–943. doi:10.1080/14760584.2017.1357472

343. Sexton TL. The Relevance of Counseling Outcome Research: Current Trends and Practical Implications. J Couns Dev. 1996;74: 590–600. doi:10.1002/j.1556-6676.1996.tb02298.x

344. Shepherd J, Kavanagh J, Picot J, Cooper K, Harden A, Barnett-Page E, et al. The effectiveness and cost-effectiveness of behavioural interventions for the prevention of sexually transmitted infections in young people aged 13-19: a systematic review and economic evaluation. Health Technol Assess Winch Engl. 2010;14: 1–206, iii–iv. doi:10.3310/hta14070

345. Sherman EJ, Primack BA. What Works to Prevent Adolescent Smoking? A Systematic Review of the National Cancer Institute’s Research-Tested Intervention Programs. J Sch Health. 2009;79: 391–399. doi:10.1111/j.1746-1561.2009.00426.x

346. Shucksmith J (Janet), Jones SE (Susan), Summerbell CD (Carolyn). The role of parental involvement in school-based mental health interventions at primary (elementary) school level. Adv Sch Ment Health Promot. 2010; Available: https://tees.openrepository.com/tees/handle/10149/112762

347. Sichieri R, Cunha DB. Unbalanced Baseline in School-Based Interventions to Prevent Obesity: Adjustment Can Lead to Bias - a Systematic Review. Obes Facts. 2014;7: 221–232. doi:10.1159/000363438

348. Silveira JAC, Taddei JAAC, Guerra PH, Nobre MRC. Effectiveness of school-based nutrition education interventions to prevent and reduce excessive weight gain in children and adolescents: a systematic review. J Pediatr (Rio J). 2011;87: 382–392. doi:10.2223/JPED.2123

349. Cardoso da Silveira JA, de Aguiar Carrazedo Taddei JA, Guerra PH, Cuce Nobre MR. The effect of participation in school-based nutrition education interventions on body mass index: A meta-analysis of randomized controlled community trials. Prev Med. 2013;56: 237–243. doi:10.1016/j.ypmed.2013.01.011

350. Singh A, Uijtdewilligen L, Twisk JWR, van Mechelen W, Chinapaw MJM. Physical Activity and Performance at School A Systematic Review of the Literature Including a Methodological Quality Assessment. Arch Pediatr Adolesc Med. 2012;166: 49–55.

351. Sipsma HL, Jones KL, Cole-Lewis H. Breastfeeding among adolescent mothers: a systematic review of interventions from high-income countries. J Hum Lact Off J Int Lact Consult Assoc. 2015;31: 221–229; quiz 321–322. doi:10.1177/0890334414561264

352. Skeie MS, Klock KS. Dental caries prevention strategies among children and adolescents with immigrant - or low socioeconomic backgrounds- do they work? A systematic review. Bmc Oral Health. 2018;18: 20. doi:10.1186/s12903-018-0478-6

353. Sobol-Goldberg S, Rabinowitz J, Gross R. School-based obesity prevention programs: A meta-analysis of randomized controlled trials. Obesity. 2013;21: 2422–2428. doi:10.1002/oby.20515

354. Soole DW, Mazerolle L, Rombouts S. School-Based Drug Prevention Programs: A Review of What Works. Aust N Z J Criminol. 2008;41: 259–286. doi:10.1375/acri.41.2.259

355. Speizer IS, Magnani RJ, Colvin CE. The effectiveness of adolescent reproductive health interventions in developing countries: a review of the evidence. J Adolesc Health. 2003;33: 324–348. doi:10.1016/S1054-139X(02)00535-9

356. Stanley N, Ellis J, Farrelly N, Hollinghurst S, Downe S. Preventing domestic abuse for children and young people: A review of school-based interventions. Child Youth Serv Rev. 2015;59: 120–131. doi:10.1016/j.childyouth.2015.10.018

357. Stead M, Hastings G, Tudor-Smith C. Preventing adolescent smoking: a review of options. Health Educ J. 1996;55: 31–54. doi:10.1177/001789699605500105

358. Steele EJ, Dawson AP, Hiller JE. School-based interventions for spinal pain - A systematic review. Spine. 2006;31: 226–233. doi:10.1097/01.brs.0000195158.00680.0d

359. Stein C, Lopes Santos NM, Hilgert JB, Hugo FN. Effectiveness of oral health education on oral hygiene and dental caries in schoolchildren: Systematic review and meta-analysis. Community Dent Oral Epidemiol. 2018;46: 30–37. doi:10.1111/cdoe.12325

360. Stein C, Lopes Santos NM, Hilgert JB, Hugo FN. Effectiveness of oral health education on oral hygiene and dental caries in schoolchildren: Systematic review and meta-analysis. Community Dent Oral Epidemiol. 2018;46: 30–37. doi:10.1111/cdoe.12325

361. Stewart D, Wang D. Building resilience through school-based health promotion: a systematic review. Int J Ment Health Promot. 2012;14: 207–218. doi:10.1080/14623730.2013.770319

362. Stothard JR, Sousa-Figueiredo JC, Navaratnam AMD. Advocacy, policies and practicalities of preventive chemotherapy campaigns for African children with schistosomiasis. Expert Rev Anti Infect Ther. 2013;11: 733–752. doi:10.1586/14787210.2013.811931

363. Summerbell CD, Moore HJ, Vögele C, Kreichauf S, Wildgruber A, Manios Y, et al. Evidence-based recommendations for the development of obesity prevention programs targeted at preschool children. Obes Rev Off J Int Assoc Study Obes. 2012;13 Suppl 1: 129–132. doi:10.1111/j.1467-789X.2011.00940.x

364. Sun C, Pezic A, Tikellis G, Ponsonby A-L, Wake M, Carlin JB, et al. Effects of school-based interventions for direct delivery of physical activity on fitness and cardiometabolic markers in children and adolescents: a systematic review of randomized controlled trials. Obes Rev. 2013;14: 818–838. doi:10.1111/obr.12047

365. Suthar AB, Ford N, Bachanas PJ, Wong VJ, Rajan JS, Saltzman AK, et al. Towards Universal Voluntary HIV Testing and Counselling: A Systematic Review and Meta-Analysis of Community-Based Approaches. Plos Med. 2013;10: e1001496. doi:10.1371/journal.pmed.1001496

366. Sutton MY, Lasswell SM, Lanier Y, Miller KS. Impact of parent-child communication interventions on sex behaviors and cognitive outcomes for black/African-American and Hispanic/Latino youth: a systematic review, 1988-2012. J Adolesc Health Off Publ Soc Adolesc Med. 2014;54: 369–384. doi:10.1016/j.jadohealth.2013.11.004

367. Sutton BM, Webster AA, Westerveld MF. A systematic review of school-based interventions targeting social communication behaviors for students with autism. Autism Int J Res Pract. 2018; 1362361317753564. doi:10.1177/1362361317753564

368. Szumilas M, Kutcher S. Post-suicide intervention programs: a systematic review. Can J Public Health Rev Can Sante Publique. 2011;102: 18–29.

369. Taylor-Robinson DC, Maayan N, Soares-Weiser K, Donegan S, Garner P. Deworming drugs for soil-transmitted intestinal worms in children: effects on nutritional indicators, haemoglobin, and school performance. Cochrane Database of Systematic Reviews. John Wiley & Sons, Ltd; 2015. Available: http://onlinelibrary.wiley.com/doi/10.1002/14651858.CD000371.pub6/abstract

370. Teesson M, Newton NC, Barrett EL. Australian school-based prevention programs for alcohol and other drugs: A systematic review. Drug Alcohol Rev. 2012;31: 731–736. doi:10.1111/j.1465-3362.2012.00420.x

371. Thakore RV, Apfeld JC, Johnson RK, Sathiyakumar V, Jahangir AA, Sethi MK. School-based violence prevention strategy: a pilot evaluation. J Inj Violence Res. 2015;7: 45–53. doi:10.5249/jivr.v7i2.565

372. Thomas R. School-based programmes for preventing smoking. Cochrane Database Syst Rev. 2002; CD001293. doi:10.1002/14651858.CD001293

373. Thomas H, Ciliska D, Micucci S, Wilson-Abra J, Dobbins M. Effectiveness of physical activity enhancement and obesity prevention programs in children and youth. PubMed Health. 2004; Available: https://www.ncbi.nlm.nih.gov/pubmedhealth/PMH0021461/

374. Thomas R, Perera R. School-based programmes for preventing smoking. Cochrane Database Syst Rev. 2006; CD001293. doi:10.1002/14651858.CD001293.pub2

375. Thomas RE, McLellan J, Perera R. Effectiveness of school-based smoking prevention curricula: systematic review and meta-analysis. Bmj Open. 2015;5: e006976. doi:10.1136/bmjopen-2014-006976

376. Thomas MH. Abstinence-based programs for prevention of adolescent pregnancies. A review. J Adolesc Health Off Publ Soc Adolesc Med. 2000;26: 5–17.

377. Thomas RE, McLellan J, Perera R. School‐based programmes for preventing smoking. The Cochrane Library. John Wiley & Sons, Ltd; 2013. Available: http://onlinelibrary.wiley.com/doi/10.1002/14651858.CD001293.pub3/abstract

378. Tilford S, Delaney F, Meyrick J, Vogels M, Britain) HEA (Great. Effectiveness of mental health promotion interventions: a review [Internet]. London: Health Education Authority; 1997. Available: http://capitadiscovery.co.uk/dmu/items/439001

379. Tol WA, Barbui C, Galappatti A, Silove D, Betancourt TS, Souza R, et al. Mental health and psychosocial support in humanitarian settings: linking practice and research. Lancet Lond Engl. 2011;378: 1581–1591. doi:10.1016/S0140-6736(11)61094-5

380. Tollit M, Politis J, Knight S. Measuring School Functioning in Students With Chronic Fatigue Syndrome: A Systematic Review. J Sch Health. 2018;88: 74–89. doi:10.1111/josh.12580

381. Topping KJ, Barron IG. School-Based Child Sexual Abuse Prevention Programs: A Review of Effectiveness. Rev Educ Res. 2009;79: 431–463. doi:10.3102/0034654308325582

382. Ttofi MM, Farrington DP. Effectiveness of school-based programs to reduce bullying: a systematic and meta-analytic review. J Exp Criminol. 2011;7: 27–56. doi:10.1007/s11292-010-9109-1

383. Ttofi M, Farrington D. What works in preventing bullying: effective elements of anti‐bullying programmes. J Aggress Confl Peace Res. 2009;1: 13–24. doi:10.1108/17596599200900003

384. Turner C, McClure R, Nixon J, Spinks A. Community-based programs to promote car seat restraints in children 0-16 years -- a systematic review. Accid Anal Prev. 2005;37: 77–83. doi:10.1016/j.aap.2003.12.004

385. Uijtdewilligen L, Waters CN, Müller-Riemenschneider F, Lim YW. Preventing childhood obesity in Asia: an overview of intervention programmes. Obes Rev Off J Int Assoc Study Obes. 2016;17: 1103–1115. doi:10.1111/obr.12435

386. Underhill K, Montgomery P, Operario D. Abstinence‐plus programs for HIV infection prevention in high‐income countries. The Cochrane Library. John Wiley & Sons, Ltd; 2008. Available: http://onlinelibrary.wiley.com/doi/10.1002/14651858.CD007006/abstract

387. Underhill K, Operario D, Montgomery P. Abstinence‐only programs for HIV infection prevention in high‐income countries. The Cochrane Library. John Wiley & Sons, Ltd; 2007. Available: http://onlinelibrary.wiley.com/doi/10.1002/14651858.CD005421.pub2/abstract

388. Vezina-Im L-A, Beaulieu D, Belanger-Gravel A, Boucher D, Sirois C, Dugas M, et al. Efficacy of school-based interventions aimed at decreasing sugar-sweetened beverage consumption among adolescents: a systematic review. Public Health Nutr. 2017;20: 2416–2431. doi:10.1017/S1368980017000076

389. Van Cauwenberghe E, Maes L, Spittaels H, van Lenthe FJ, Brug J, Oppert J-M, et al. Effectiveness of school-based interventions in Europe to promote healthy nutrition in children and adolescents: systematic review of published and “grey” literature. Br J Nutr. 2010;103: 781–797. doi:10.1017/S0007114509993370

390. Van Lippevelde W, Verloigne M, De Bourdeaudhuij I, Brug J, Bjelland M, Lien N, et al. Does parental involvement make a difference in school-based nutrition and physical activity interventions? A systematic review of randomized controlled trials. Int J Public Health. 2012;57: 673–678. doi:10.1007/s00038-012-0335-3

391. van Sluijs EMF, McMinn AM, Griffin S. Effectiveness of interventions to promote physical activity in children and adolescents: systematic review of controlled trials. Bmj-Br Med J. 2007;335: 703–707. doi:10.1136/bmj.39320.843947.BE

392. van Sluijs EMF, McMinn AM, Griffin SJ. Effectiveness of interventions to promote physical activity in children and adolescents: systematic review of controlled trials. BMJ. 2007;335: 703. doi:10.1136/bmj.39320.843947.BE

393. van Stralen MM, Yildirim M, Velde SJT, Brug J, van Mechelen W, Chinapaw MJM. What works in school-based energy balance behaviour interventions and what does not? A systematic review of mediating mechanisms. Int J Obes. 2011;35: 1251–1265. doi:10.1038/ijo.2011.68

394. Verrotti A, Penta L, Zenzeri L, Agostinelli S, De Feo P. Childhood obesity: prevention and strategies of intervention. A systematic review of school-based interventions in primary schools. J Endocrinol Invest. 2014;37: 1155–1164. doi:10.1007/s40618-014-0153-y

395. Verstraeten R, Roberfroid D, Lachat C, Leroy JL, Holdsworth M, Maes L, et al. Effectiveness of preventive school-based obesity interventions in low- and middle-income countries: a systematic review. Am J Clin Nutr. 2012;96: 415–438. doi:10.3945/ajcn.112.035378

396. Villa-Gonzalez E, Barranco-Ruiz Y, Evenson KR, Chillon P. Systematic review of interventions for promoting active school transport. Prev Med. 2018;111: 115–134. doi:10.1016/j.ypmed.2018.02.010

397. Vindigni SM, Riley PL, Jhung M. Systematic review: handwashing behaviour in low- to middle-income countries: outcome measures and behaviour maintenance. Trop Med Int Health TM IH. 2011;16: 466–477. doi:10.1111/j.1365-3156.2010.02720.x

398. Vreeman RC, Carroll AE. A systematic review of school-based interventions to prevent bullying. Arch Pediatr Adolesc Med. 2007;161: 78–88. doi:10.1001/archpedi.161.1.78

399. Waddell C, Hua JM, Garland OM, DeV PR, McEwan K. Preventing mental disorders in children: A systematic review to inform policy-making. Can J Public Health. 2007;98: 166–73. doi:10.17269/cjph.98.812

400. Wainwright P, Thomas J, Jones M. Health promotion and the role of the school nurse: a systematic review. J Adv Nurs. 2000;32: 1083–1091. doi:10.1046/j.1365-2648.2000.01579.x

401. Walsh K, Zwi K, Woolfenden S, Shlonsky A. School-Based Education Programs for the Prevention of Child Sexual Abuse: A Cochrane Systematic Review and Meta-Analysis. Res Soc Work Pract. 2018;28: 33–55. doi:10.1177/1049731515619705

402. Walsh K, Zwi K, Woolfenden S, Shlonsky A. School‐based education programmes for the prevention of child sexual abuse. The Cochrane Library. John Wiley & Sons, Ltd; 2015. Available: http://onlinelibrary.wiley.com/doi/10.1002/14651858.CD004380.pub3/abstract

403. Walter H, Sadeque-Iqbal F, Ulysse R, Castillo D, Fitzpatrick A, Singleton J. The effectiveness of school-based family asthma educational programs on the quality of life and number of asthma exacerbations of children aged five to 18 years diagnosed with asthma: a systematic review protocol. JBI Database Syst Rev Implement Rep. 2015;13: 69–81. doi:10.11124/jbisrir-2015-2335

404. Wang D, Stewart D. The implementation and effectiveness of school-based nutrition promotion programmes using a health-promoting schools approach: a systematic review. Public Health Nutr. 2013;16: 1082–1100. doi:10.1017/S1368980012003497

405. Wang T, Lurie M, Govindasamy D, Mathews C. The Effects of School-Based Condom Availability Programs (CAPs) on Condom Acquisition, Use and Sexual Behavior: A Systematic Review. Aids Behav. 2018;22: 308–320. doi:10.1007/s10461-017-1787-5

406. Wang Y, Cai L, Wu Y, Wilson RF, Weston C, Fawole O, et al. What childhood obesity prevention programmes work? A systematic review and meta-analysis. Obes Rev Off J Int Assoc Study Obes. 2015;16: 547–565. doi:10.1111/obr.12277

407. Wang T, Lurie M, Govindasamy D, Mathews C. The Effects of School-Based Condom Availability Programs (CAPs) on Condom Acquisition, Use and Sexual Behavior: A Systematic Review. Aids Behav. 2018;22: 308–320. doi:10.1007/s10461-017-1787-5

408. Warren-Gash C, Fragaszy E, Hayward AC. Hand hygiene to reduce community transmission of influenza and acute respiratory tract infection: a systematic review. Influenza Other Respir Viruses. 2013;7: 738–749. doi:10.1111/irv.12015

409. Waters E, de Silva-Sanigorski A, Burford BJ, Brown T, Campbell KJ, Gao Y, et al. Interventions for preventing obesity in children. Cochrane Database of Systematic Reviews. John Wiley & Sons, Ltd; 2011. Available: http://onlinelibrary.wiley.com/doi/10.1002/14651858.CD001871.pub3/abstract

410. Watson JA, Ensink JHJ, Ramos M, Benelli P, Holdsworth E, Dreibelbis R, et al. Does targeting children with hygiene promotion messages work? The effect of handwashing promotion targeted at children, on diarrhoea, soil-transmitted helminth infections and behaviour change, in low- and middle-income countries. Trop Med Int Health. 2017;22: 526–538. doi:10.1111/tmi.12861

411. Weisz, Weiss B*, Alicke MD*, Klotz ML*. Effectiveness of psychotherapy with children and adolescents: A meta-analysis for clinicians. J Consult Clin Psychol. 1987;55: 542–549.

412. Wells J, Barlow J, Stewart‐Brown S. A systematic review of universal approaches to mental health promotion in schools. Health Educ. 2003;103: 197–220. doi:10.1108/09654280310485546

413. Werch CE, Owen DM. Iatrogenic effects of alcohol and drug prevention programs [Internet]. Centre for Reviews and Dissemination (UK); 2002. Available: https://www.ncbi.nlm.nih.gov/books/NBK69521/

414. Westwood M, Fayter D, Hartley S, Rithalia A, Butler G, Glasziou P, et al. Childhood obesity: should primary school children be routinely screened? A systematic review and discussion of the evidence. Arch Dis Child. 2007;92: 416–422. doi:10.1136/adc.2006.11.2589

415. Whitaker DJ, Morrison S, Lindquist C, Hawkins SR, O’Neil JA, Nesius AM, et al. A critical review of interventions for the primary prevention of perpetration of partner violence. Aggress Violent Behav. 2006;11: 151–166. doi:10.1016/j.avb.2005.07.007

416. Whitaker D, Murphy, Eckhardt C, Hodges, Osborne M. Effectiveness of Primary Prevention Efforts for Intimate Partner Violence. Partn Abuse. 2013;4: 175–195. doi:10.1891/1946-6560.4.2.175

417. White D, Pitts M. Educating young people about drugs: a systematic review. Addict Abingdon Engl. 1998;93: 1475–1487.

418. Wiefferink CH, Peters L, Hoekstra F, Dam GT, Buijs GJ, Paulussen TGWM. Clustering of health-related behaviors and their determinants: possible consequences for school health interventions. Prev Sci Off J Soc Prev Res. 2006;7: 127–149. doi:10.1007/s11121-005-0021-2

419. Wiehe SE, Garrison MM, Christakis DA, Ebel BE, Rivara FP. A systematic review of school-based smoking prevention trials with long-term follow-up. J Adolesc Health Off Publ Soc Adolesc Med. 2005;36: 162–169. doi:10.1016/j.jadohealth.2004.12.003

420. Wight D, Fullerton D. A Review of Interventions With Parents to Promote the Sexual Health of Their Children. J Adolesc Health. 2013;52: 4–27. doi:10.1016/j.jadohealth.2012.04.014

421. Willmott M, Nicholson A, Busse H, MacArthur GJ, Brookes S, Campbell R. Effectiveness of hand hygiene interventions in reducing illness absence among children in educational settings: a systematic review and meta-analysis. Arch Dis Child. 2016;101: 42–50. doi:10.1136/archdischild-2015-308875

422. Wilson S, Lipsey M. The Effects of School-Based Social Information Processing Interventions on Aggressive Behavior, Part II: Selected/Indicated Pull-Out Programs. Campbell Syst Rev. 2006;6.

423. Wilson S, Lipsey M. The Effects of School-Based Social Information Processing Interventions on Aggressive Behaviour: Part I: Universal Programs. Campbell Syst Rev. 2007;

424. Wood E, Shakeshaft A, Gilmour S, Sanson-Fisher R. A systematic review of school-based studies involving alcohol and the community. Aust N Z J Public Health. 2006;30: 541–549. doi:10.1111/j.1467-842X.2006.tb00783.x

425. Wood S, Mayo-Wilson E. School-Based Mentoring for Adolescents: A Systematic Review and Meta-Analysis. Res Soc Work Pract. 2012;22: 257–269. doi:10.1177/1049731511430836

426. Worrell K, Shaw MR, Postma J, Katz JR. A Systematic Review of the Literature on Screening for Exercise-Induced Asthma: Considerations for School Nurses. J Sch Nurs. 2015;31: 70–76. doi:10.1177/1059840514523295

427. Yamada J, DiCenso A, Feldman L, Cormillott P, Wade K, Wignall R, et al. A systematic review of the effectiveness of primary prevention programs to prevent sexually transmitted diseases in adolescents [Internet]. Dundas, ON, Canada: Ontario Ministry of Health, region of Hamilton-Wentworth, Social and Public Health Services Division. Effective Public Health Practice Project.; 1999. Available: https://extranet.who.int/rhl/topics/adolescent-sexual-and-reproductive-health/sexually-transmitted-infections-including-hiv/systematic-review-effectiveness-primary-prevention-programs-prevent-sexually-transmitted-diseases

428. Yeung CA, Chong LY, Glenny A-M. Fluoridated milk for preventing dental caries. The Cochrane Library. John Wiley & Sons, Ltd; 2015. Available: http://onlinelibrary.wiley.com/doi/10.1002/14651858.CD003876.pub4/abstract

429. Yildirim M, Van Stralen MM, Chinapaw MJM, Brug J, Van Mechelen W, Twisk JWR, et al. For whom and under what circumstances do school-based energy balance behavior interventions work? Systematic review on moderators. Int J Pediatr Obes. 2011;6: E46–E57. doi:10.3109/17477166.2011.566440

430. Young I, Waddell L, Harding S, Greig J, Mascarenhas M, Sivaramalingam B, et al. A systematic review and meta-analysis of the effectiveness of food safety education interventions for consumers in developed countries. BMC Public Health. 2015;15: 822. doi:10.1186/s12889-015-2171-x

431. Zwi KJ, Woolfenden SR, Wheeler DM, O’brien TA, Tait P, Williams KW. School-based education programmes for the prevention of child sexual abuse. Cochrane Database Syst Rev. 2007; CD004380. doi:10.1002/14651858.CD004380.pub2

432. Arora A, Khattri S, Ismail NM, Kumbargere Nagraj S, Prashanti E. School dental screening programmes for oral health. The Cochrane Library. John Wiley & Sons, Ltd; 2017. Available: http://cochranelibrary-wiley.com/doi/10.1002/14651858.CD012595.pub2/full

433. Higgins J, Altman D, Sterne J, editors. Chapter 8: Assessing risk of bias in included studies. Cochrane Handbook for Systematic Reviews of Interventions Version 510 (updated March 2011). The Cochrane Collaboration; 2011. Available: handbook.cochrane.org

434. Bastounis A, Callaghan P, Banerjee A, Michail M. The effectiveness of the Penn Resiliency Programme (PRP) and its adapted versions in reducing depression and anxiety and improving explanatory style: A systematic review and meta-analysis. J Adolesc. 2016;52: 37–48. doi:10.1016/j.adolescence.2016.07.004

435. Higgins J, Altman D. Assessing risk of bias in included studies. Cochrane handbook for systematic reviews of interventions Cochrane book series. Chichester, UK: Wiley-Blackwell; 2008.

436. Brendel KE, Maynard BR, Albright DL, Bellomo M. Effects of School-Based Interventions with U.S. Military-Connected Children: A Systematic Review. Res Soc Work Pract. 2014;24: 649–658. doi:10.1177/1049731513517143

437. Chung K-F, Chan M-S, Lam Y-Y, Lai CS-Y, Yeung W-F. School-Based Sleep Education Programs for Short Sleep Duration in Adolescents: A Systematic Review and Meta-Analysis. J Sch Health. 2017;87: 401–408. doi:10.1111/josh.12509

438. Cooper AM, O’Malley LA, Elison SN, Armstrong R, Burnside G, Adair P, et al. Primary school-based behavioural interventions for preventing caries. Cochrane Database Syst Rev. 2013; CD009378. doi:10.1002/14651858.CD009378.pub2

439. Evans JR, Morjaria P, Powell C. Vision screening for correctable visual acuity deficits in school‐age children and adolescents. The Cochrane Library. John Wiley & Sons, Ltd; 2018. Available: http://cochranelibrary-wiley.com/doi/10.1002/14651858.CD005023.pub3/full

440. Geryk LL, Roberts CA, Carpenter DM. A systematic review of school-based interventions that include inhaler technique education. Respir Med. 2017;132: 21–30. doi:10.1016/j.rmed.2017.09.001

441. Gold C, Wigram T, Elefant C. Music therapy for autistic spectrum disorder. The Cochrane Library. John Wiley & Sons, Ltd; 2006. Available: http://cochranelibrary-wiley.com/doi/10.1002/14651858.CD004381.pub2/abstract

442. Hennegan J, Montgomery P. Do Menstrual Hygiene Management Interventions Improve Education and Psychosocial Outcomes for Women and Girls in Low and Middle Income Countries? A Systematic Review. Plos One. 2016;11: e0146985. doi:10.1371/journal.pone.0146985

443. EPOC Resources for review authors. Oslo: Norwegian Knowledge Centre for the Health Services [Internet]. Effective Practice and Organisation of Care Group (EPOC); 2013. Available: http://epocoslo. cochrane.org/epoc-specific-resources-review-authors

444. Higgins E, O’Sullivan S. “What Works”: systematic review of the “FRIENDS for Life” programme as a universal school-based intervention programme for the prevention of child and youth anxiety. Educ Psychol Pract. 2015;31: 424–438. doi:10.1080/02667363.2015.1086977

445. Kavanagh J, Oliver S, Lorenc T, Caird J, Tucker H, Harden A, et al. School-based cognitive-behavioural interventions: A systematic review of effects and inequalities. Health Sociol Rev. 2009;18: 61–78. doi:10.5172/hesr.18.1.61

446. Peersman G, Oliver S, Oakley A. EPPI-Centre Review Guidelines. London: EPPI-Centre, Social Science Research Unit, Institute of Education; 1997.

447. Marinho VC, Worthington HV, Walsh T, Chong LY. Fluoride gels for preventing dental caries in children and adolescents. The Cochrane Library. John Wiley & Sons, Ltd; 2015. Available: http://onlinelibrary.wiley.com/doi/10.1002/14651858.CD002280.pub2/abstract

448. McDonald A, Drey N. Primary-school-based Art Therapy: A Review of Controlled Studies. Int J Art Ther. 2017;23: 33–44.

449. Downs SH, Black N. The feasibility of creating a checklist for the assessment of the methodological quality both of randomised and non-randomised studies of health care interventions. J Epidemiol Community Health. 1998;52: 377–384.

450. Neil AL, Christensen H. Efficacy and effectiveness of school-based prevention and early intervention programs for anxiety. Clin Psychol Rev. 2009;29: 208–215. doi:10.1016/j.cpr.2009.01.002

451. Jadad AR, Moore RA, Carroll D, Jenkinson C, Reynolds DJ, Gavaghan DJ, et al. Assessing the quality of reports of randomized clinical trials: is blinding necessary? Control Clin Trials. 1996;17: 1–12.

452. Paul-Ebhohimhen VA, Poobalan A, van Teijlingen ER. A systematic review of school-based sexual health interventions to prevent STI/HIV in sub-Saharan Africa. BMC Public Health. 2008;8: 4. doi:10.1186/1471-2458-8-4

453. Schroeder K, Travers J, Smaldone A. Are School Nurses an Overlooked Resource in Reducing Childhood Obesity? A Systematic Review and Meta-analysis. J Sch Health. 2016;86: 309–321. doi:10.1111/josh.12386

454. Sullivan AL, Simonson GR. A Systematic Review of School-Based Social-Emotional Interventions for Refugee and War-Traumatized Youth. Rev Educ Res. 2016;86: 503–530. doi:10.3102/0034654315609419

455. Walter H, Sadeque-Iqbal F, Ulysse R, Castillo D, Fitzpatrick A, Singleton J. Effectiveness of school-based family asthma educational programs in quality of life and asthma exacerbations in asthmatic children aged five to 18: a systematic review. JBI Database Syst Rev Implement Rep. 2016;14: 113–138. doi:10.11124/JBISRIR-2016-003181

456. Werner-Seidler A, Perry Y, Calear AL, Newby JM, Christensen H. School-based depression and anxiety prevention programs for young people: A systematic review and meta-analysis. Clin Psychol Rev. 2017;51: 30–47. doi:10.1016/j.cpr.2016.10.005

457. Shea BJ, Reeves BC, Wells G, Thuku M, Hamel C, Moran J, et al. AMSTAR 2: a critical appraisal tool for systematic reviews that include randomised or non-randomised studies of healthcare interventions, or both. The BMJ. 2017;358. doi:10.1136/bmj.j4008
